# Diversity of dirammox microorganisms

**DOI:** 10.1128/mbio.01645-26

**Published:** 2026-10-15

**Authors:** Gantuya Khasbaatar, Qun Han, Xiao-Kang Wang, Lei Song, Chang Liu, Xin Sun, Ze-Shen Liu, Ying Liu, Cheng-Ying Jiang, Ji-Guo Qiu, Shuang-Jiang Liu, Yanning Zheng, De-Feng Li

**Affiliations:** 1 State Key Laboratory of Microbial Diversity and Innovative Utilization, Institute of Microbiology, Chinese Academy of Sciences 12381, Beijing, China; 2 College of Life Sciences, University of Chinese Academy of Sciences 74519, Beijing, China; 3 China General Microbiological Culture Collection Center, Institute of Microbiology, Chinese Academy of Sciences 12381, Beijing, China; 4 Key Laboratory of Agricultural and Environmental Microbiology, Ministry of Agriculture and Rural Affairs, College of Life Sciences, Nanjing Agricultural University 70578, Nanjing, China; The University of Oklahoma, Norman, Oklahoma, USA

**Keywords:** direct ammonia oxidation, dirammox, DnfABC, heterotrophic nitrifier, heterotrophic nitrification

## Abstract

Our knowledge of the nitrogen cycle has been advanced by the discovery of anammox bacteria, ammonia-oxidizing archaea, and comammox bacteria, along with their high diversity. Direct ammonia oxidation (dirammox), a recently discovered heterotrophic nitrification pathway, converts ammonium and organic nitrogen directly to N_2_ through the DnfABC multienzyme system, further expanding our understanding of the nitrogen cycle. However, the taxonomic distribution, prevalence, and nitrogen metabolic potential of microorganisms encoding dirammox genes remain poorly understood. Here, large-scale genome mining identified complete *dnfABC* gene clusters in 690 genomes representing 3 phyla and 26 genera. The *dnfABC* cluster showed a striking bimodal prevalence: it was nearly ubiquitous in *Alcaligenes*, *Delftia*, and *Microvirgula* but occurred sporadically in most other genera. These microorganisms were recovered from diverse environments, particularly organic nitrogen-rich habitats. Comparative analyses of DnfABC phylogeny, gene cluster organization, GC content, and flanking mobile genetic elements indicate that the *dnfABC* cluster is conserved within several core genera, whereas its sporadic distribution in other lineages suggests that horizontal gene transfer may have contributed to its dissemination. The *dnfABC* cluster co-occurs with genes associated with assimilatory nitrate/nitrite reduction to ammonium, dissimilatory nitrate/nitrite reduction to ammonium, denitrification, and/or nitrogen fixation, but not with marker genes for nitrification or anammox, indicating diverse nitrogen metabolic potential among potential dirammox microorganisms. Functional assays further showed that diverse *dnfABC* clusters consistently conferred ammonium- and glutamine-oxidizing activities in *Escherichia coli*, despite exhibiting low activities in their native hosts. These results suggest that dirammox is a broadly distributed, metabolically flexible, and tightly regulated pathway and may represent an overlooked route of nitrogen transformation in organic-rich habitats.

**IMPORTANCE:**

Nitrogen is essential for all life, and our understanding of the nitrogen cycle has advanced substantially with the discovery of anammox bacteria, ammonia-oxidizing archaea, and comammox bacteria, which exhibit remarkable taxonomic and metabolic diversity. This study suggests that the recently discovered dirammox pathway may be more widespread in nature than previously recognized. Potential dirammox microorganisms exhibit broad phylogenetic and ecological distributions, high genomic diversity, and notable metabolic versatility. Functional assays indicate that dirammox activity is tightly regulated in native hosts, suggesting that its ecological role depends on specific environmental conditions. Unlike canonical nitrification and anammox, dirammox operates under conditions where other nitrogen transformations are limited, highlighting an important route of nitrogen loss in organic-rich habitats. These findings highlight the previously overlooked potential role of dirammox in nitrogen cycling, expand our understanding of microbial contributions to the global nitrogen cycle, and provide a foundation for future ecological and environmental research.

## INTRODUCTION

The nitrogen cycle is a fundamental biogeochemical process that regulates nitrogen availability and maintains ecosystem balance. This cycle comprises multiple microbially mediated transformations, including nitrogen fixation, nitrification, denitrification, and anaerobic ammonium oxidation (anammox) (1–8). The discovery of ammonia-oxidizing archaea and comammox bacteria, along with their high diversity, has substantially advanced our knowledge of the nitrogen cycle (9–12).

Furthermore, recent studies have identified a previously unrecognized heterotrophic ammonia oxidation pathway, termed dirammox (direct ammonia oxidation), in heterotrophic nitrifiers *Alcaligenes ammonioxydans* HO-1 and *Alcaligenes faecalis* JQ135, which contributes to the predominant N_2_ production from ammonium during heterotrophic nitrification by *Alcaligenes* (13–15). In detail, ammonium and organic nitrogen compounds were converted into glutamine via glutamine synthetase GlnA, which was then oxidized to hydroxylamine and subsequently to N_2_ via the DnfABC multienzyme system (13, 16). The DnfA, DnfB, and DnfC show the highest amino acid sequence identity with 4-aminobenzoate N-oxygenase CmlI from *Streptomyces venezuelae*, toluene-4-sulfonate monooxygenase system reductase subunit from *Comamonas testosteroni*, and glutamine amidotransferase-like protein YfeJ from *Salmonella enterica* in the UniProtKB/Swiss-Prot database, respectively. DnfA has been shown to bind hydroxylamine and oxidize it to N_2_ *in vitro* and was proposed to be responsible for the conversion of almost 50% of ammonium to N_2_ *in vivo* (17). We therefore proposed that DnfC converts glutamine to an unknown amine, and DnfAB oxidizes that amine to hydroxylamine, which is then converted to N_2_ (13, 16). In contrast, Miao et al. proposed that glutamine was oxidized to L-glutamic acid γ-hydroxamate by DnfAB, hydrolyzed to hydroxylamine by DnfC, and finally oxidized to N_2_ by DnfAB (18). Lenferink et al. confirmed the production of hydroxylamine from ammonium by *Alcaligenes faecalis*, and observed the abiotic, not biotic, conversion of hydroxylamine to N_2_ under the conditions tested (19). Although the detailed mechanism of converting ammonium/organic nitrogen compounds to N_2_ remains controversial, the discovery of dirammox revealed a pathway for both the aerobic oxidation of ammonium and organic nitrogen compounds and the aerobic production of N_2_, thereby updating our understanding of heterotrophic nitrification.

The knockout of any gene in *dnfABC* abolishes the ability of *Alcaligenes* to produce hydroxylamine and N_2_, and the *dnfABC* gene cluster confers upon *Escherichia coli* cells the ability to oxidize ammonium and organic nitrogen to hydroxylamine and N_2_ (13, 14). These findings indicate that the *dnfABC* gene cluster is required for the dirammox process in *Alcaligenes* and is sufficient for it in *E. coli* cells and possibly other microorganisms. Therefore, investigating the presence of the *dnfABC* gene cluster in microbial taxa is important for further understanding the physiological and ecological roles of potential dirammox microorganisms. Hou et al. found that 36 of 49 *Alcaligenes* genomes encoded the *dnfABC* gene cluster and observed that 14 of 16 *Alcaligenes* strains oxidized ammonium to hydroxylamine (20). Xu et al. identified the *dnfABC* gene cluster in many gram-negative bacteria, including 108 strains from *Andreprevotia*, *Burkholderia*, *Delftia*, *Duganella*, *Herbaspirillum*, *Jeongeupia*, *Microvirgula*, *Pokkaliibacter*, and *Pseudomonas* (14). Miao et al. reported the presence of the *dnfABC* gene cluster in *Pseudomonas*, *Delftia*, and *Microvirgula*, observed that *Delftia acidovorans* CGMCC1.3363T, *Delftia* sp. CGMCC1.18821T, *Pseudomonas parafulva* CGMCC1.5634, and *Microvirgula* sp. CGMCC1.19429T could convert glutamine to N_2_, and it was confirmed that these *dnfABC* gene clusters enabled *E. coli* cells to produce N_2_ (18). These studies indicate that the *dnfABC* gene cluster may be more widely distributed across microbial taxa than previously recognized.

However, the taxonomic breadth, prevalence, and diversity of potential dirammox microorganisms remain largely unexplored. Here, we present a comprehensive survey of the taxonomic distribution and prevalence of complete *dnfABC* gene clusters using genome-scale bioinformatic analysis and assess the diversity of microorganisms with genetically predicted dirammox potential. Our study expands current knowledge of this recently identified heterotrophic nitrification pathway and provides a foundation for future investigations into its physiological functions, ecological significance, and potential applications in wastewater treatment.

## RESULTS

### Taxonomic breadth and bimodal prevalence of the *dnfABC* gene cluster

**Fig 1:**
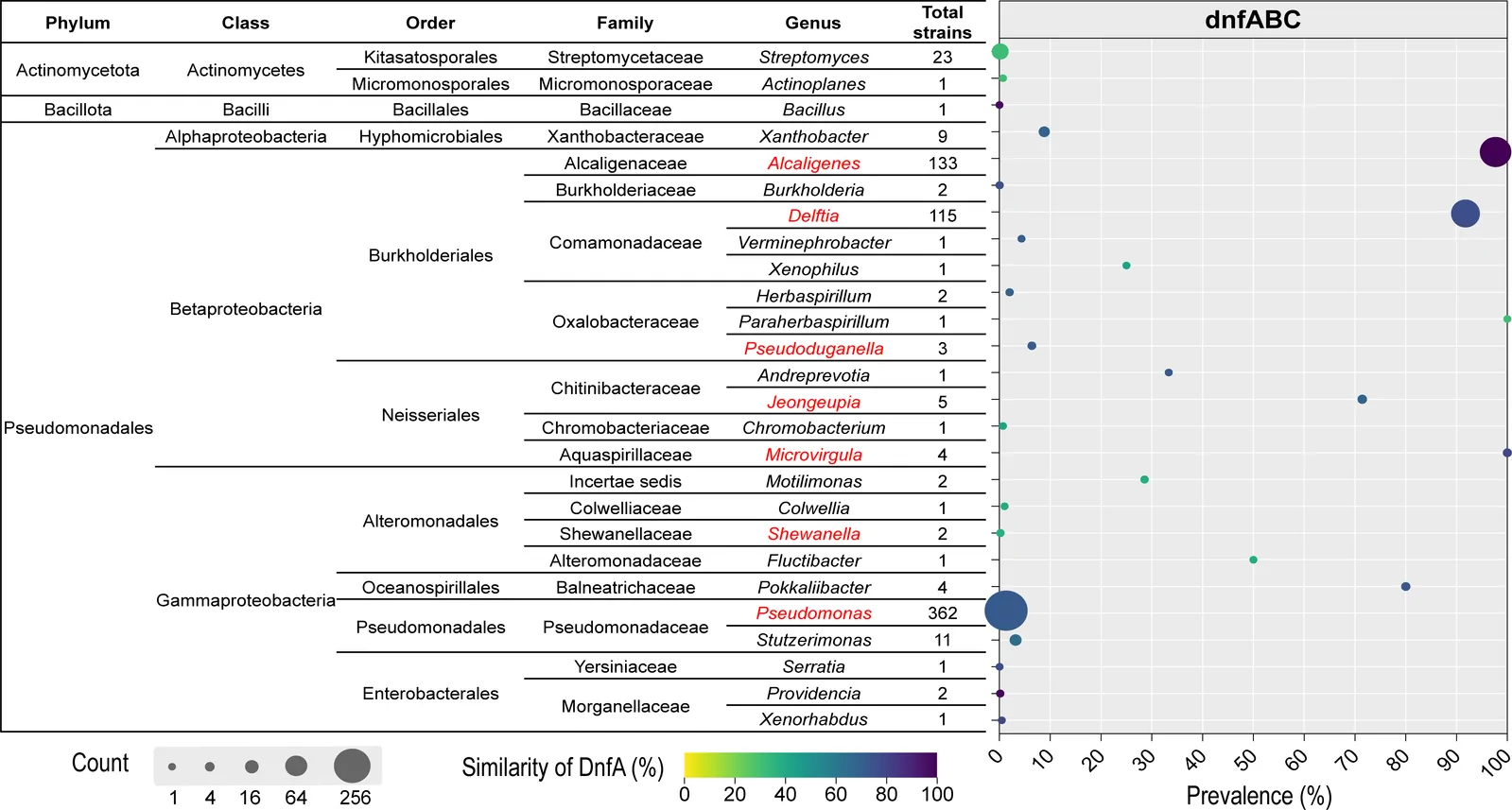
Taxonomic diversity of potential dirammox microorganisms. The left panel displays the hierarchical classification and the total number of *dnfABC* cluster gene-harboring strains in each genus. The right bubble plot illustrates the prevalence (%) on the *x*-axis, with bubble size indicating the number of strains and color representing DnfA sequence similarity to *A. faecalis* JQ135. Red genus names indicate genera represented by experimentally tested strains.

To identify potential dirammox microorganisms, we screened publicly available bacterial genomes for the presence of the *dnfABC* gene cluster, a genetic hallmark of the dirammox pathway. We identified 690 genomes containing complete *dnfABC* gene clusters using hidden Markov model (HMM)-based screening and cluster confirmation. These bacteria belong to 3 phyla, 5 classes, 10 orders, 19 families, and 26 genera, including *Actinoplanes*, *Alcaligenes*, *Andreprevotia*, *Bacillus*, *Burkholderia*, *Chromobacterium*, *Colwellia*, *Delftia*, *Fluctibacter*, *Herbaspirillum*, *Jeongeupia*, *Microvirgula*, *Motilimonas*, *Paraherbaspirillum*, *Pokkaliibacter*, *Providencia*, *Pseudoduganella*, *Pseudomonas*, *Serratia*, *Shewanella*, *Streptomyces*, *Stutzerimonas*, *Verminephrobacter*, *Xanthobacter*, *Xenophilus*, and *Xenorhabdus* (Fig. 1). The top three genera are *Pseudomonas*, *Alcaligenes*, and *Delftia*, which consist of 88.4% (610 strains) of target strains and contain 362, 133, and 115 strains, respectively. Of all strains, 96.4% (665 strains from 23 genera) belong to the phylum Proteobacteria. In contrast, 24 strains are classified under Actinobacteria and one strain under Bacillota (Fig. 1). The 690 strains were isolated from a wide range of environments, including animal-associated habitats (85 strains, 12.3%), plant-associated habitats (107, 15.5%), clinical settings (63, 9.1%), extreme environments (25, 3.6%), hospital and indoor environments (78, 11.3%), freshwater environments (69, 10.0%), marine environments (16, 2.3%), sewage systems (38, 5.5%), soils (86, 12.5%), other environments (92, 13.3%), and unknown sources (31, 4.5%; Fig. S1; Table S3). These genomic findings suggest that genetically predicted dirammox microorganisms are broadly distributed across different genera, inhabit diverse terrestrial and aquatic habitats, occupy diverse ecological niches, and may preferentially occur in organic nitrogen-rich habitats.

Although potential dirammox microorganisms are taxonomically widespread, their prevalence differs markedly among genera, as measured by the percentage of sequenced genomes carrying *dnfABC* within each genus. Approximately half of the strains were identified as *Pseudomonas* (362 strains), yet they constitute only 1.6% of the publicly available sequenced *Pseudomonas* genomes, indicating a very low prevalence of *dnfABC* in *Pseudomonas*. In contrast, *Alcaligenes* and *Delftia*, with 133 and 115 strains, respectively, exhibited substantially higher prevalence, representing 97.8% and 92.7% of their sequenced genomes, respectively. Although the total number of available genomes was limited for *Fluctibacter* (1/2), *Jeongeupia* (5/7 strains), *Pokkaliibacter* (4/5), and *Microvirgula* (4/4), the prevalence of *dnfABC* in these genera was relatively high, reaching 50.0%, 71.4%, 80.0%, and 100.0%, respectively. Only one *Paraherbaspirillum* genome was sequenced, which also encoded *dnfABC*. However, these estimates should be interpreted cautiously because of the small sample sizes. The prevalence of *dnfABC* in other genera was very low, at less than 33.3% (Fig. 1). Taken together, the *dnfABC* gene cluster exhibits a bimodal distribution across genera, being highly prevalent (92.7%–100%) in three genera, i.e., *Alcaligenes*, *Delftia*, and *Microvirgula*, but sporadically present (<33% prevalence) in most of the remaining genera. This distribution pattern suggests that the *dnfABC* gene cluster is genetically conserved in several genera, including *Alcaligenes*, *Delftia*, and *Microvirgula*, whereas its presence in other genera may be consistent with horizontal transfer.

### The architectural diversity of *dnfABC* gene clusters is associated with gene rearrangements and inversions

**Fig 2:**
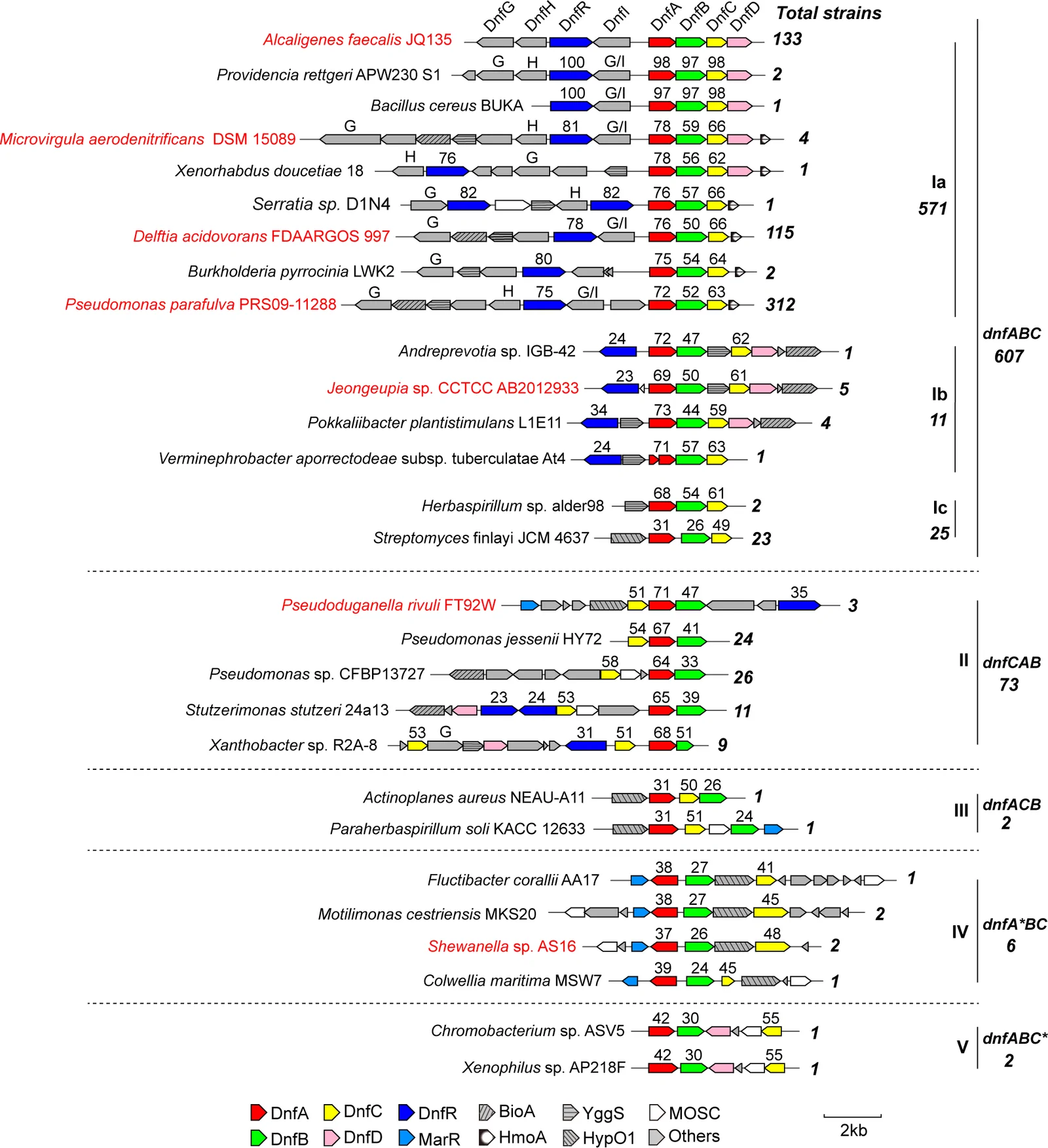
The *dnfABC* gene cluster organizations across the representative genomes of 26 genera. The clusters are classified into five distinct groups based on the ORF order and orientation: group I (subdivided into Ia, Ib, and Ic; *dnfABC*), group II (*dnfCAB*), group III (*dnfACB*), group IV (dnfA*BC, * reverse direction), and group V (dnfABC*). Colored arrows indicate different genes or functional elements: *dnfA* (red), *dnfB* (green), *dnfC* (yellow), *dnfD* (pink), *dnfR* (dark blue), *MarR* (light blue), and others (gray). Sequence identities (%) of DnfABC proteins relative to *A. faecalis* JQ135 are shown above each ORF. The total number of strains was denoted alongside the representative species. The A 2-kb scale bar represents the relative size of the genomic regions. A stop codon was identified in the *dnfA* ORF of *Verminephrobacter aporrectodeae* subsp. *tuberculatae* At4. DnfC proteins from *Motilimonas* and *Shewanella* included the N-terminal glutamine amidotransferase and the C-terminal peroxiredoxin domains. Red genus names indicate genera represented by experimentally tested strains.

The *dnfABC* gene clusters of 690 strains could be classified into five groups (I–V) based on the order and orientation of three open reading frames (ORFs; Fig. 2; Fig. S2 to S17, Table S3). Those members sharing the same *dnfABC* order and orientation as those in *A. faecalis* JQ135 were classified into the first group (group I) and indicated by “*dnfABC*,” including members from *Alcaligenes*, *Andreprevotia*, *Bacillus*, *Burkholderia*, *Delftia*, *Herbaspirillum*, *Jeongeupia*, *Microvirgula*, *Pokkaliibacter*, *Providencia*, *Serratia*, *Streptomyces*, *Verminephrobacter*, and *Xenorhabdus*, and some *Pseudomonas* members. The second group (group II) encoded the gene cluster in the order of *dnfC-dnfA-dnfB* (referred to as *dnfCAB*), including members from *Pseudoduganella*, *Stutzerimonas*, and *Xanthobacter*, and 50 strains of *Pseudomonas*. The third group (group III) had the gene cluster in the order of *dnfA-dnfC-dnfB* (referred to as *dnfACB*), including members from *Actinoplanes* and *Paraherbaspirillum*. The fourth group (group IV) had a different *dnfA* ORF orientation from *dnfBC* (referred to as dnfA*BC, * stands for reversed ORF direction), including members from *Motilimonas*, *Shewanella*, *Colwellia*, and *Fluctibacter*. The fifth group (group V) had a different *dnfC* ORF orientation from *dnfAB* (referred to as dnfABC*), including members from *Chromobacterium* and *Xenophilus*. Most strains were classified into group I (607 strains) and II (73) and are limited in the other groups. The DnfA, DnfB, and DnfC in groups I and II (excluding *Streptomyces*) share amino acid sequence identities of 68%–98%, 47%–97%, and 59%–98%, respectively, with those of *Alcaligenes*, much higher than those in the other groups (Fig. 2). These results suggest that gene rearrangements and inversions contributed to the architectural variants of *dnfABC* gene clusters. Since three conserved potential dirammox genera, *Alcaligenes, Delftia*, and *Microvirgula*, shared the same *dnfABC* order and orientation, we hypothesized that the architectural variants of other *dnfABC* gene clusters may have arisen through gene rearrangements, inversions, and/or insertions.

Potential gene cluster regulators were identified in some members in groups I, II, III, and IV, suggesting that the regulatory mechanisms associated with the *dnfABC* gene cluster may differ among these strains. Based on the presence and sequence identity of potential regulators, group I could be further divided into three subgroups: Ia, Ib, and Ic, characterized by DnfR homologs with high sequence identity, low sequence identity, or no detectable DnfR homolog, respectively. The genera with high *dnfABC* prevalence, *Alcaligenes*, *Delftia*, and *Microvirgula*, are classified into group I, indicating that these members may share similar regulatory features associated with *dnfABC*. The *dnfD*, *dnfG*, and *dnfH* genes are frequently observed nearby those *dnfABC* gene clusters, including *dnfGH* in group Ia, and *dnfD* in Groups Ia, Ib, and V. In addition, other neighboring genes were frequently observed across different groups, including *bioA* and *yggS* in group I, *hmoA* in group Ia, *hypO1* in groups Ic, II, and IV, and *mosC* in groups Ic, III, and IV. Most of these neighboring genes remain poorly studied but are thought to be involved in ammonia oxidation or its regulation (Fig. 2). The presence of distinct regulator proteins, gene arrangements, and neighboring genes across groups suggests that different genera may employ varied regulatory mechanisms and strategies associated with the *dnfABC* gene cluster, potentially reflecting different functional roles of the *dnfABC* gene cluster in each group.

### Coupled evolution and lineage-dependent genomic signatures of *dnfABC* loci

**Fig 3:**
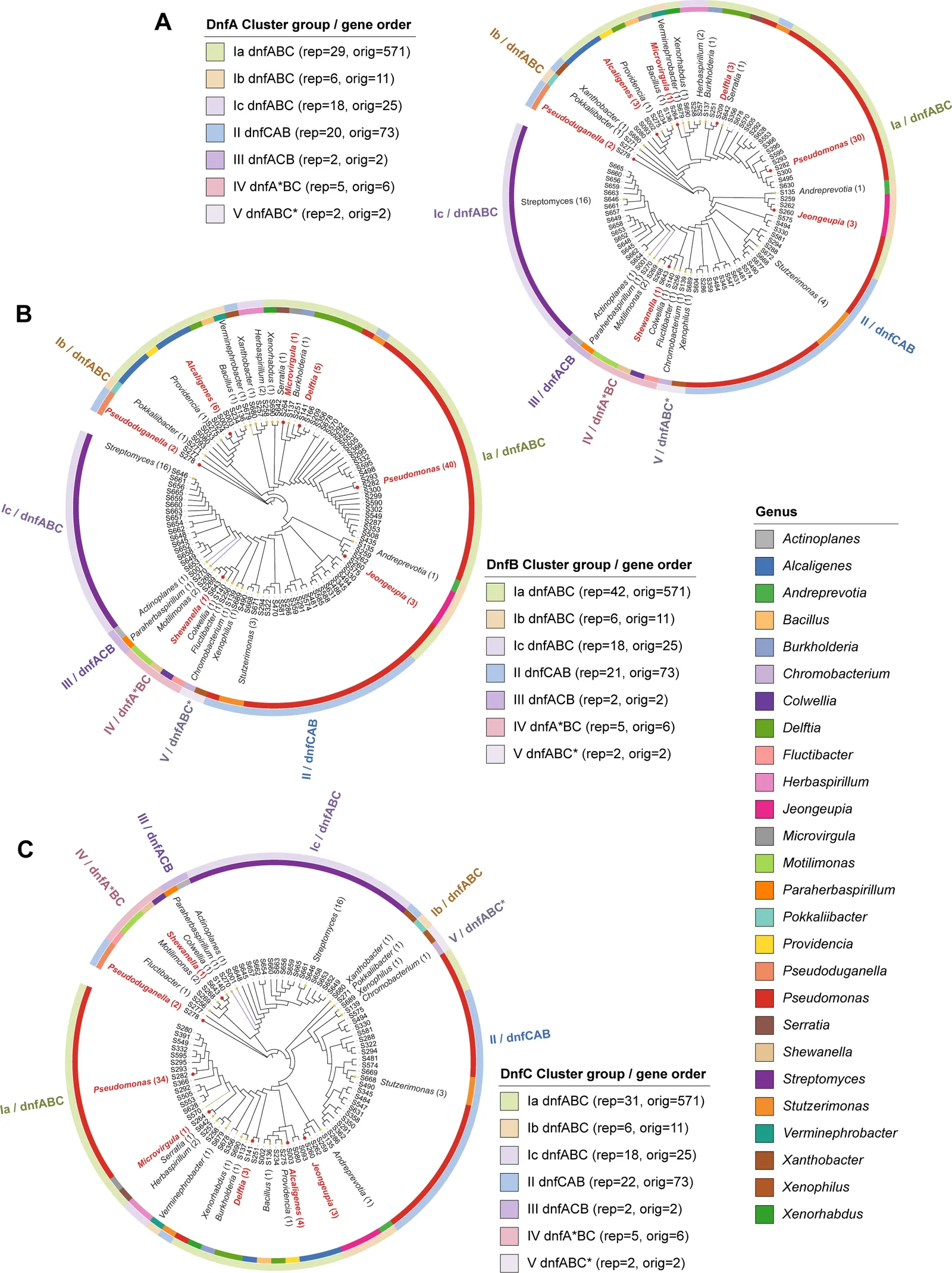
Phylogenetic trees of DnfABC proteins. (**A**) DnfA, (**B**) DnfB, and (**C**) DnfC amino acid sequences were reconstructed using maximum likelihood trees. The outer rings indicate *dnfABC* cluster groups, including I/*dnfABC* (Ia, Ib, and Ic), II/*dnfCAB*, III/*dnfACB*, IV/dnfA*BC, and V/dnfABC*. Different colors on the inner ring represent bacterial genera. Values in parentheses next to genus names indicate the number of representative sequences from each genus. In the cluster group legends, “rep” indicates the number of representative sequences retained after dereplication, whereas “orig” indicates the number of original sequences in the complete sequence set. Red labels indicate experimentally tested strains in representative genera.

To clarify the evolutionary relationships and genomic signatures of the *dnfABC* gene cluster, maximum-likelihood phylogenetic trees were reconstructed using representative amino acid sequences of DnfA, DnfB, and DnfC, and GC content deviation and mobile genetic element (MGE)-associated flanking regions were further examined across 690 *dnfABC*-containing genomes (Fig. 3; Supplemental results; Fig. S18 to S24). Based on the previously defined gene cluster classification, these genomes were assigned to seven cluster categories: Ia/*dnfABC* (571 strains), Ib/*dnfABC* (11), Ic/*dnfABC* (25), II/*dnfCAB* (73), III/*dnfACB* (2), IV/*dnfABC* (6), and V/*dnfABC* (2). After 95% identity dereplication within each cluster category, 82 DnfA, 96 DnfB, and 86 DnfC representative sequences were retained for phylogenetic reconstruction. The DnfA, DnfB, and DnfC phylogenies showed broadly congruent branching patterns, suggesting that the three proteins have diversified in a largely coupled manner. Major branches were associated with both taxonomic affiliation and the *dnfABC* gene cluster organization. The canonical Ia/*dnfABC* group formed the largest and most widely distributed lineage, mainly represented by *Pseudomonas*, *Alcaligenes*, and *Delftia*. In contrast, the Ic and II groups occupied more distinct phylogenetic positions, whereas the rare rearranged groups III, IV, and V formed small but discrete branches (Fig. 3A through C; Fig. S18). Compared with DnfA and DnfC, DnfB exhibited a more dispersed topology, suggesting protein-specific sequence divergence within the cluster’s overall coupled evolution. These patterns suggest that *dnfABC* has diversified as a gene cluster, with structural rearrangements and lineage-specific evolution contributing to its present diversity.

Genomic context analyses further revealed lineage-dependent signatures of *dnfABC* loci. At the cluster level, most complete *dnfABC* loci showed slightly higher GC content than their host genomes, but the overall signed GC deviation was small, whereas the absolute deviation indicated substantial locus-level variability. At the gene level, *dnfA* was predominantly GC-depleted relative to host genomes, whereas *dnfB* and *dnfC* were mainly GC-enriched, indicating that the three genes contribute unequally to the GC composition of the cluster. Genus-level GC deviation varied substantially across taxa, with larger deviations mainly observed in low-abundance genera, including *Burkholderia*, *Xenorhabdus*, *Shewanella*, *Stutzerimonas*, *Verminephrobacter*, and *Xanthobacter*. In contrast, dominant *dnfABC*-harboring genera, such as *Pseudomonas*, *Delftia*, and *Alcaligenes*, showed more moderate deviations (Supplemental Results; Fig. S19 to S22A).

Analysis of 20-CDS flanking regions identified strict MGE-related proteins near 207 of 690 *dnfABC* clusters (30.0%), mainly including integrases, transposases, IS-family transposases, and phage-associated proteins. MGE signals were unevenly distributed among genera, with high frequencies in *Xanthobacter*, *Pseudoduganella*, *Herbaspirillum*, and *Pseudomonas*, but much lower frequencies in *Delftia* and *Alcaligenes*. No strict MGE signals were detected in several genera, including *Microvirgula*, *Shewanella*, *Verminephrobacter*, and *Motilimonas* (Supplemental Results; Fig. S22 to S24). The coupled phylogenetic patterns, GC divergence, and uneven MGE associations suggest that *dnfABC* evolution has been shaped by lineage-specific diversification and, in some bacterial lineages, could be consistent with possible horizontal gene transfer or MGE-associated rearrangement. However, these genomic signatures were not universal across all *dnfABC*-harboring taxa, indicating that both conserved vertical inheritance and lineage-dependent genomic mobility may have contributed to the diversification of this gene cluster.

### The dirammox gene cluster co-occurs with multiple nitrogen cycling pathways, but not nitrification or anammox

**Fig 4:**
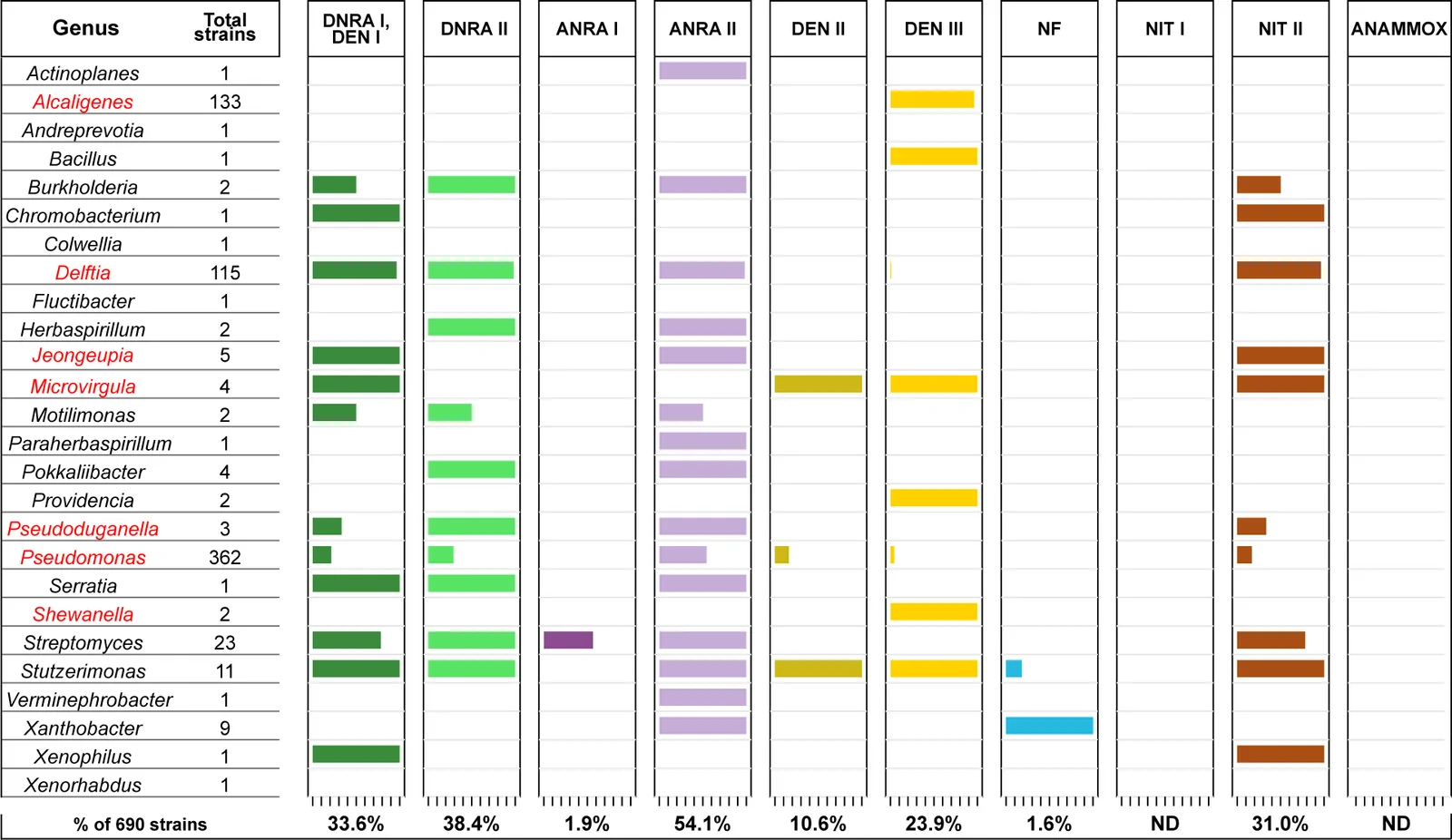
The statistics of different nitrogen metabolism (sub-)pathways in potential dirammox microorganisms across 26 genera. Each (sub-)pathway is represented by a distinct color, and the colored bars indicate the proportion (%) of genomes within each strain harboring the corresponding pathway markers. The 10 pathway categories include DNRA I/DEN I (pathway 1; dissimilatory nitrate reduction to ammonium step I/denitrification step I), DNRA II (pathway 2), ANRA I (pathway 3; assimilatory nitrate reduction to ammonium steps I), ANRA II (pathway 4), DEN II (pathway 5), DEN III (pathway 6), NF (pathway 7; nitrogen fixation), NIT I (pathway 8; nitrification steps I), NIT II (pathway 9), and ANAMMOX (pathway 10). Pathway assignments were based on representative marker genes, and a pathway was considered present only when all required steps were detected. Percentages shown at the bottom indicate the proportion of the 690 *dnfABC*-containing genomes carrying each pathway category. NIT I and ANAMMOX were not detected (ND) in the analyzed genomes. The NIT II category is based on *nxrAB*-like genes, which may reflect homology with *narGH* and therefore does not necessarily indicate nitrite-oxidizing capacity. Red genus names indicate genera represented by experimentally tested strains.

To determine the broader genetic context of nitrogen metabolic backgrounds of potential dirammox microorganisms, we surveyed genes involved in major nitrogen transformation pathways across all 690 genomes. The *dnfABC* clusters co-occurred with dissimilatory nitrate/nitrite reduction to ammonium (DNRA), assimilatory nitrate/nitrite reduction to ammonium (ANRA), denitrification, and/or nitrogen fixation, but complete nitrification and anammox pathways were not detected (Fig. S25A). DNRA was widely distributed among those genomes. For the nitrate reduction to nitrite (NO_3_^−^ → NO_2_^−^) step in DNRA, referred to as DNRA step I and pathway 1 (Fig. S25A), 232 genomes encoded genes for nitrate reduction, including *narGHI, narZYV*, or *napAB*, accounting for 33.6% of the total genomes. For the nitrite reduction to ammonium (NO_2_^−^ → NH_4_^+^) in DNRA, referred to as DNRA step II and pathway 2 (Fig. S25A), 265 genomes encoded nitrite reduction genes, including *nirBD* or *nrfAH*, accounting for 38.4%. It was mainly detected in dominant genera, such as *Delftia* and *Pseudomonas*. Only 13 genomes encoded *narB*, *NR*, or *nasAC* that respond to assimilatory nitrate reduction to nitrite (NO_3_^−^ → NO_2_^−^; ANRA step I or pathway 3), accounting for 1.9% of the total genomes, and all belonged to *Streptomyces*. In contrast, the assimilatory nitrite reduction to ammonium (NO_2_^−^ → NH_4_^+^; ANRA step II, pathway 4) was the most prevalent pathway, with 373 genomes encoding *NIT6*, *nirA*, or *nasBDE*, accounting for 54.1%. This pathway was detected across 15 genera. Denitrification was observed in *Alcaligenes*, *Bacillus*, *Microvirgula*, *Providencia*, *Shewanella*, and *Stutzerimonas*. The first step of denitrification (DEN I) corresponded to pathway 1, which was shared with DNRA step I. In the subsequent denitrification steps (NO_2_^−^ → NO → N_2_O referred to as DEN II and pathway 5, and N_2_O → N_2_ as DEN III and pathway 6), 31.0% of genomes encoded *nirK/nirS* and *norB/norC* (pathway 5), whereas 23.4% encoded *nosZ* (pathway 6). We also observed nitrogen fixation genes (N_2_ → NH_3_, pathway 7) in *Stutzerimonas* and *Xanthobacter*, accounting for 1.6% of those genomes. Genes related to the oxidation of ammonium to nitrite (nitrification step I, pathway 8) and anammox (pathway 10) were not detected. Although *nxrAB*-like genes associated with nitrite oxidation (nitrification step II, pathway 9) were identified, they may reflect homology with *narGH* genes in pathway 1 rather than a capacity for nitrite oxidation (Fig. 4; Fig. S25B; Table S3). These patterns demonstrate that the genetic potential for dirammox may be metabolically compatible with several reductive nitrogen pathways and nitrogen fixation but remains independent of classical ammonia oxidation and anaerobic ammonium oxidation.

**Fig 5:**
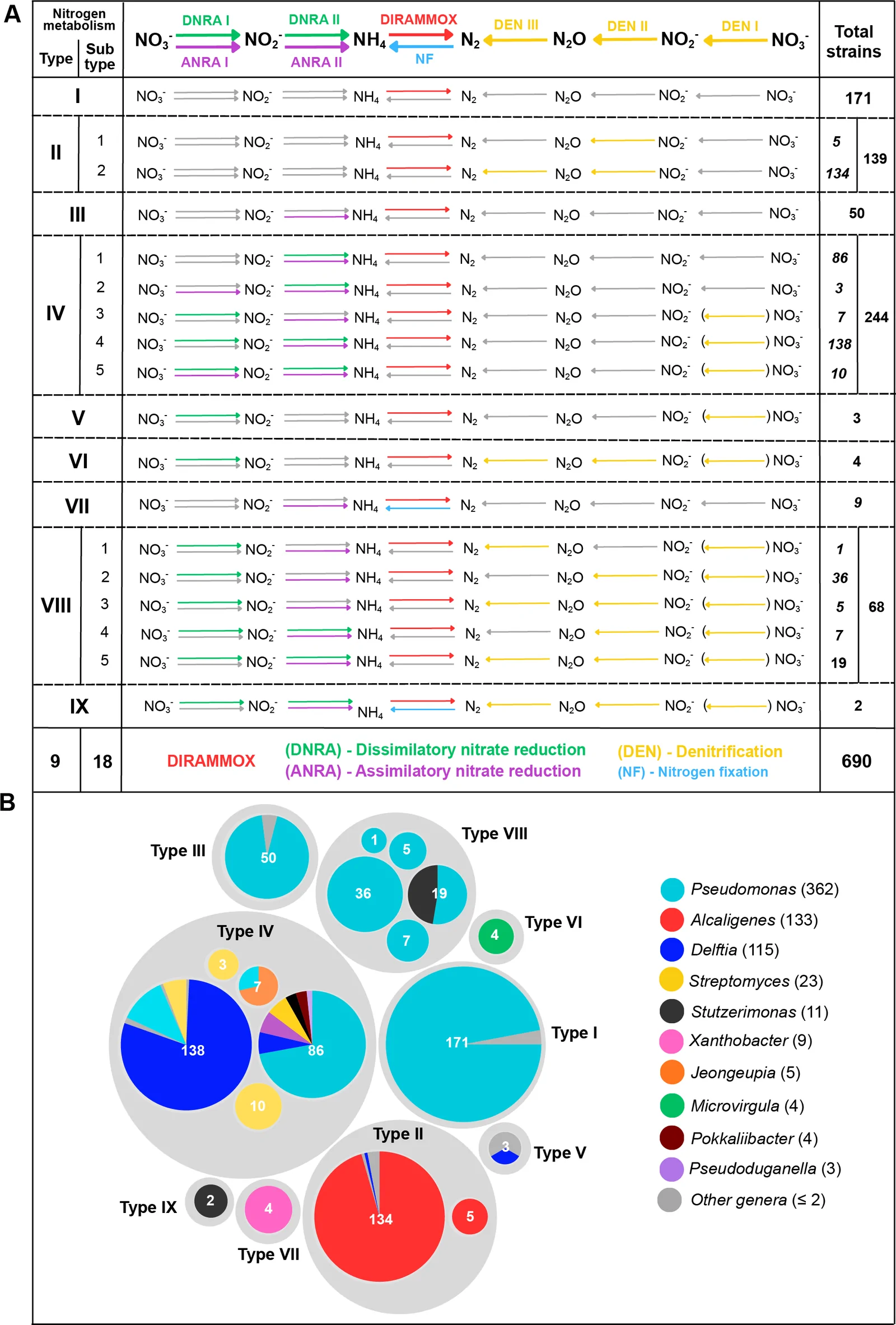
Diverse nitrogen metabolism types in potential dirammox microorganisms. (**A**) Combination types of nitrogen metabolism pathways in 690 *dnfABC*-containing strains were classified into nine major types and 18 subtypes based on the presence of dirammox, DNRA, ANRA, denitrification, and/or nitrogen fixation pathways. Each subtype is defined by distinct pathway combinations, and arrows indicate the corresponding reaction steps within the nitrogen cycle. The arrow shown in parentheses indicates that this pathway may occur concurrently. The numbers in the table denote the total number of strains assigned to each type and subtype. (**B**) Taxonomic distribution of pathway groups across 26 genera. Circle sizes are proportional to the number of strains within each type, and color represents the corresponding genus.

Furthermore, these nitrogen metabolism patterns can be classified into 9 major types and 18 subtypes based on pathway combinations. The nine major types included *dnfABC* only (type I); *dnfABC* combined with denitrification (type II), ANRA (type III), DNRA and ANRA (type IV), DNRA (type V), DNRA and denitrification (type VI), ANRA and nitrogen fixation (type VII), DNRA, ANRA, and denitrification (type VIII), and DNRA, ANRA, denitrification, and nitrogen fixation (type IX; Fig. 5A). Types I, II, and IV were the most abundant among the nine nitrogen metabolism types, comprising 171, 139, and 244 genomes, respectively. Type I was dominated by *Pseudomonas*, type II by *Alcaligenes*, and type IV by *Delftia*. Nitrogen metabolism type IX was observed in two *Stutzerimonas* genomes (Fig. 5A and B; Fig. S26 to S34), representing the most metabolically versatile combination of pathways. This diversity of predicted nitrogen metabolic traits suggests that dirammox might occur together with multiple nitrogen transformation pathways and could have broader ecological relevance than previously recognized.

### The *dnfABC* clusters functioned in *E. coli* but not consistently in the native hosts

**Fig 6:**
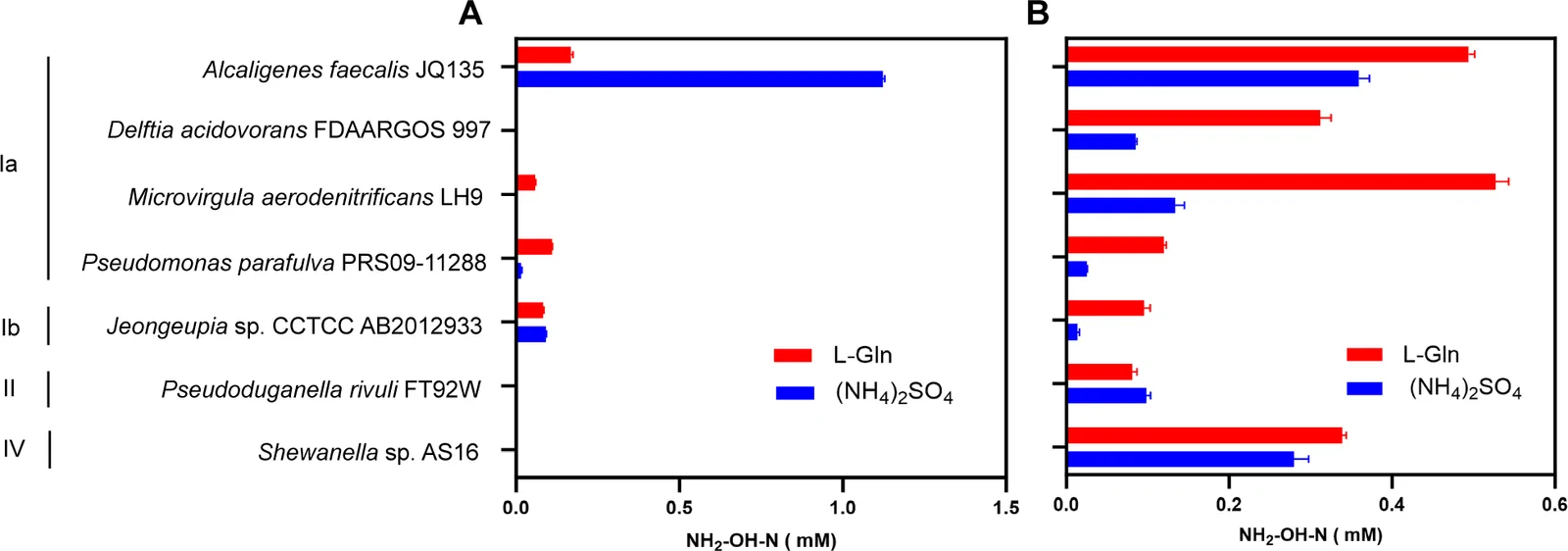
Hydroxylamine accumulation of native strains and *E. coli* harboring *dnfABC* gene cluster using ammonium or L-glutamine as substrate. (**A**) The natural strains harboring *dnfABC* gene cluster, including *A. faecalis* JQ135, *D. acidovorans* FDAARGOS 997, *M. aerodenitrificans* LH9, *P. parafulva* PRS09-11288, *Jeongeupia* sp. CCTCC AB 2,012,933, *P. rivuli* FT92W, and *Shewanella* sp. AS16, were tested for hydroxylamine accumulation at 12 h. (**B**) *E. coli* cells harboring the *dnfABC* gene cluster were tested at 120 min. Blue and red bars represent hydroxylamine accumulation with ammonium and L-glutamine as substrates, respectively. Error bars indicate standard deviations from triplicate measurements.

To assess the functional potential of *dnfABC* gene clusters across different lineages, we evaluated ammonium- and glutamine-oxidizing activities in six representative *dnfABC*-encoding bacterial strains from distinct cluster groups and genera. Among those 690 strains (besides *Alcaligenes*), we collected four strains, i.e., *Delftia acidovorans* FDAARGOS 997 (=CGMCC 1.3363; group Ia), *Pseudomonas parafulva* PRS09-11288 (=CGMCC 1.15632; group Ia), *Pseudoduganella rivuli* FT92W (group II), and *Shewanella* sp. AS16 (group IV). In addition, we obtained two additional strains, *Microvirgula aerodenitrificans* LH9 (group Ia) and *Jeongeupia* sp. CCTCC AB (group Ib), which were not from those 690 strains, yet were confirmed to encode *dnfABC* by cloning. Those six strains were tested for hydroxylamine accumulation using ammonium or L-glutamine as the sole nitrogen source. Among the tested strains, *P. parafulva* PRS09-11288 and *Jeongeupia* sp. CCTCC AB 2012933 were capable of oxidizing both ammonium and L-glutamine to hydroxylamine. *M. aerodenitrificans* LH9 oxidized only L-glutamine. The other strains were not observed to produce hydroxylamine (Fig. 6A; Fig. S35). In addition, none of the other tested *Delftia* strains accumulated hydroxylamine, and some tested *Alcaligenes* strains also showed no detectable hydroxylamine accumulation (Fig. S36).

To check the ammonium and glutamine oxidation potential of *dnfABC* gene clusters from the seven strains, we assayed the engineered *E. coli* strains harboring each *dnfABC* gene cluster via whole-cell transformation assays. All seven *dnfABC* clusters enabled *E. coli* to accumulate hydroxylamine when either L-glutamine or ammonium was supplied as the substrate, although the amounts varied among the clusters and between the two substrates (Fig. 6B). These results demonstrate that all the tested *dnfABC* gene clusters enable *E. coli* to oxidize both ammonium and glutamine to hydroxylamine. Notably, the *dnfABC* cluster from *Shewanella* sp. AS16, which belongs to group IV, shared only 37%, 26%, and 48% sequence identity with the corresponding DnfA, DnfB, and DnfC proteins from *Alcaligenes*, respectively. This finding suggests that *dnfABC* clusters with relatively low amino acid sequence similarity can still retain functional activity. Although only representative gene clusters from groups Ia, Ib, II, and IV were examined, these results suggest that *dnfABC* gene clusters from diverse genera retain the functional potential to catalyze the oxidation of both ammonium and glutamine. However, the variable hydroxylamine accumulation observed among native strains indicates that the *dnfABC* gene cluster may be tightly regulated in native hosts. This regulation may involve regulatory proteins located near the *dnfABC* gene cluster.

## DISCUSSION

Dirammox, the ammonia oxidation pathway reported after nitrification and anammox, is a recently discovered nitrogen transformation pathway in heterotrophic nitrifiers such as *A. ammonioxydans* HO-1 and *A. faecalis* JQ135 (13–15). It provides a previously unrecognized oxidative route to the nitrogen cycle. By integrating large-scale genomic, phylogenetic, and physiological analyses, our study demonstrates that potential dirammox microorganisms are far more taxonomically widespread, genetically diverse, and metabolically versatile than previously appreciated. This diversity spans taxonomy, gene cluster architecture, protein sequence variation, nitrogen metabolic context, and heterotrophic nitrification activity, collectively indicating substantial diversity in the genetic potential for nitrogen transformation among potential dirammox microorganisms.

We found complete *dnfABC* gene clusters in 26 genera, comparable to those of ammonia-oxidizing bacteria (AOB), ammonia-oxidizing archaea (AOA), and comammox. Although *dnfABC*-encoding microorganisms are taxonomically diverse, they occur predominantly within the Proteobacteria, and the gene cluster is nearly ubiquitous only in *Alcaligenes*, *Delftia*, and *Microvirgula*. In contrast, ammonia-oxidizing microorganism (AOM)-associated genes (*amoABC*, *hao*) are broadly conserved across ammonia-oxidizing genera, reflecting their essential role in chemolithoautotrophic energy metabolism (21). The broad habitat distribution of *dnfABC*-containing microorganisms, encompassing soils, freshwater, marine systems, activated sludge, and plant- and animal-associated environments, suggests that they are well adapted to organic nitrogen-rich, heterotrophic niches. This observation is consistent with the metabolic properties of model dirammox organisms such as *A. faecalis* JQ135, which can oxidize both ammonium and organic nitrogen substrates (16). In contrast, canonical ammonia-oxidizing microorganisms are primarily associated with soil and aquatic environments (22–25). This distinction suggests that potential dirammox microorganisms may contribute to nitrogen cycling across a broader range of habitats than previously recognized. Moreover, the ability of dirammox to directly convert ammonium to N_2_ independently of other nitrogen transformation pathways suggests that it may expand the classical framework of heterotrophic nitrification–aerobic denitrification.

Our analysis reveals that genetic potential for dirammox may occur alone or in combination with genes associated with ANRA, DNRA, denitrification, and nitrogen fixation. These different combinations of dirammox with other nitrogen metabolic pathways highlight the diverse nitrogen metabolic potential of potential dirammox microorganisms. Importantly, the *dnfABC* gene cluster did not co-occur with marker genes for nitrification or anammox genes, reinforcing the notion that this heterotrophic oxidative pathway occupies a distinct ecological niche from canonical ammonia oxidizers. For instance, a single cell with the genetic potential for DNRA or ANRA but lacking denitrification genes could reduce nitrate to ammonium and subsequently oxidize ammonium to nitrogen gas through dirammox, thereby enabling a complete route for nitrogen loss in the absence of denitrification. However, these patterns should be interpreted as genomic associations rather than evidence of pathway exclusivity, causality, or realized activity, because the analysis was not normalized by phylogenetic background or genome completeness.

The bimodal distribution of *dnfABC* is further supported by phylogenetic and genomic evidence. Group I lineages showed conserved gene architecture and coherent DnfA/DnfB/DnfC phylogenetic patterns, suggesting long-term retention and possible vertical inheritance in core genera. In contrast, the sporadic *dnfABC* occurrence in diverse Proteobacteria and Actinobacteria was associated with more variable cluster organizations, lineage-dependent GC deviation, and flanking MGE-related genes, suggesting that horizontal gene transfer or MGE-mediated genomic rearrangement may have contributed to its distribution in some lineages. However, these signatures were unevenly distributed across taxa, indicating that horizontal transfer alone cannot fully account for the observed distribution of *dnfABC*. Thus, the bimodal pattern likely reflects the combined effects of ecological compatibility, metabolic trade-offs, vertical inheritance, and lineage**-**dependent horizontal transfer. Functional assays revealed a mismatch between the catalytic potential encoded by *dnfABC* clusters and their activity in native hosts: although all tested clusters conferred ammonium- and glutamine-oxidizing activity in *E. coli*, only some native host strains produced hydroxylamine. These results suggest that *dnfABC*-associated activity is tightly regulated in a condition-dependent manner. The mechanisms underlying this regulation remain unclear and may involve environmental, transcriptional, post-translational, or metabolic controls. This contrast is clearly illustrated by *Alcaligenes* and *Delftia*, whose *dnfABC* clusters share moderate sequence identity and are functionally active in *E. coli*, yet only *Alcaligenes* showed activity under the tested conditions. The differing genomic contexts, with *Delftia* lacking *dnfD* and *dnfH* but harboring *hmoA*, *yggS*, and *bioA*, suggest that pathway expression depends on regulatory and accessory-gene environments rather than protein function alone. Because dirammox is heterotrophic, oxygen dependent, and energy neutral and produces reactive hydroxylamine, its expression likely requires tight coordination with carbon metabolism and redox homeostasis. These constraints may restrict dirammox activity to carbon-rich, nitrate-poor, or fluctuating-nitrogen habitats where classical nitrification is suppressed.

Our results show that the potential dirammox microorganisms complement rather than compete with AOB, AOA, and comammox, offering a direct heterotrophic route for converting ammonium and organic nitrogen to N_2_ in settings where classical nitrification–denitrification may be ineffective. By operating independently of nitrite and nitrate, dirammox broadens the conditions under which aerobic N_2_ production can occur and reveals overlooked nitrogen-loss pathways in organic-rich environments. However, our conclusions are based largely on genomic potential and laboratory assays rather than direct measurements of *in situ* activity. Therefore, the ecological contribution of dirammox remains to be quantified in natural environments. Given the widespread yet tightly regulated nature of *dnfABC*, resolving when and where the *dnfABC* gene cluster is expressed and quantifying its contribution to ecosystem N_2_ fluxes should be priorities for future work. Such efforts will clarify how this previously underappreciated pathway fits into the broader nitrogen cycle and interacts with canonical ammonia oxidizers across diverse habitats.

These findings suggest that the widespread distribution of *dnfABC*, its potential substrate flexibility, and its association with diverse nitrogen metabolic pathways may allow potential dirammox microorganisms to contribute more broadly to nitrogen transformation than previously recognized. Given that the aerobic conversion of ammonium and organic nitrogen to N_2_ has largely been overlooked in previous ecological studies, comprehensive ecological investigations are needed to quantify the contribution of dirammox to nitrogen cycling. Such efforts will advance our understanding of the ecological role of dirammox and may reveal previously unrecognized pathways influencing nitrogen fluxes in natural and artificial ecosystems.

## MATERIALS AND METHODS

### Distribution analysis of the *dnfABC* gene cluster in the public database

To assess the taxonomic distribution of the *dnfABC* gene cluster, publicly available RefSeq and GenBank assemblies of Archaea, Bacteria, and Fungi released by 23 May 2026 were downloaded from NCBI. Metagenome-assembled genomes were excluded, and paired GenBank and RefSeq records were dereplicated, resulting in a local database of 3,142,604 nonredundant genomes, including 3,078,488 bacterial, 39,273 archaeal, and 24,843 fungal assemblies. A DnfA profile HMM constructed from 1,001 curated homologs was used to screen the genome database with HMMER v3.4 (26–28). Candidate DnfA-positive genomes were further validated by BLASTP searches for DnfA, DnfB, and DnfC, followed by the extraction of DnfA-centered genomic neighborhoods. Genomes were considered *dnfABC* positive only when DnfA, DnfB, and DnfC co-occurred within the same genomic neighborhood. This pipeline identified 690 nonredundant *dnfABC*-containing genomes. Genome completeness and contamination were assessed using CheckM v1.2.5 with the lineage-specific workflow and the reduced-tree option (29). Estimated completeness ranged from 89.10% to 100.00%. Among the 690 genomes, 689 (99.9%) had an estimated completeness of ≥90%, including 683 (99.0%) with an estimated completeness of ≥95%; 681 genomes had estimated contamination ≤5%. Genus-level prevalence was calculated as the percentage (%) of *dnfABC*-positive genomes among all nonredundant, non-MAG genomes assigned to each genus. Isolation metadata were obtained from NCBI BioSample, and BioProject records verified isolate origins (20).

### Gene cluster organization and phylogenetic analysis

Comparative genomic analyses were performed to investigate the organization of the gene clusters and the orientation of ORFs across all genome sequences retrieved from the NCBI database. Gene cluster groups were classified by comparing differences in gene organization and ORF orientation among different bacterial genera. The arrangements of the gene clusters were visualized using ChiPlot software (https://www.chiplot.online/gene_cluster.html) (30). For phylogenetic analysis, DnfA, DnfB, and DnfC amino acid sequences were dereplicated at 95% identity using CD-HIT within each predefined cluster group and genus stratum to reduce redundancy while retaining cluster- and genus-level representation (Supplemental Methods). Representative sequences were aligned using MAFFT, and maximum-likelihood trees were reconstructed using IQ-TREE2 (31, 32). The resulting trees were visualized in iTOL (https://itol.embl.de/), and final illustrations with key annotations were prepared using Adobe Illustrator 2023 (Adobe Inc., San Jose, CA, USA).

### GC content and flanking MGE analysis

GC content and MGE-associated genomic context were analyzed for the 690 dnfABC-positive genomes using the corresponding genome and annotation files. Whole-genome GC content was calculated for each genome, and GC content was determined for individual *dnfA*, *dnfB*, and *dnfC* genes, concatenated *dnfABC* CDSs, and the complete *dnfABC* locus, including intergenic regions. GC deviation was defined as the difference between the GC content of each target sequence and the corresponding whole-genome GC content (33). To assess MGE-associated genomic context, coding sequences located within 20 genes upstream and downstream of each *dnfABC* locus were screened for strict MGE-related annotations, including transposase-, integrase-, and phage-associated proteins. Cluster- and genus-level MGE frequencies were calculated as the proportion of *dnfABC* loci containing at least one strict MGE-related protein within the flanking region (34). Detailed MGE annotation criteria are provided in Supplemental Methods.

### Nitrogen metabolism pathway analysis

Genomes encoding *dnfABC* were screened for six major nitrogen-cycling pathways using Kyoto Encyclopedia of Genes and Genomes (KEGG) orthology-defined marker genes: assimilatory nitrate reduction (*narB*, *NR*, *nasC–nasA*, and *NIT6/nirA/nasBD–nasE*), dissimilatory nitrate reduction (*narGHI*, *narZYV*, *napAB*, *nirBD*, and *nrfAH*), denitrification (*narGHI*, *narZYV*, *napAB*, *nirK/nirS*, *norB–norC*, and *nosZ*), nitrogen fixation (*nifHDK*), nitrification (*pmoABC*, *amoABC*, *hao*, *nxrAB*-like genes, and *narZY*), and anammox (*hzsABC* and *hdh*). Protein sequences were screened against the corresponding KOfam HMM profiles, and hits were retained only when their scores met or exceeded the KO-specific thresholds defined by KOfam (35, 36). For each genome and KO, the highest-scoring threshold-passing hit was retained and treated as a putative homolog. A pathway was scored as present only when all required marker genes or pathway steps were detected; genomes containing only partial marker sets were not considered to encode a complete pathway. The detected pathways were further grouped into 10 functional categories based on catalytic roles (Fig. S22A), and each genome was assigned a nitrogen transformation profile based on the combination of identified complete pathway categories.

### Bacterial cultivation, cloning, functional assays, and statistical analysis

All bacterial strains used in this study are listed in Table S1. Strains were cultivated aerobically at 30°C and 160 rpm in LB, R2A, HNM, or modified HNM medium, depending on the experiment (14, 15, 37). For wild-type functional assays, overnight cultures were inoculated into HNM or modified HNM medium, and growth and hydroxylamine accumulation were monitored using colorimetric assays. For heterologous expression, selected *dnfABC* gene clusters were amplified from genomic DNA and cloned into pBAD33 using Gibson Assembly. Recombinant plasmids were introduced into *Escherichia coli* BW25113, and verified transformants were induced with L-arabinose for functional assays. Hydroxylamine accumulation was determined after 120 min as previously described (14, 15, 37, 38). Primer and plasmid information is provided in Table S2, and detailed cultivation, cloning, and assay procedures are described in Supplemental Methods. All experiments were performed in triplicate, and results are presented as mean ± SD. Statistical analyses and data visualization were performed using GraphPad Prism version 10 (GraphPad Software, San Diego, CA, USA).

## Data Availability

Genome assemblies analyzed in this study were primarily retrieved from the NCBI Assembly database (GenBank and RefSeq; https://www.ncbi.nlm.nih.gov/datasets/genome/), with additional sequences obtained from the NCBI Nucleotide database (https://www.ncbi.nlm.nih.gov/nuccore/). Taxonomic classification of all strains was conducted exclusively using the NCBI Taxonomy database (https://www.ncbi.nlm.nih.gov/taxonomy/). Taxonomic lineage information was accessed through the NCBI Taxonomy Browser and the NCBI Datasets taxonomy interface (https://www.ncbi.nlm.nih.gov/datasets/taxonomy/tree/). Environmental and sample-associated metadata were obtained from the NCBI BioSample database (https://www.ncbi.nlm.nih.gov/biosample/). Accession numbers for all analyzed genome assemblies (GCA/GCF) and their corresponding BioSample records (SAMN/SAMEA/SAMD) are provided in Table S3. All referenced data are publicly available and accessible without restriction.

## SUPPLEMENTAL MATERIAL

Supplemental figures mbio.01645-26-s0001.pdf Fig. S1 to S24.

Supplemental text mbio.01645-26-s0002.docx Supplemental results and methods.

Supplemental material mbio.01645-26-s0003.pdf Fig. S25 to S36; Tables S1 and S2.

Supplemental table mbio.01645-26-s0004.xlsx Table S3.

ASM does not own the copyrights to Supplemental Material that may be linked to, or accessed through, an article. The authors have granted ASM a non-exclusive, world-wide license to publish the Supplemental Material files. Please contact the corresponding author directly for reuse.
