# Supplementary material for: Diversity of dirammox microorganisms
Source: mBio. 2026 Oct 15;17(11):e01645-26. doi: 10.1128/mbio.01645-26 (PMC13753816; doi:10.1128/mbio.01645-26)
Supplement: Supplementary file 1 [file mbio.01645-26-s0001.pdf]

Diversity of Dirammox Microorganisms

Gantuya Khasbaatar[1,2#], Qun Han[1,2#], Xiao-Kang Wang[1,2#], Lei Song[3], Chang Liu[1,2], Xin Sun[1,2], Ze-Shen Liu[1], Ying Liu[1], Cheng-Ying Jiang[1], Ji-Guo Qiu[4], Shuang-Jiang Liu[1], Yanning Zheng[1,2*], De-Feng Li[1,2*]

[1] State Key Laboratory of Microbial Diversity and Innovative Utilization, Institute of Microbiology, Chinese Academy of Sciences, Beijing 100101, China

[2] College of Life Sciences, University of Chinese Academy of Sciences, Beijing 100049, China

[3] China General Microbiological Culture Collection Center, Institute of Microbiology, Chinese Academy of Sciences, Beijing 100101, China

[4] Key Laboratory of Agricultural and Environmental Microbiology, Ministry of Agriculture and Rural Affairs, College of Life Sciences, Nanjing Agricultural University, Nanjing 210095, China

[#] Those authors contribute equally.

***Correspondences:**

De-Feng Li: lidefeng@im.ac.cn, Tel: +86-10-64807015.

Yanning Zheng: zhengyn@im.ac.cn, Tel: +86-10-64806943

Running title: Diversity of Dirammox Microorganisms

This Supplementary Information file includes: Figure S1-S24

Table of Contents

Fig. S1. Environmental distribution of *dnfABC* gene cluster……………………..……………3

Fig. S2 – S17 Comparative organizations of the *dnfABC* gene cluster in genomes of 690 bacterial strains……………………………………………………..……………….……….4-19

Fig. S18. Phylogenetic trees of representative DnfABC proteins……………………….……20

Fig. S19. GC deviation of *dnfABC* genes and loci across genera……………………………….21

Fig. S20. Mean absolute GC deviation of complete *dnfABC* loci across genera……………..…22

Fig. S21. Cluster-level variation in GC deviation of complete *dnfABC* loci…………………....23

Fig. S22. GC deviation and flanking MGE signals of *dnfABC* loci……………………….…24

Fig. S23. Strict MGE-related protein categories near *dnfABC* loci……………………………...25

Fig. S24. Position of strict MGE signals around *dnfABC* loci…………………………………...26

Figure S1

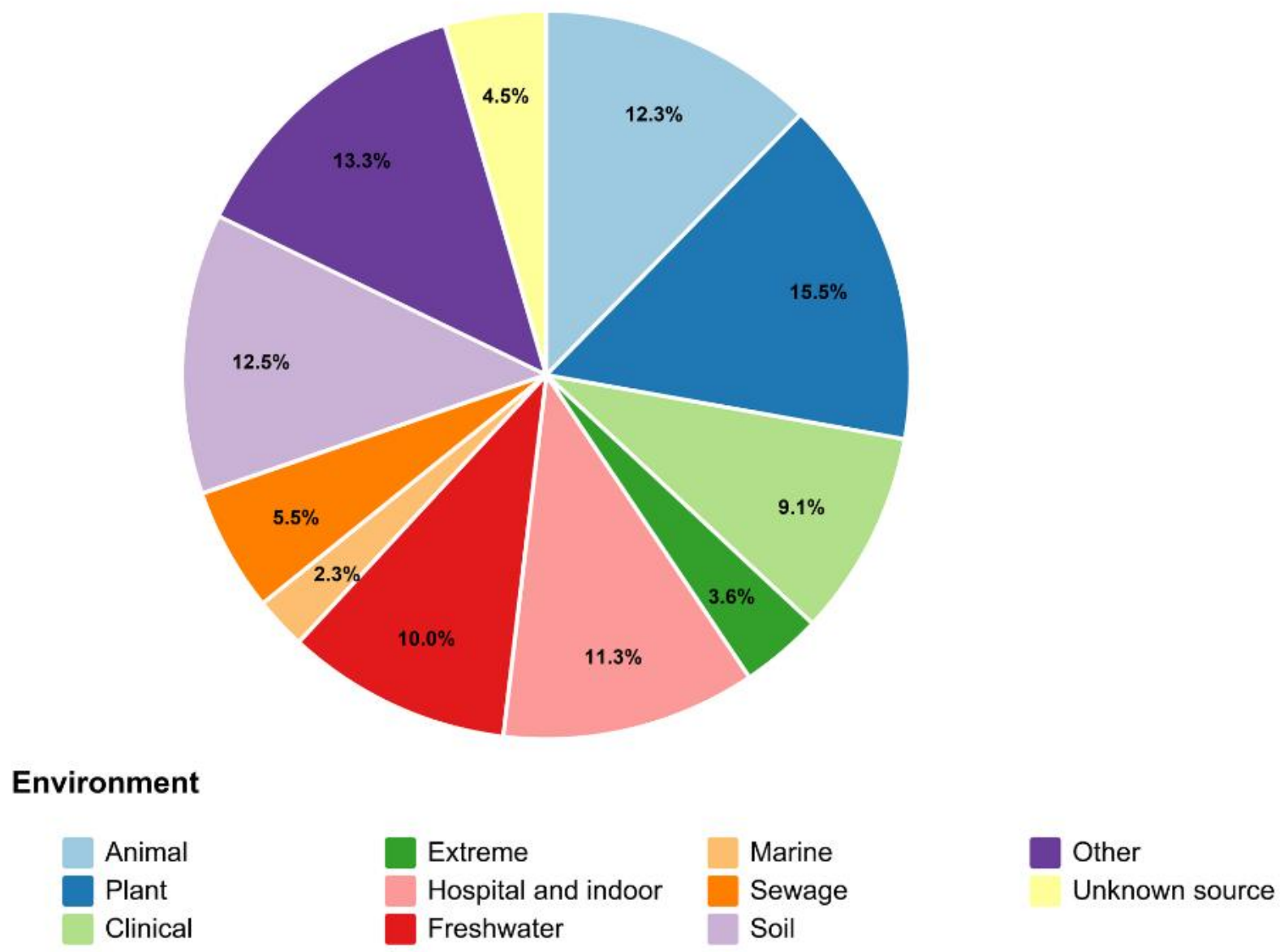

Fig. S1. Environmental distribution of *dnfABC* gene cluster. Environmental distribution of 690 strains harboring the *dnfABC* gene cluster. The pie chart depicts the relative abundance of *dnfABC*-harboring strains across different environments. Colors denote distinct environmental sources, and percentages indicate the proportion of total isolates from each habitat.

Figure S2

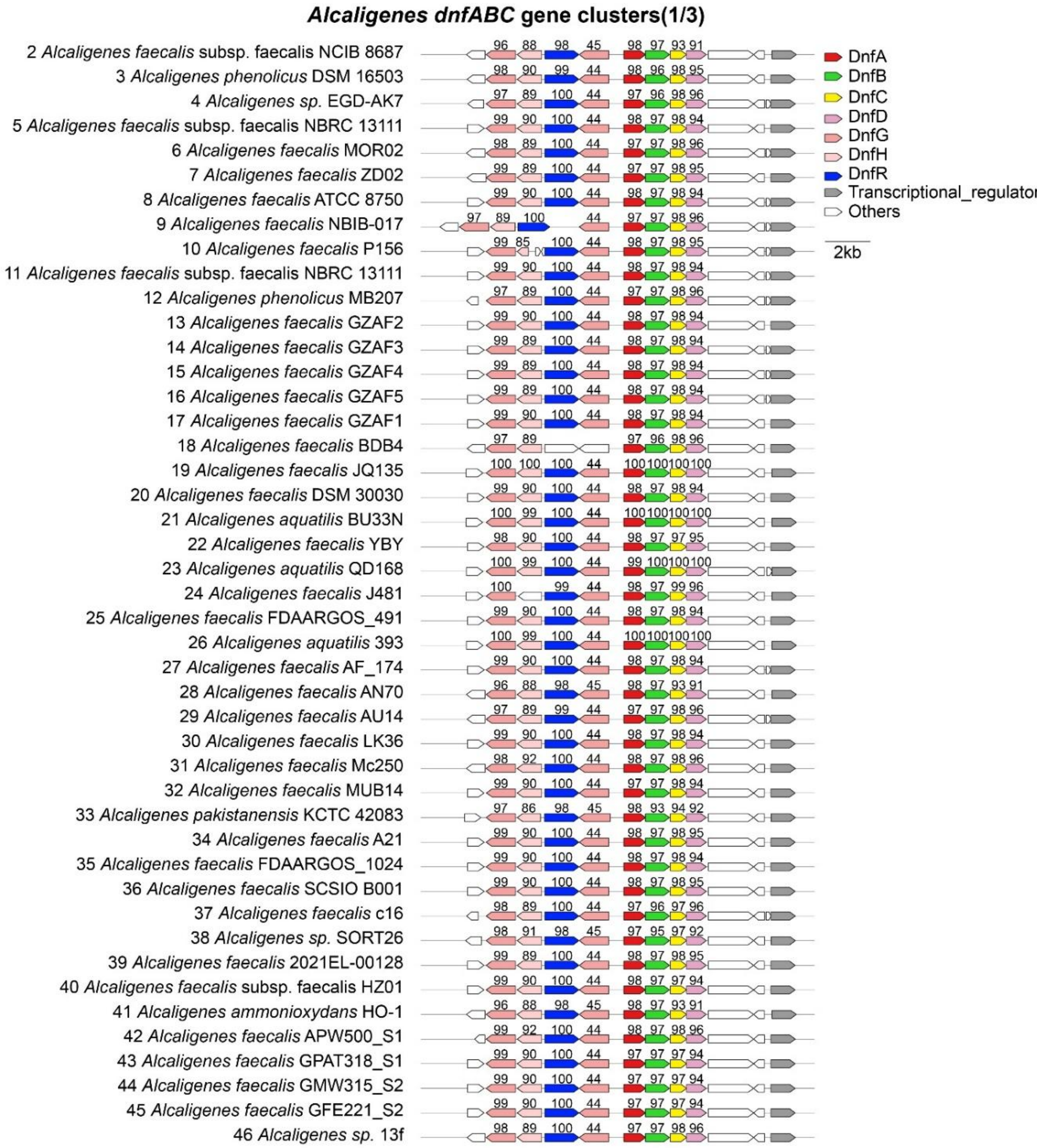

Fig. S2. Comparative organizations of the *dnfABC* gene cluster in genomes of 690 bacterial strains. Colored arrows indicate different genes or functional elements: DnfA (red), DnfB (green), DnfC (yellow), DnfD (pink), DnfR (dark blue), MarR (light blue), and others (grey, pink, yellow). Sequence identities (%) of DnfABC proteins relative to *A. faecalis* JQ135 are shown above each ORF. A 2-kb scale bar represents the relative size of the genomic regions.

Figure S3

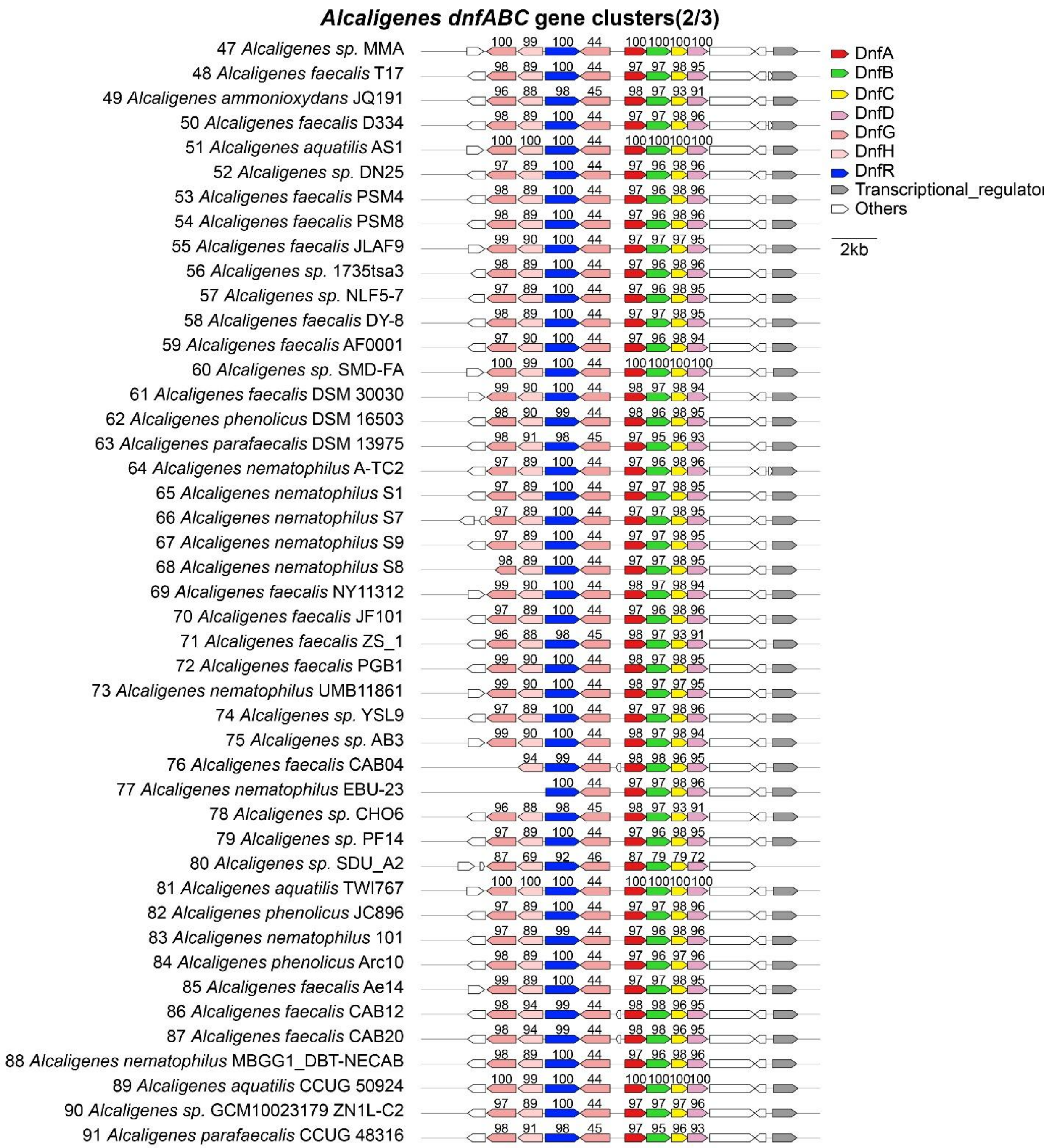

Fig. S3. Comparative organizations of the *dnfABC* gene cluster in genomes of 690 bacterial strains.

Figure S4

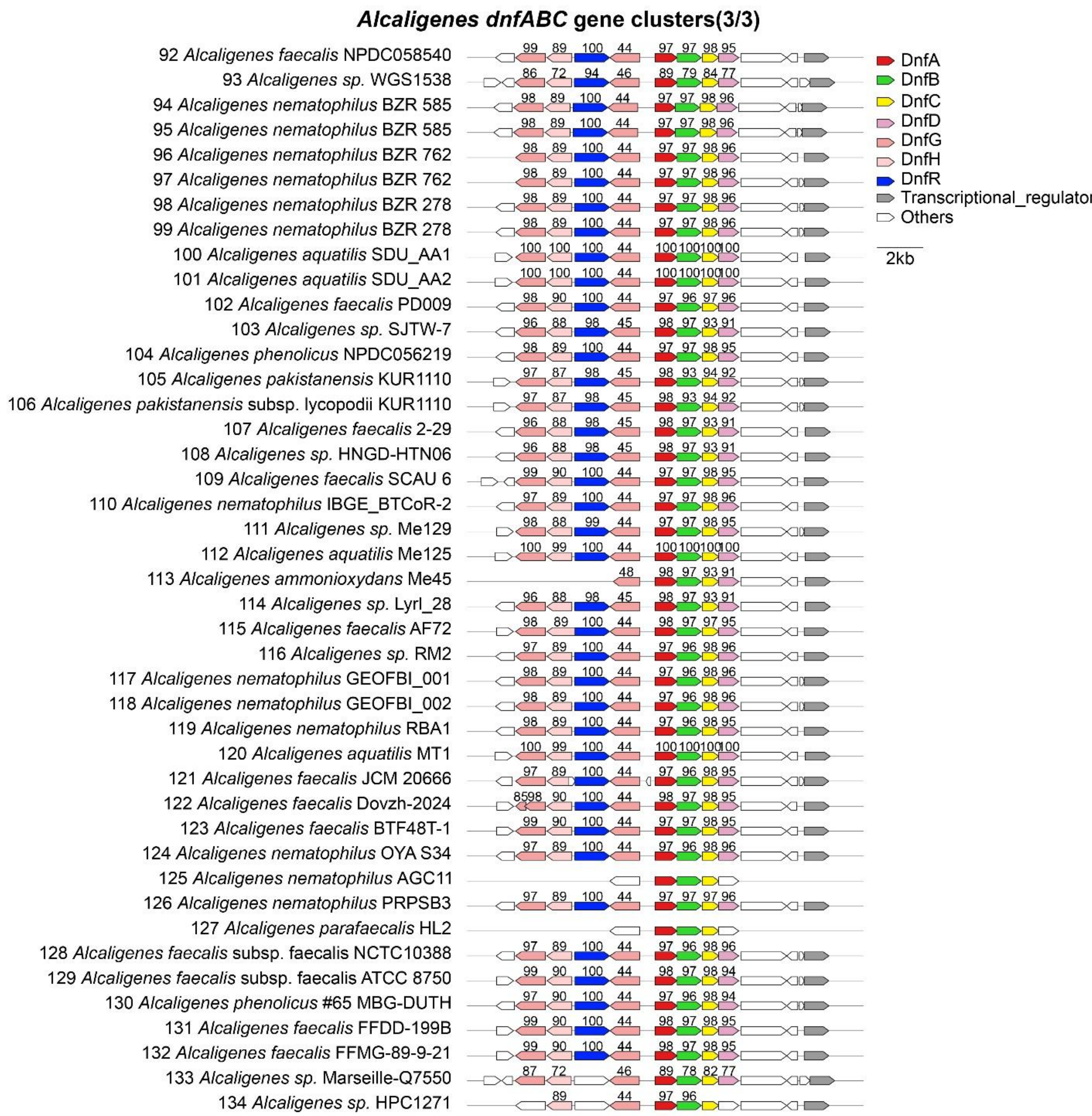

Fig. S4. Comparative organizations of the *dnfABC* gene cluster in genomes of 690 bacterial strains.

Figure S5

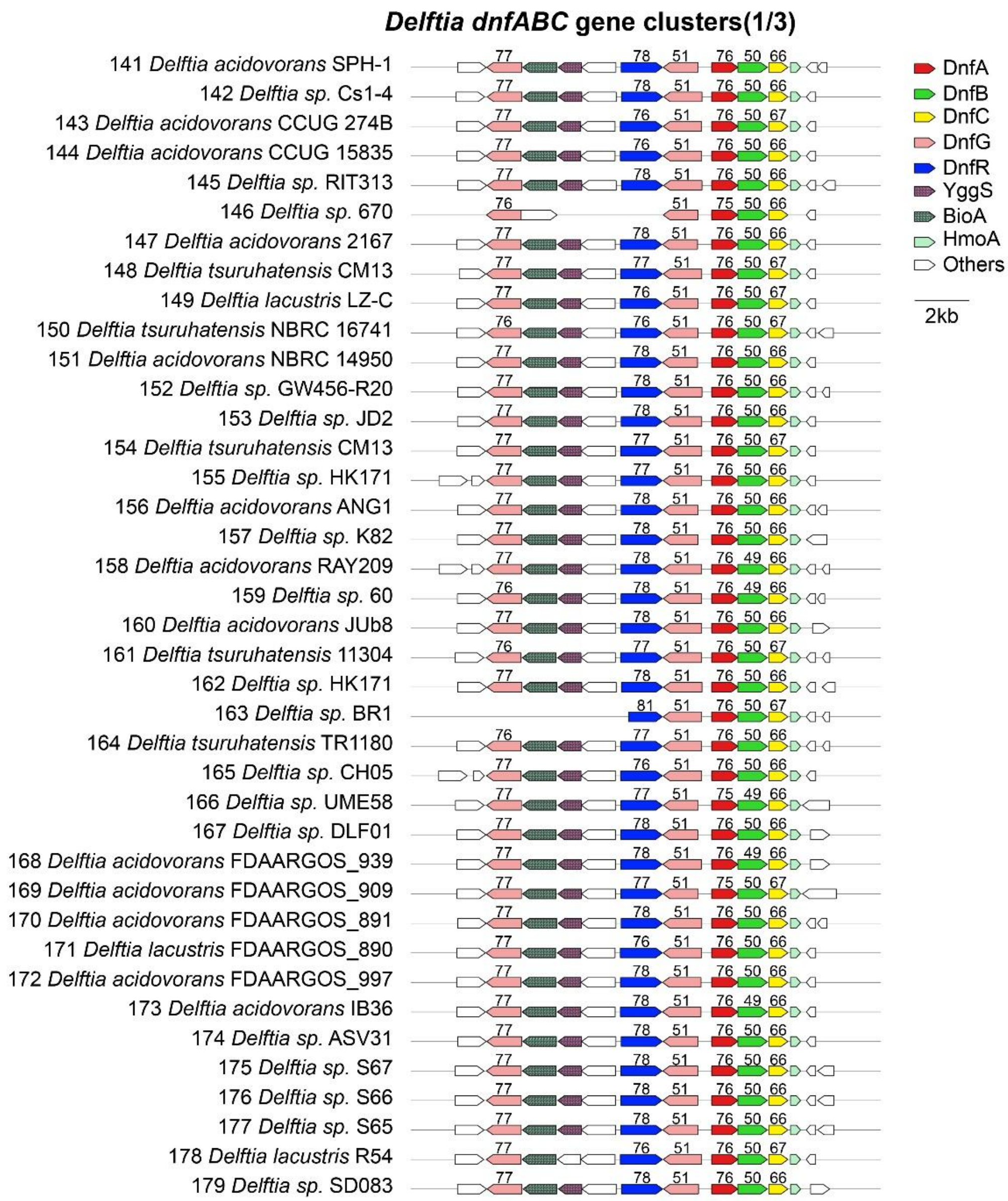

Fig. S5. Comparative organizations of the *dnfABC* gene cluster in genomes of 690 bacterial strains**.**

Figure S6

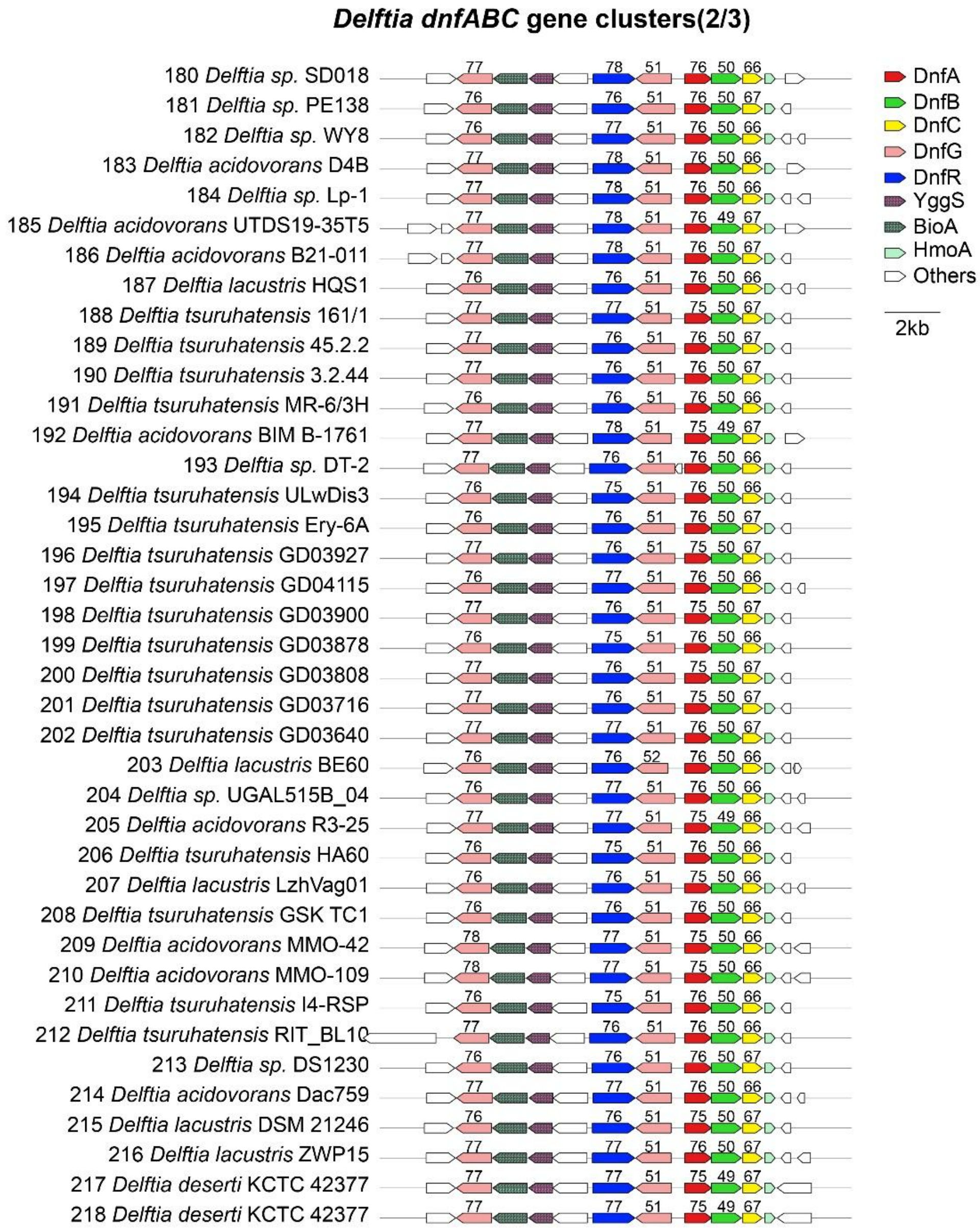

Fig. S6. Comparative organizations of the *dnfABC* gene cluster in genomes of 690 bacterial strains.

Figure S7

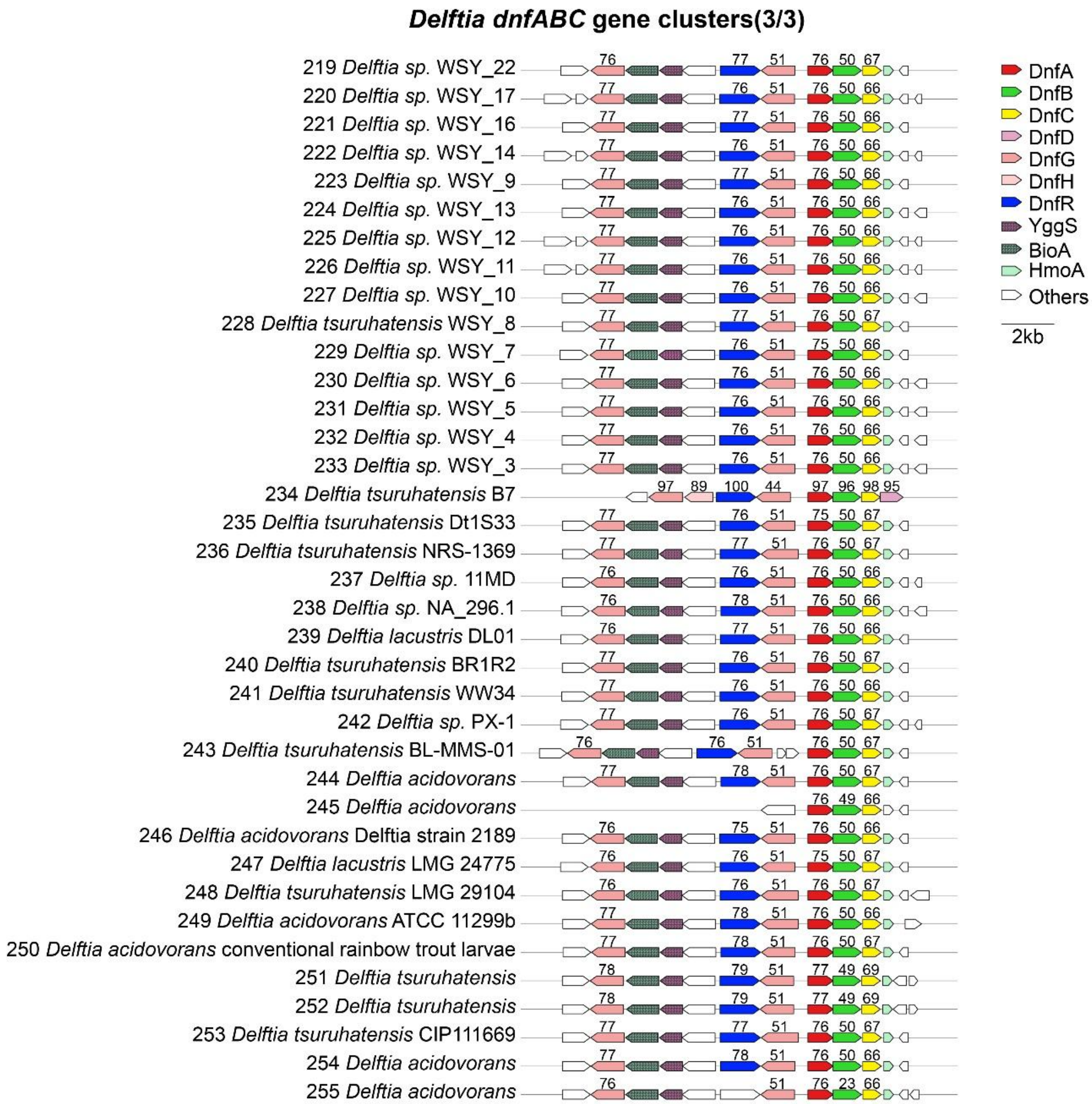

Fig. S7. Comparative organizations of the *dnfABC* gene cluster in genomes of 690 bacterial strains.

Figure S8

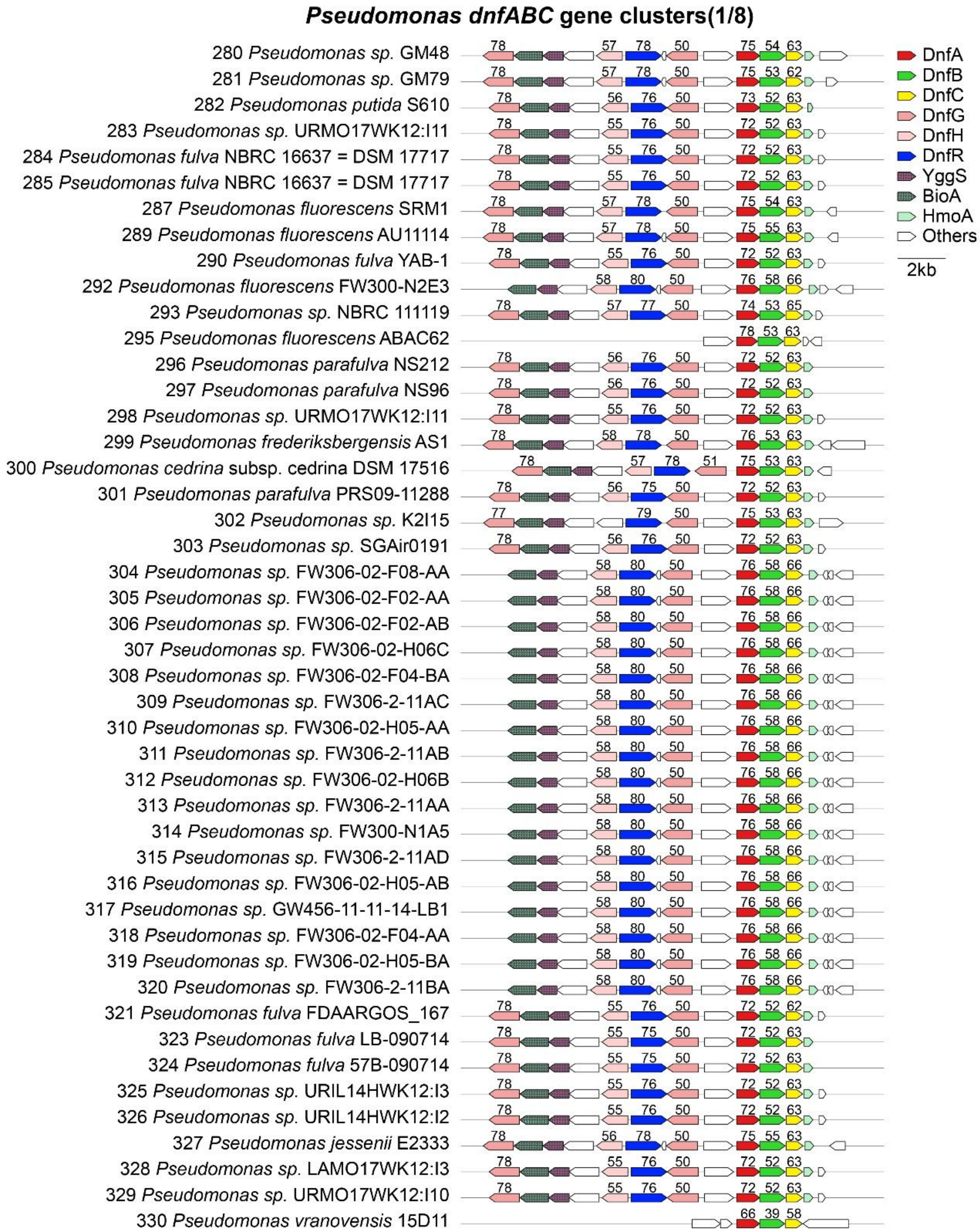

Fig. S8. Comparative organizations of the *dnfABC* gene cluster in genomes of 690 bacterial strains.

Figure S9

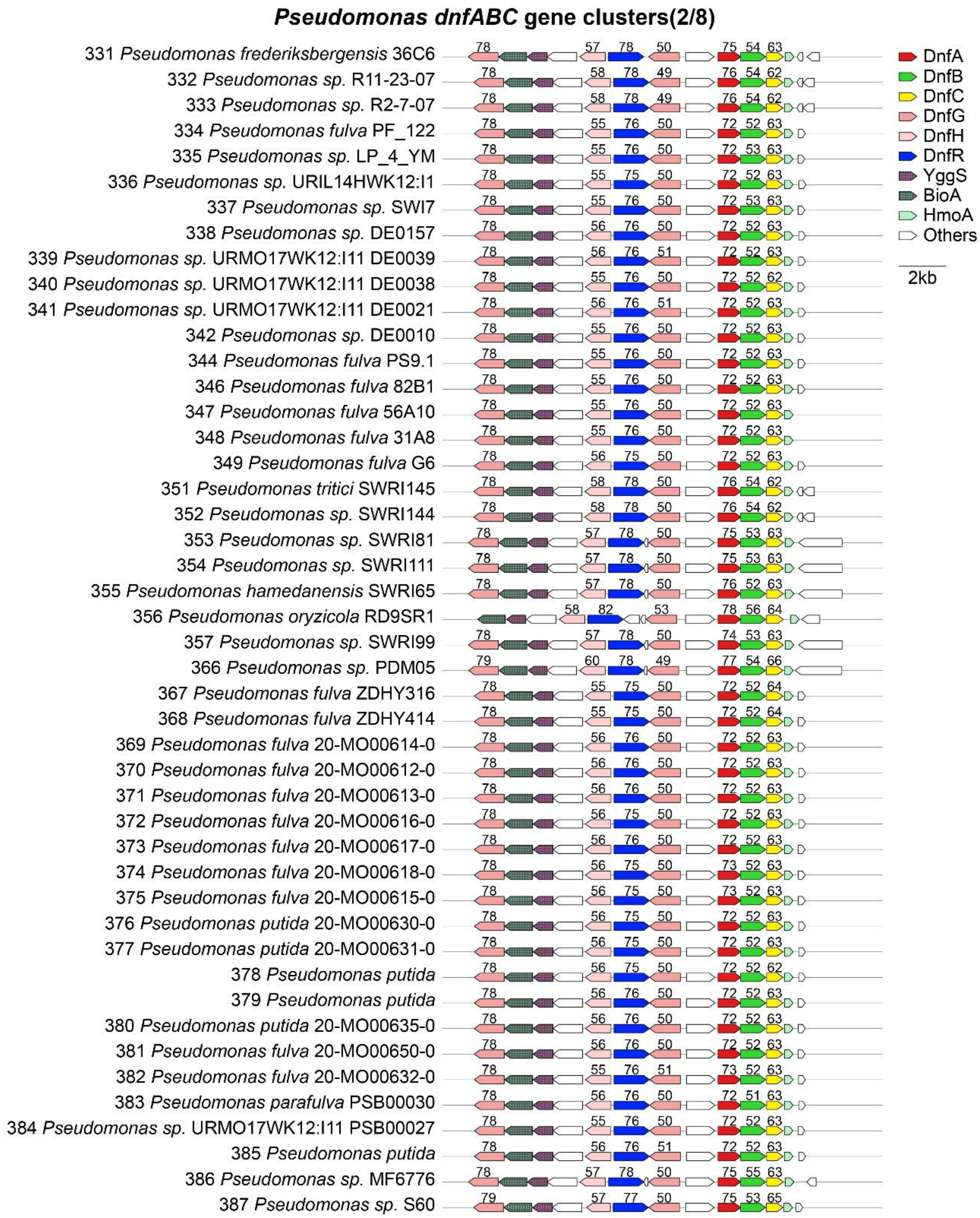

Fig. S9. Comparative organizations of the *dnfABC* gene cluster in genomes of 690 bacterial strains.

Figure S10

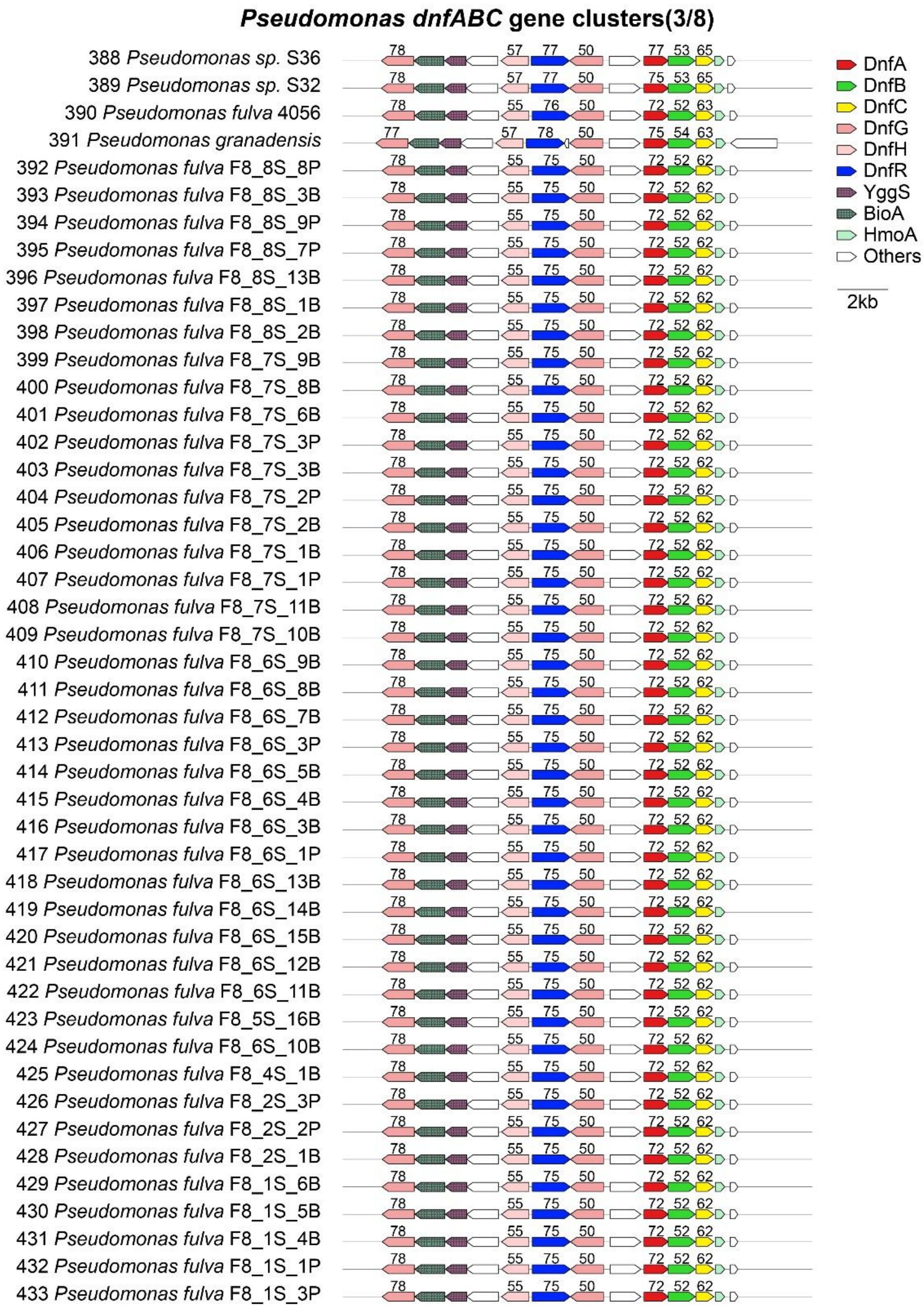

Fig. S10. Comparative organizations of the *dnfABC* gene cluster in genomes of 690 bacterial strains.

Figure S11

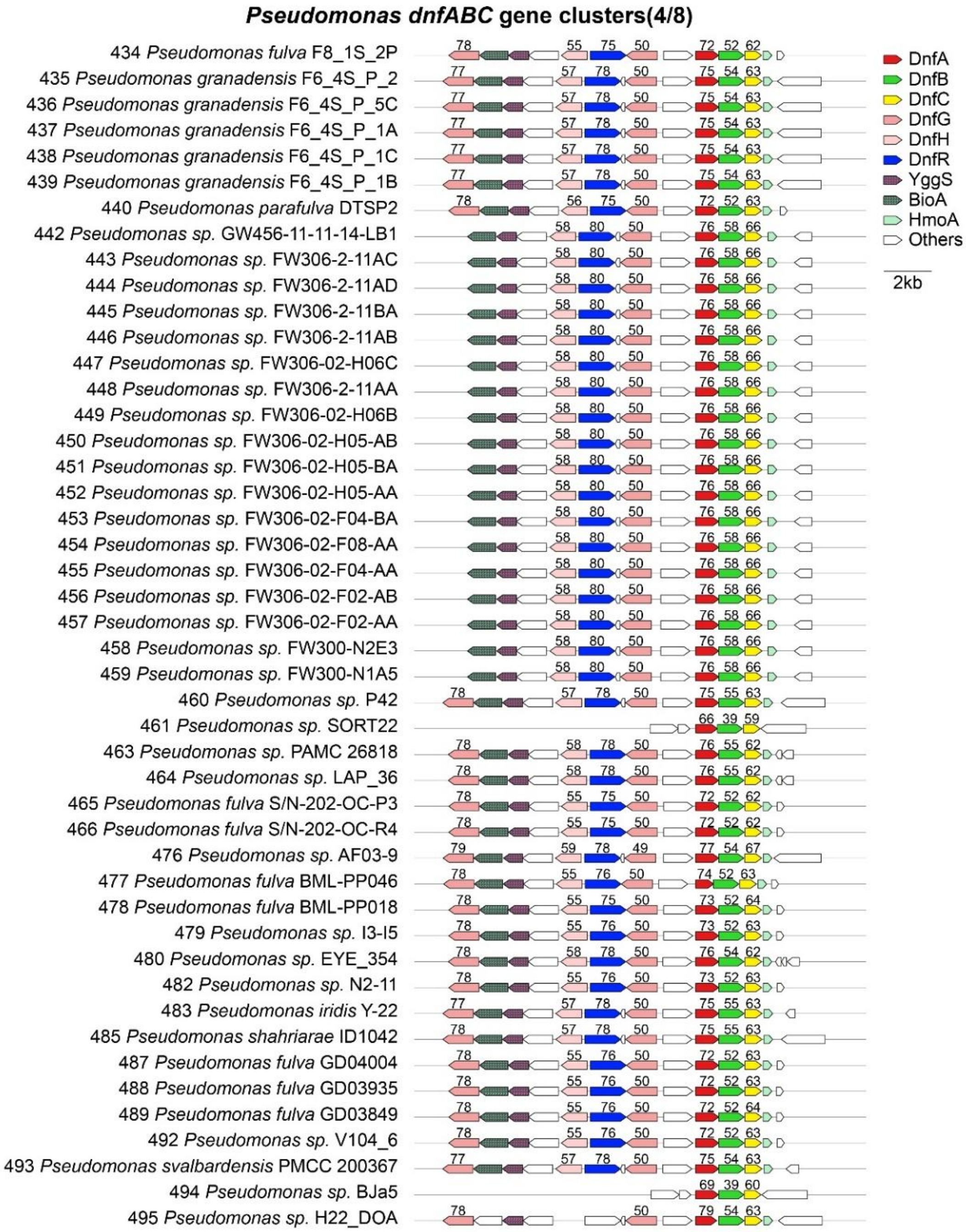

Fig. S11. Comparative organizations of the *dnfABC* gene cluster in genomes of 690 bacterial strains.

Figure S12

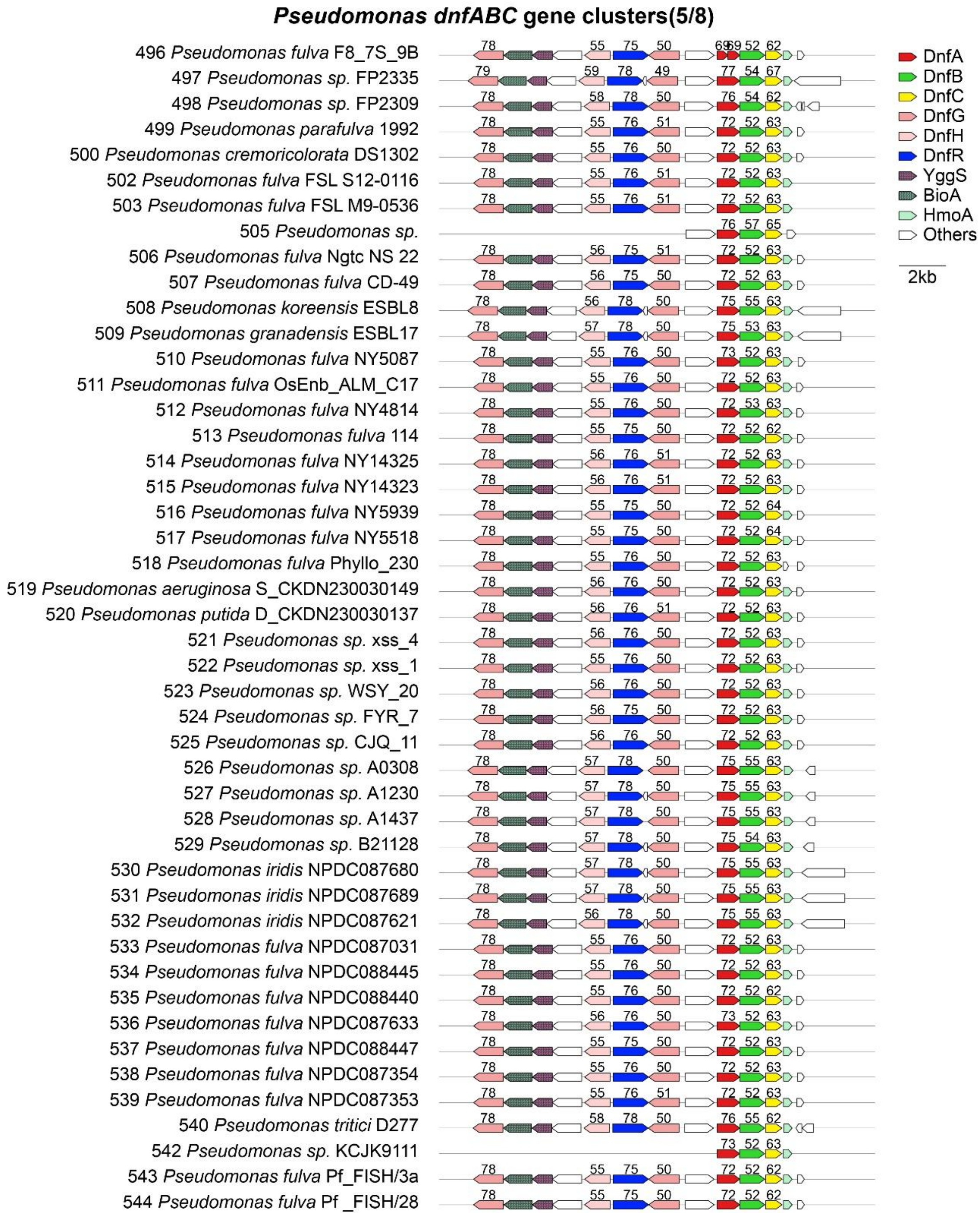

Fig. S12. Comparative organizations of the *dnfABC* gene cluster in genomes of 690 bacterial strains.

Figure S13

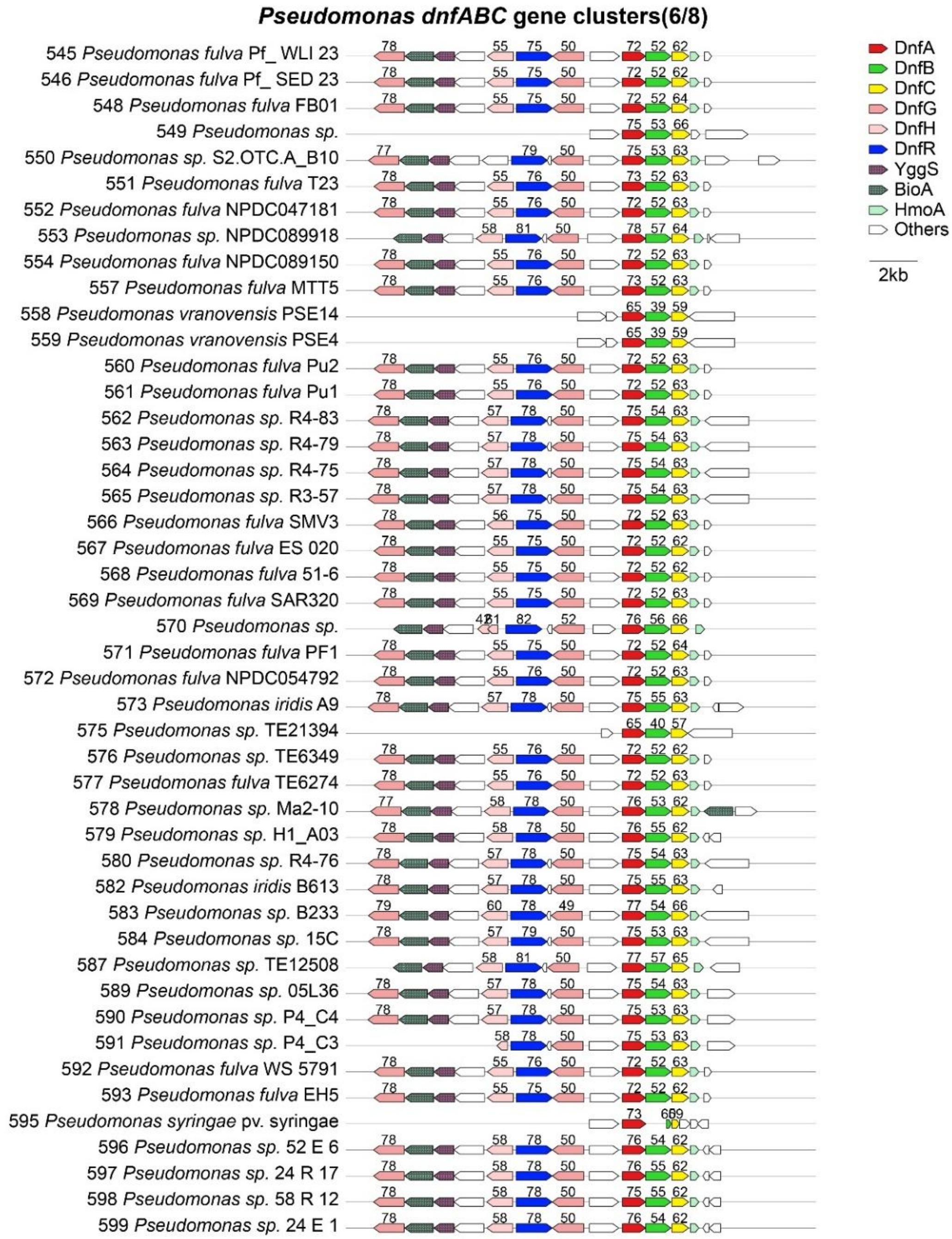

Fig. S13. Comparative organizations of the *dnfABC* gene cluster in genomes of 690 bacterial strains.

Figure S14

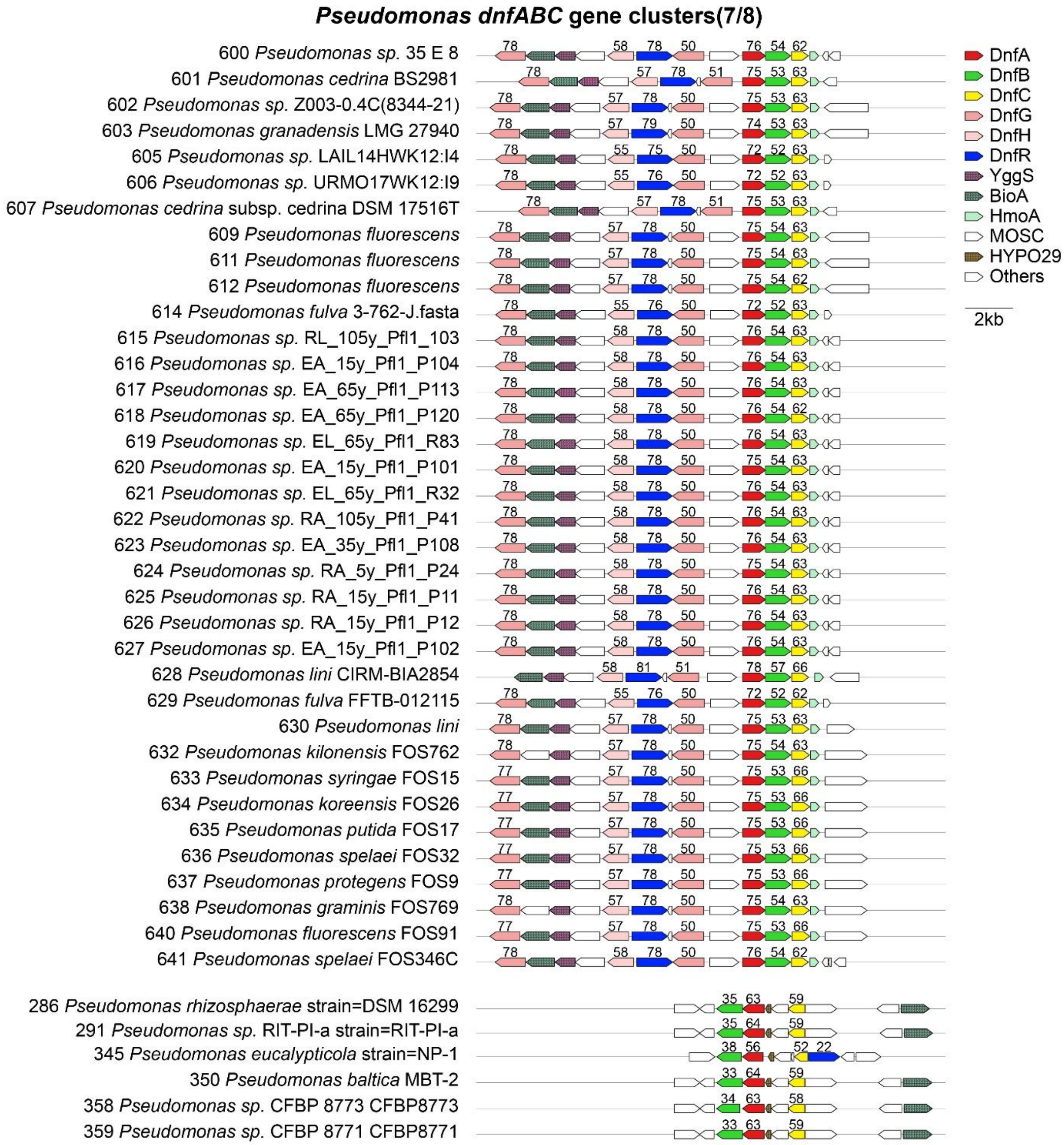

Fig. S14. Comparative organizations of the *dnfABC* gene cluster in genomes of 690 bacterial strains.

Figure S15

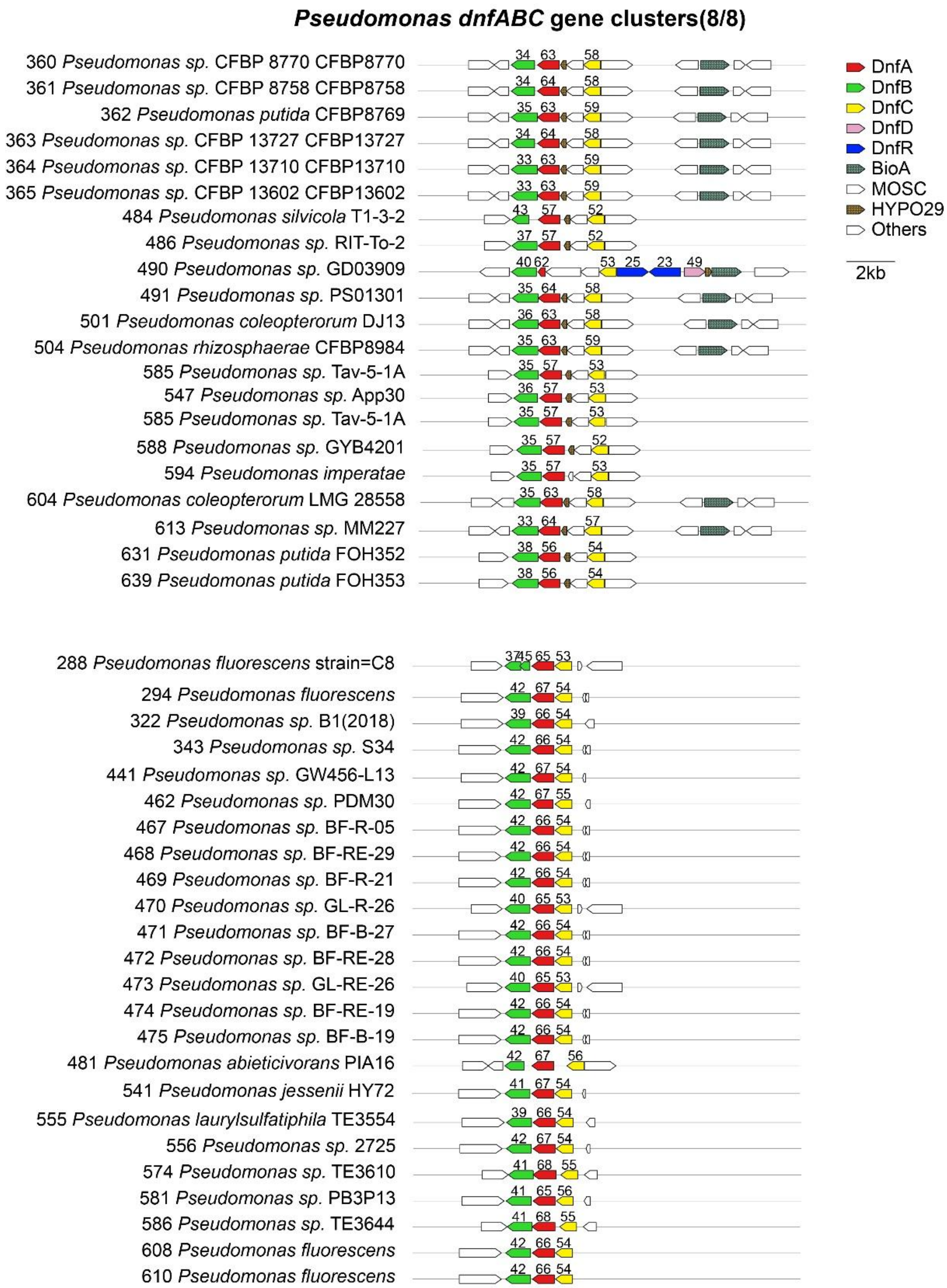

Fig. S15. Comparative organizations of the *dnfABC* gene cluster in genomes of 690 bacterial strains.

Figure S16

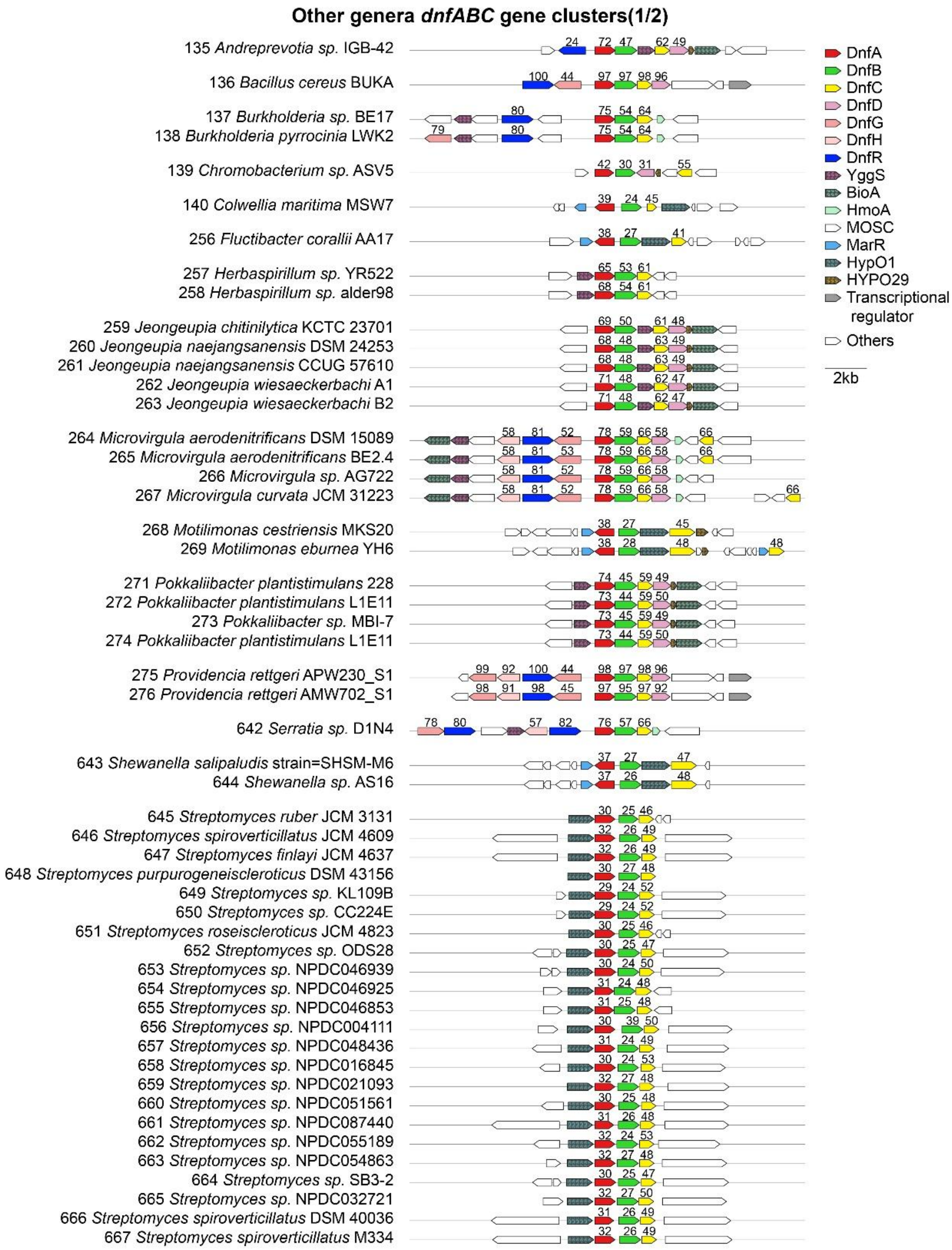

Fig. S16. Comparative organizations of the *dnfABC* gene cluster in genomes of 690 bacterial strains.

Figure S17

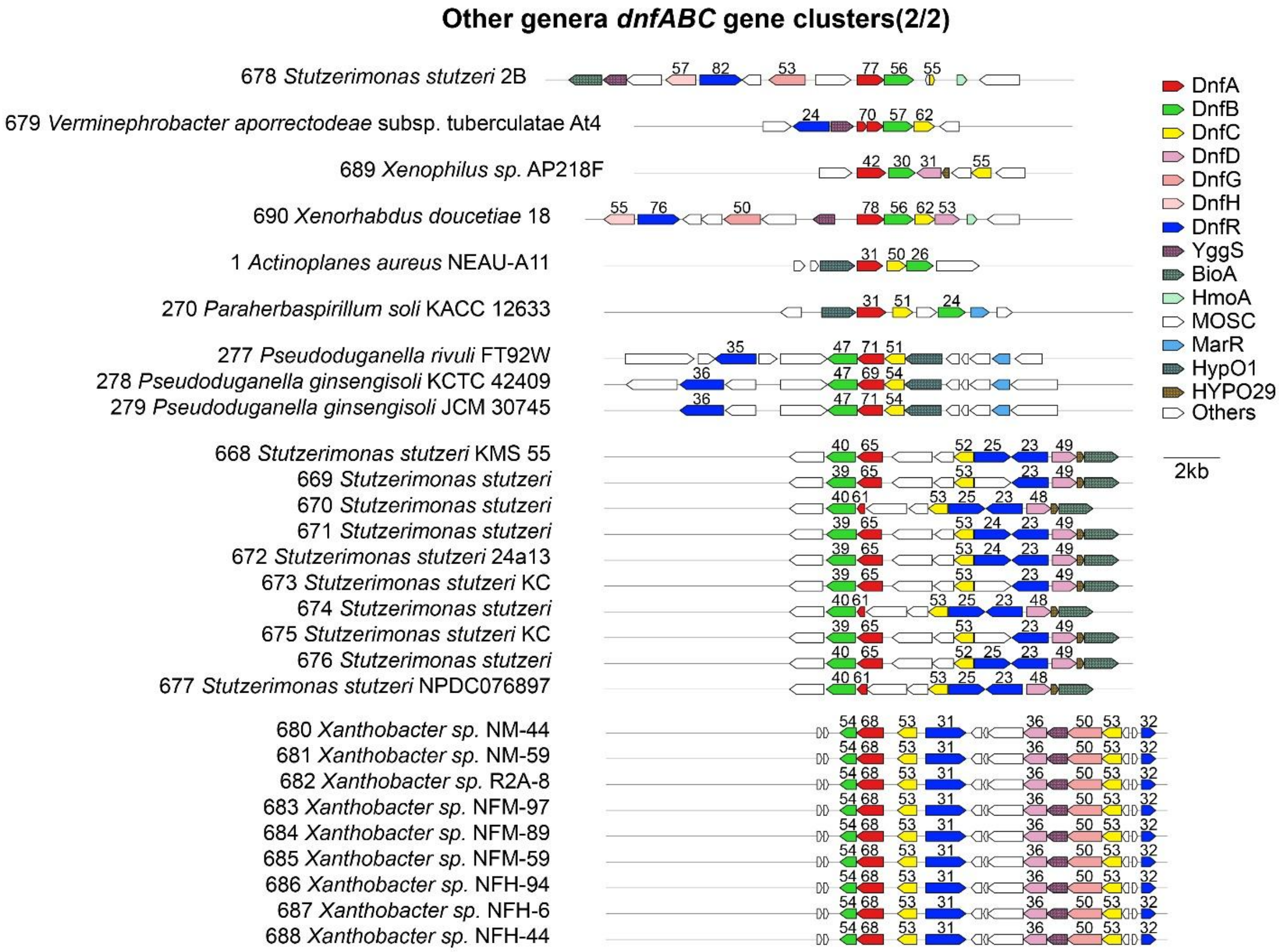

Fig. S17. Comparative organizations of the *dnfABC* gene cluster in genomes of 690 bacterial strains.

Figure S18

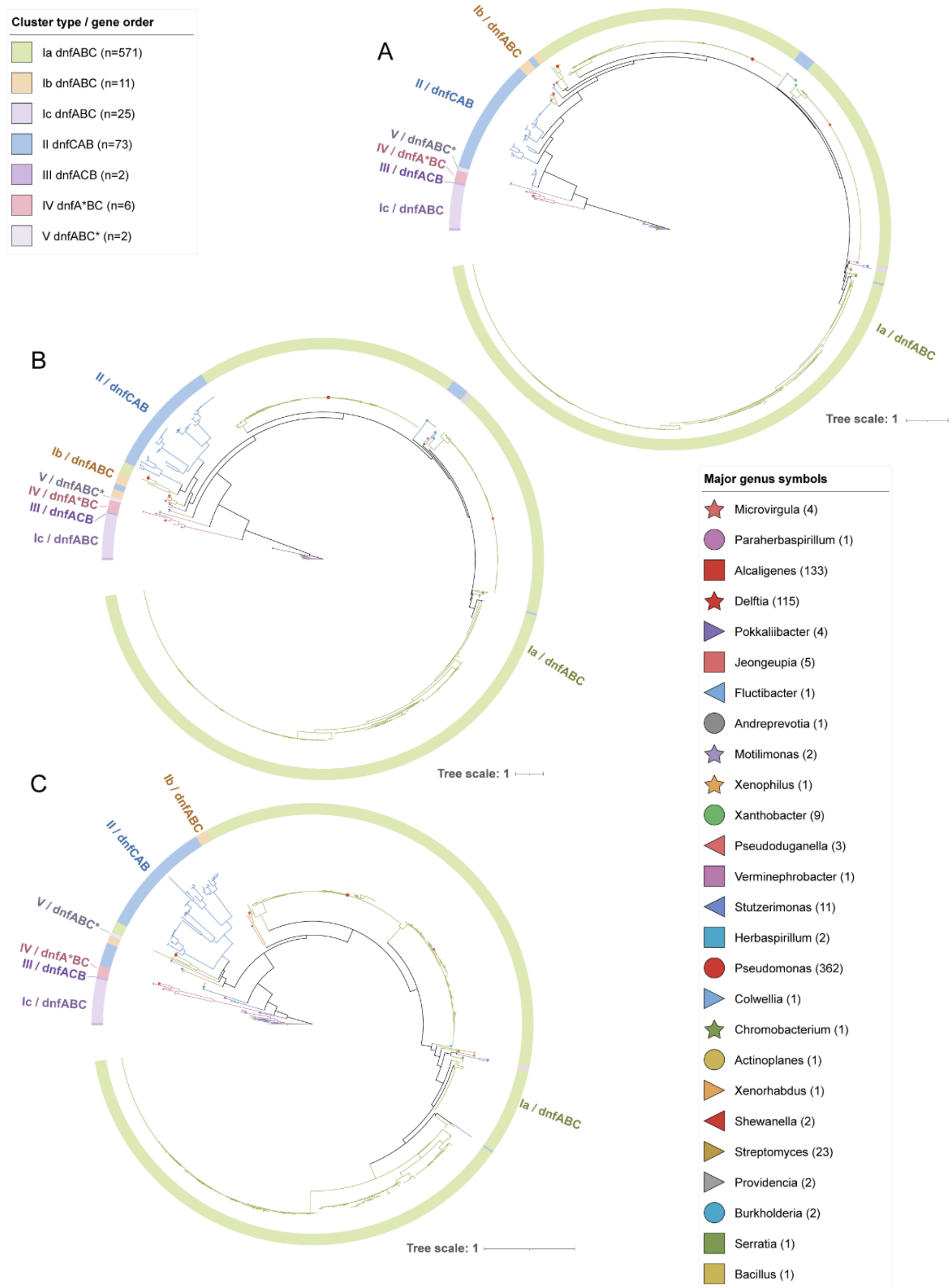

Fig. S18. Phylogenetic trees of representative DnfABC proteins. Maximum likelihood phylogenetic trees were reconstructed from representative amino acid sequences of (A) DnfA, (B) DnfB, and (C) DnfC. The outer ring indicates *dnfABC* cluster type and gene order, including Ia/*dnfABC*, Ib/*dnfABC*, Ic/*dnfABC*, II/*dnfCAB*, III/*dnfACB*, IV/*dnfABC**, and V/*dnfABC**. Colored

branches correspond to the associated cluster types. Symbols on the branches indicate major bacterial genera, and numbers in parentheses indicate the total number of strains from each genus. Red dots indicate experimentally tested strains. Tree scale bars represent amino acid substitutions per site.

Figure S19

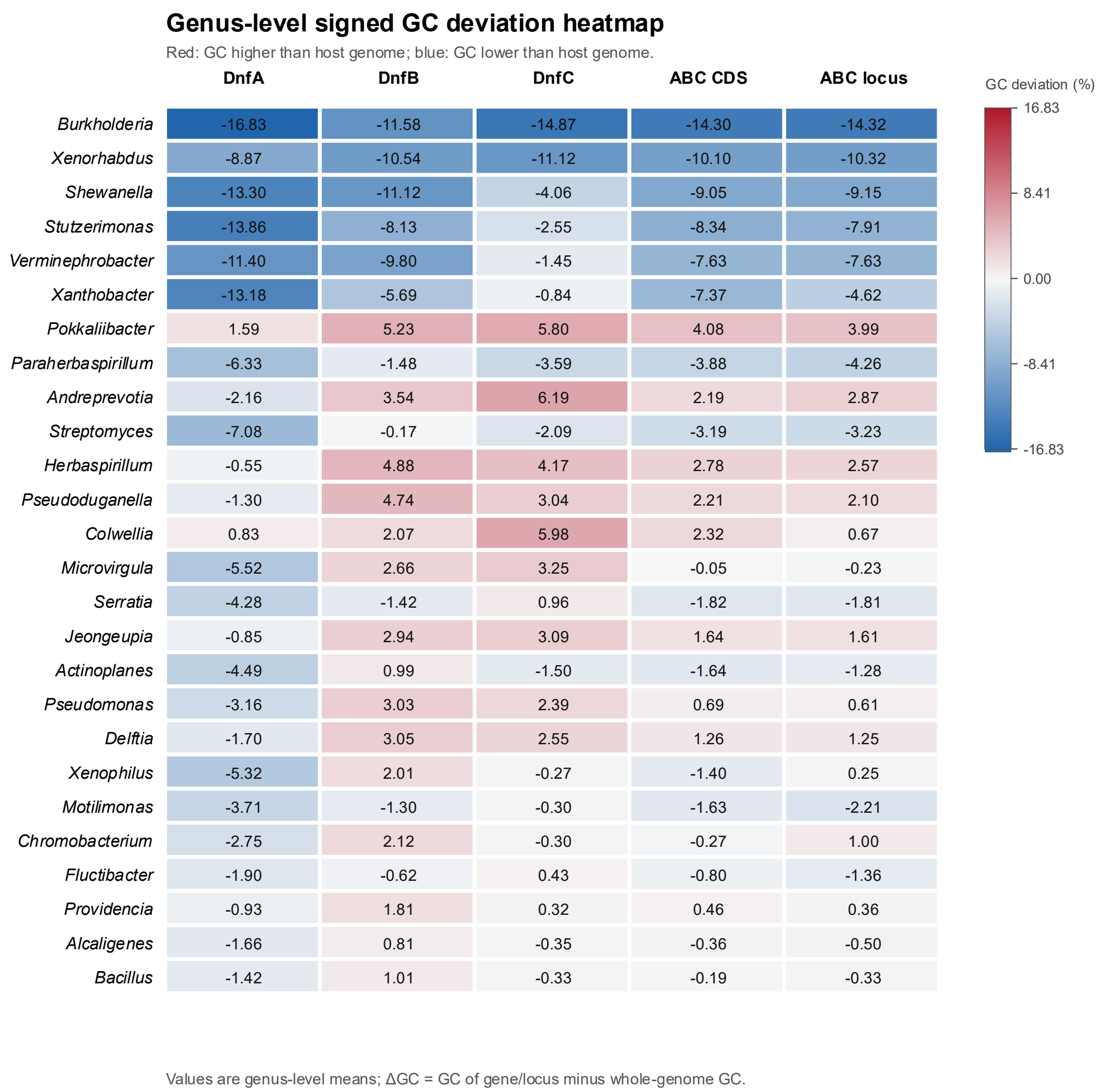

Fig. S19. GC deviation of *dnfABC* genes and loci across genera. Mean signed GC deviation of *dnfA, dnfB, dnfC*, concatenated *dnfABC* CDSs, and complete *dnfABC* loci relative to host genome GC content. Red indicates GC enrichment and blue indicates GC depletion relative to the host genome. Cell values represent genus-level mean deviations in percentage points.

Figure S20

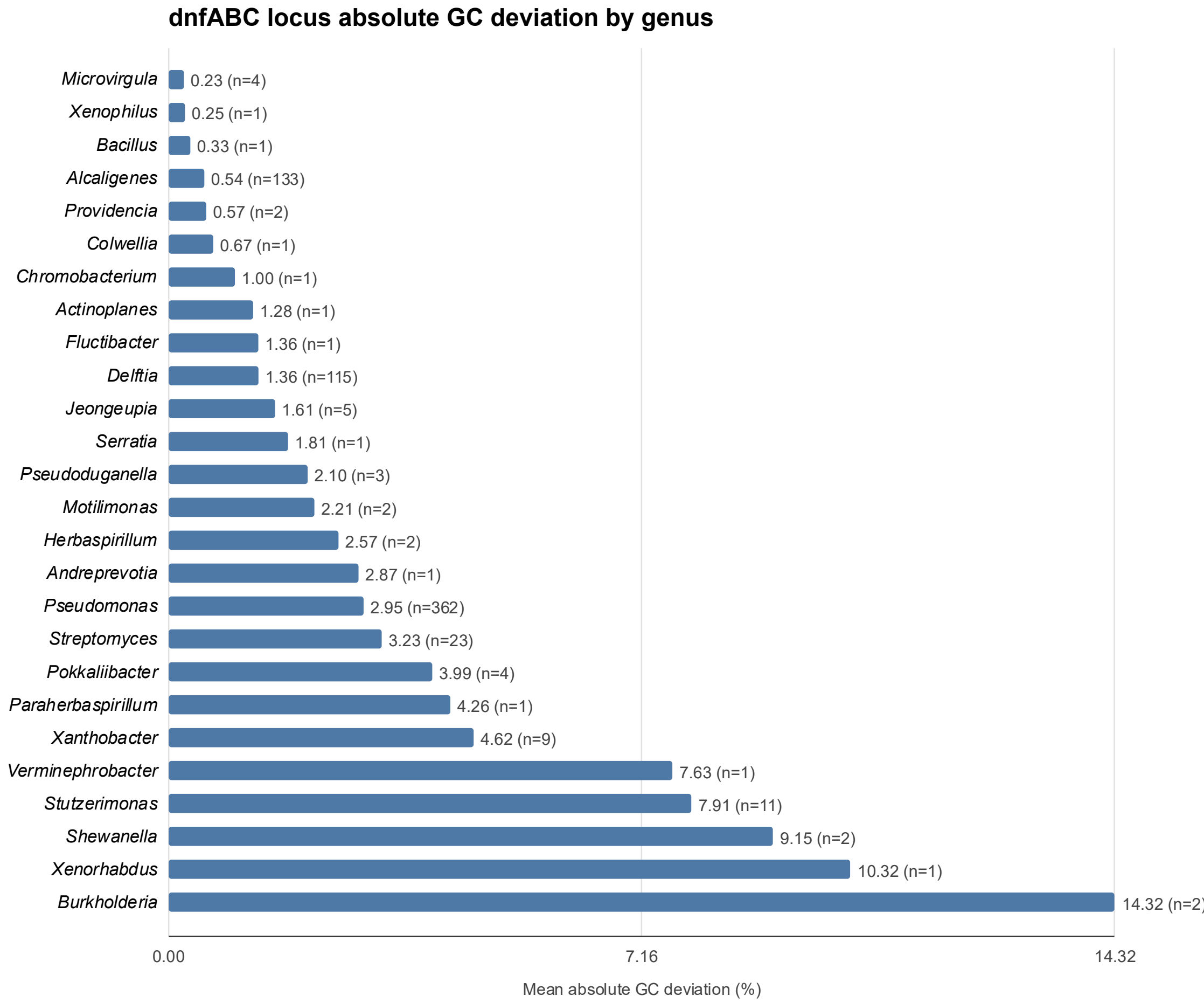

Fig. S20. Mean absolute GC deviation of complete *dnfABC* loci across genera. Genus-level mean absolute GC deviation of complete *dnfABC* loci relative to host genome GC content. Values are shown in percentage points, with the number of analyzed genomes indicated in parentheses.

Figure S21

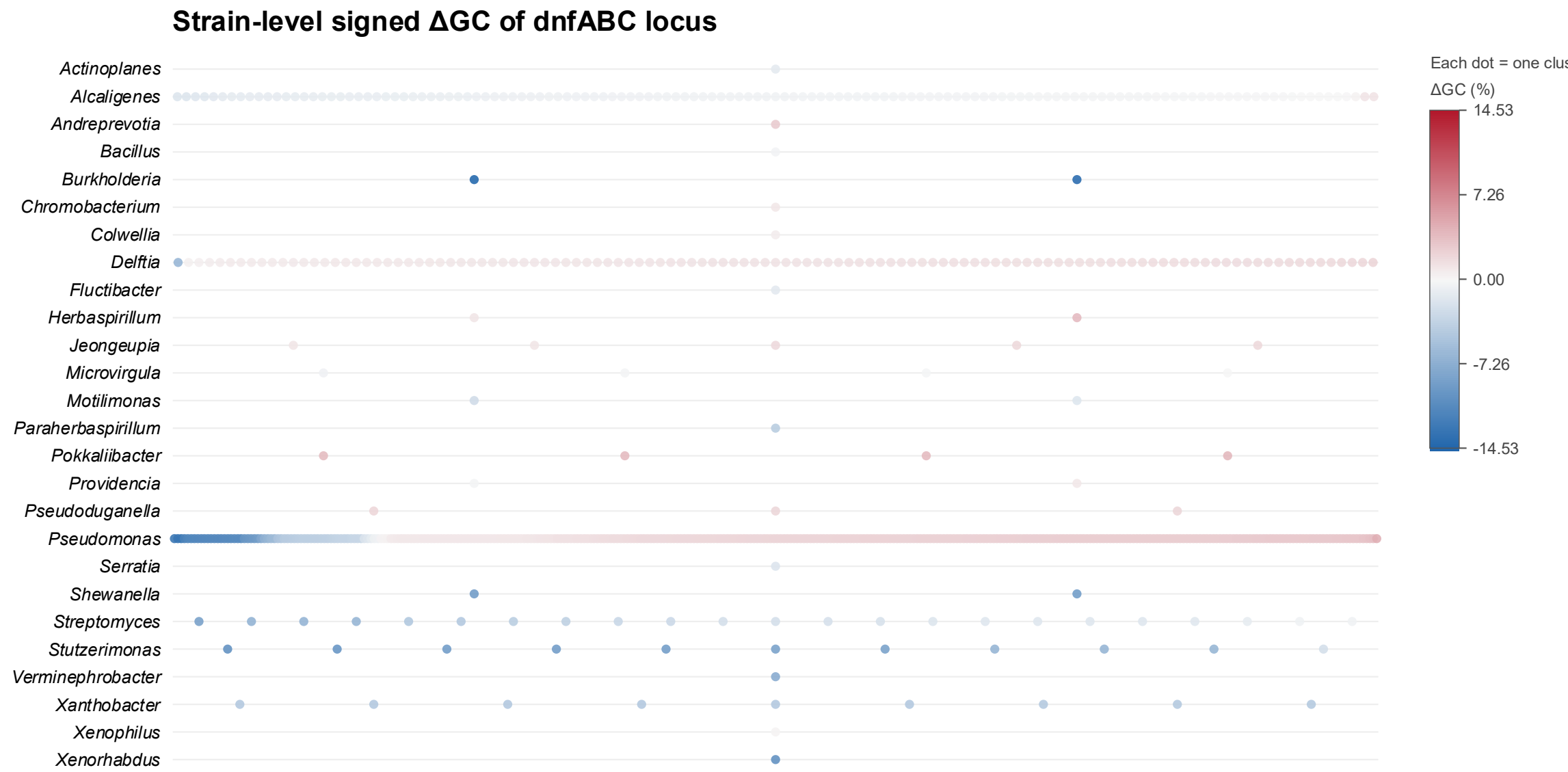

Fig. S21. Cluster-level variation in GC deviation of complete *dnfABC* loci. Each dot represents one complete *dnfABC* locus, with the signed GC deviation calculated as locus GC content minus whole-genome GC content. Red indicates GC enrichment relative to the host genome, blue indicates GC depletion, and white indicates values close to zero. The plot highlights heterogeneity among individual clusters within and across genera.

Figure S22

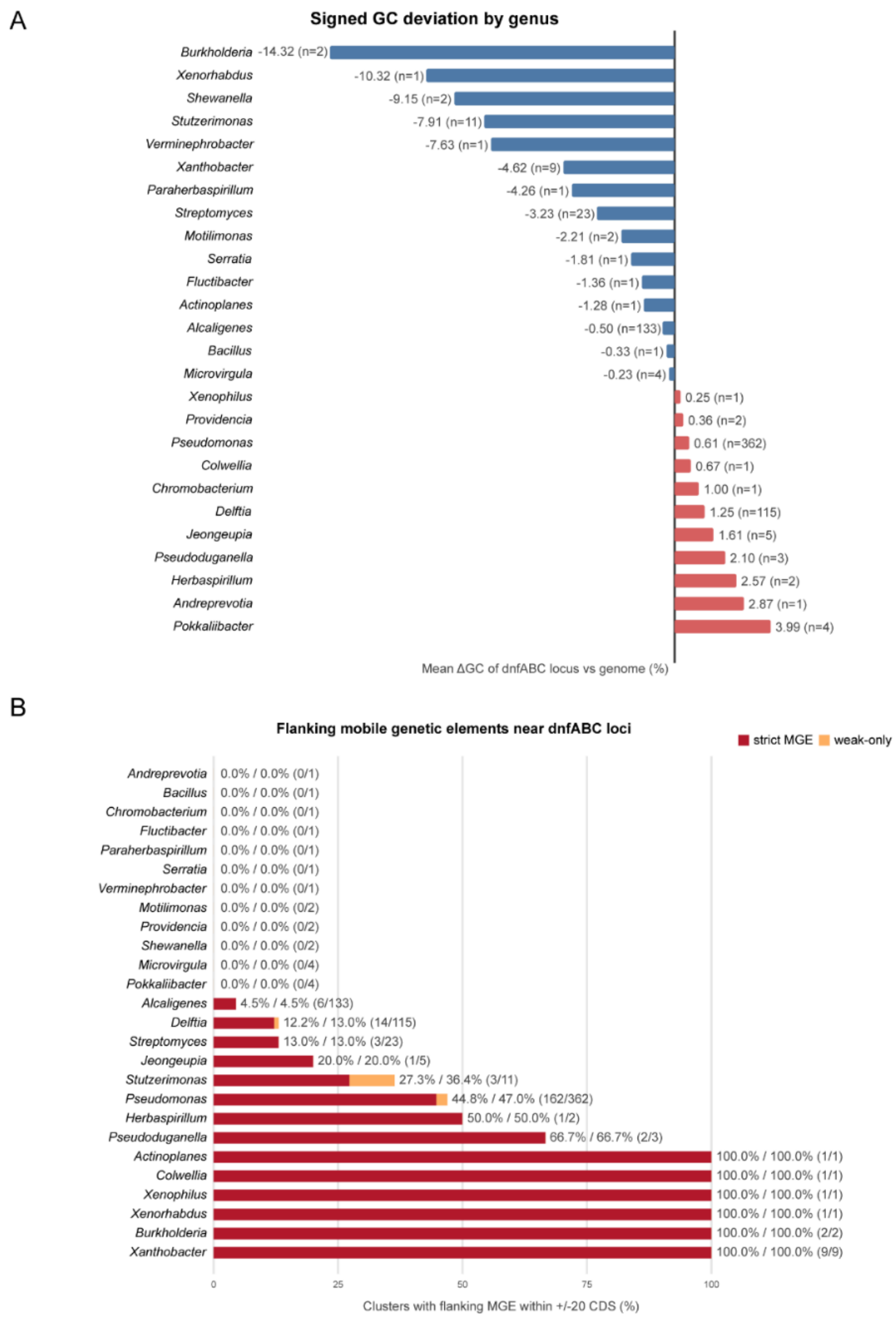

Fig. S22. GC deviation and flanking MGE signals of *dnfABC* loci. (A) Mean signed GC deviation of complete *dnfABC* loci relative to host genomes across genera. Negative and positive values indicate GC depletion and enrichment, respectively. Numbers in parentheses indicate the number of *dnfABC*-containing genomes analyzed for each genus. (B) Frequency of MGE-related genes detected within the 20-CDS flanking regions of *dnfABC* loci across genera. Strict and weak-only MGE signals are shown separately.

Figure S23

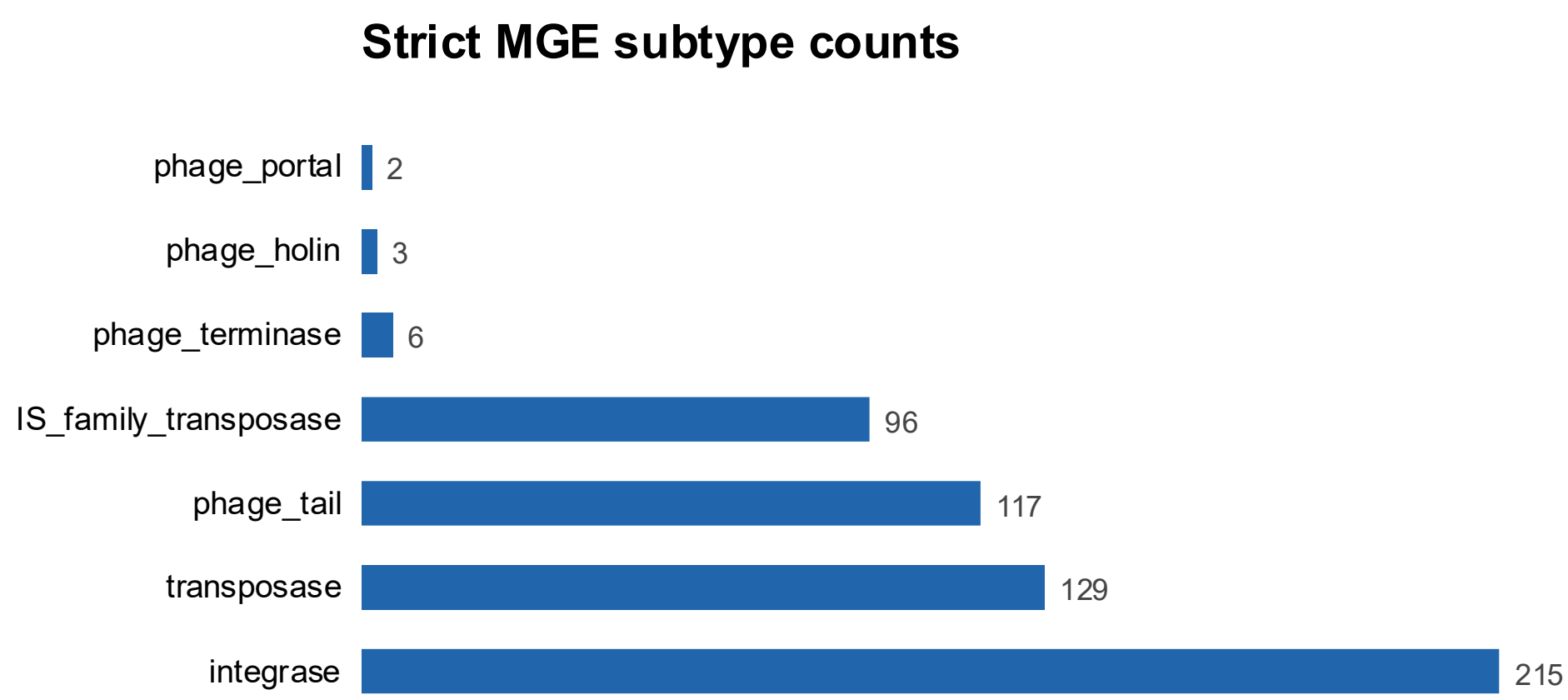

Fig. S23. Strict MGE-related protein categories near *dnfABC* loci. Counts of strict MGE-related protein records detected in the 20-CDS flanking regions of *dnfABC* loci. Integrases, transposases, phage tail proteins, and IS-family transposases were the dominant categories.

Figure S24

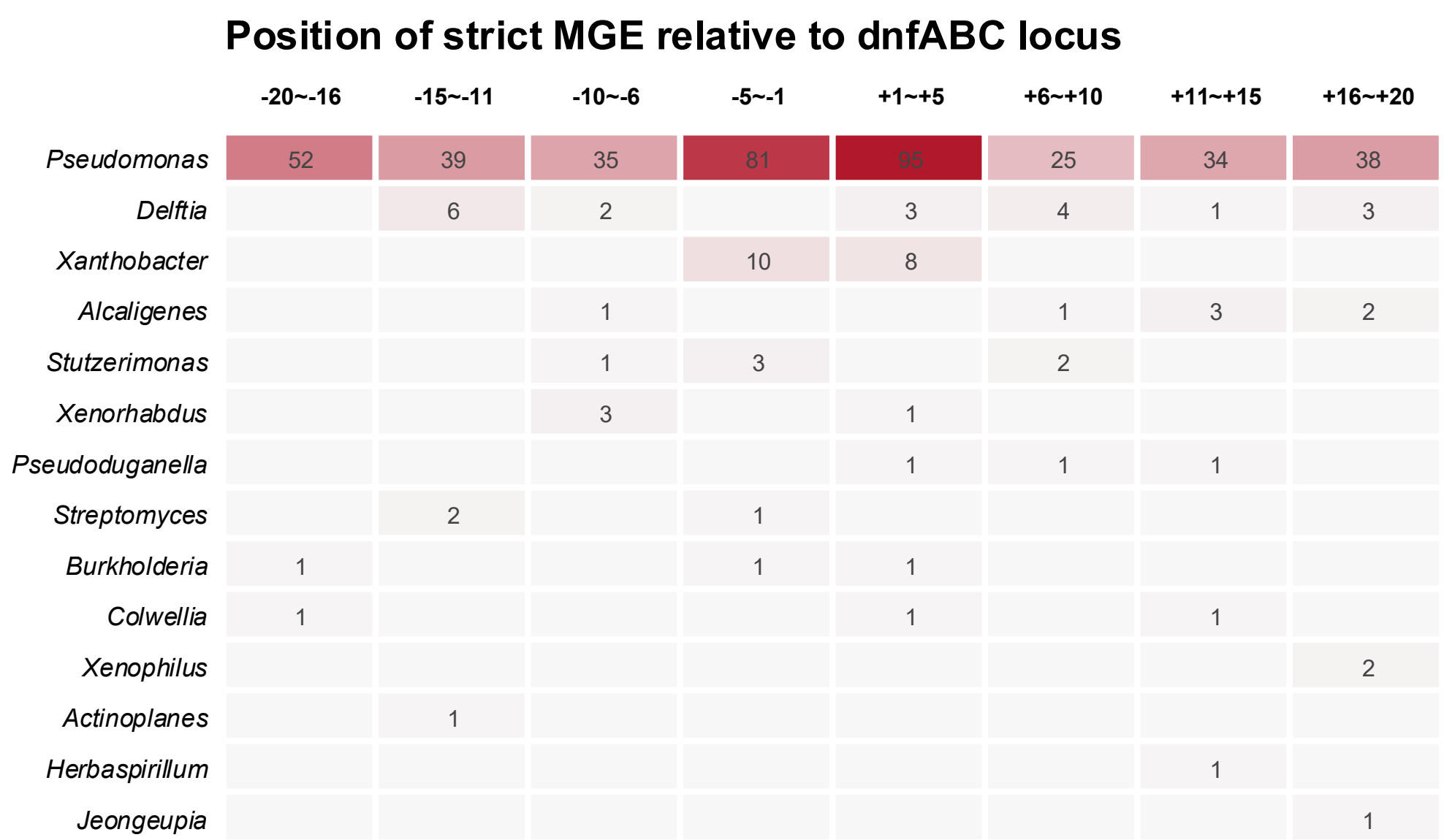

Fig. S24. Position of strict MGE signals around *dnfABC* loci. Heatmap showing the distribution of strict MGE-related protein records within the 20-CDS upstream and downstream flanking regions of *dnfABC* loci. Cell values indicate protein record counts, and darker red shading represents higher frequency.
