# Supplementary material for: Diversity of dirammox microorganisms
Source: mBio. 2026 Oct 15;17(11):e01645-26. doi: 10.1128/mbio.01645-26 (PMC13753816; doi:10.1128/mbio.01645-26)
Supplement: Supplementary file 2 [file mbio.01645-26-s0003.pdf]

Diversity of Dirammox Microorganisms

Gantuya Khasbaatar[1,2#], Qun Han[1,2#], Xiao-Kang Wang[1,2#], Lei Song[3], Chang Liu[1,2], Xin Sun[1,2], Ze-Shen Liu[1], Ying Liu[1], Cheng-Ying Jiang[1], Ji-Guo Qiu[4], Shuang-Jiang Liu[1], Yanning Zheng[1,2*], De-Feng Li[1,2*]

[1] State Key Laboratory of Microbial Diversity and Innovative Utilization, Institute of Microbiology, Chinese Academy of Sciences, Beijing 100101, China

[2] College of Life Sciences, University of Chinese Academy of Sciences, Beijing 100049, China

[3] China General Microbiological Culture Collection Center, Institute of Microbiology, Chinese Academy of Sciences, Beijing 100101, China

[4] Key Laboratory of Agricultural and Environmental Microbiology, Ministry of Agriculture and Rural Affairs, College of Life Sciences, Nanjing Agricultural University, Nanjing 210095, China

[#] Those authors contribute equally.

***Correspondences:**

De-Feng Li: lidefeng@im.ac.cn, Tel: +86-10-64807015.

Yanning Zheng: zhengyn@im.ac.cn, Tel: +86-10-64806943

Running title: Diversity of Dirammox Microorganisms

This Supplementary Information file includes: Figure S25-S36, Table S1, S2, and S3 (Excel file).

Table of Contents

Fig. S25. Major nitrogen cycling pathways……………………..……………………...…3-4

Fig. S26. Nitrogen metabolism type I: genomes containing only the dirammox pathway……….5

Fig. S27. Nitrogen metabolism type II: genomes containing dirammox and denitrification pathways……………………………………………………..……………………………6

Fig. S28. Nitrogen metabolism type III: genomes containing dirammox and ANRA pathways….7

Fig. **S29.** Nitrogen metabolism type IV: genomes containing dirammox, DNRA, and ANRA pathways……………………………………………………………………………………..8

Fig. S30. Nitrogen metabolism type V: genomes containing dirammox and DNRA pathways…..9

Fig. S31. Nitrogen metabolism type VI: genomes containing dirammox, DNRA, and denitrification pathways……………………………………………………………………….10

Fig. S32. Nitrogen metabolism type VII: genomes containing dirammox, ANRA, and nitrogen fixation pathways……………………………………………………………………...………11

Fig. S33. Nitrogen metabolism type VIII: genomes containing dirammox, DNRA, ANRA, and denitrification pathways……………………………………………………………………….12

Fig. S34. Nitrogen metabolism type IX: genomes containing dirammox, DNRA, ANRA, denitrification, and nitrogen fixation pathways…………………………………...…………..13

Fig. S35. Hydroxylamine accumulation and growth of seven wild-type strains on ammonia and L-glutamine as nitrogen sources………………………………………………………...……..14

Fig. S36. Hydroxylamine accumulation and growth of *Delftia* and *Alcaligenes* strains on ammonia……………………………………………………………………………………….15

Table S1. All strains were used in this study…………………………………………………..…16

Table S2. All plasmids and primers were used in this study…………………………………….17

Figure S25

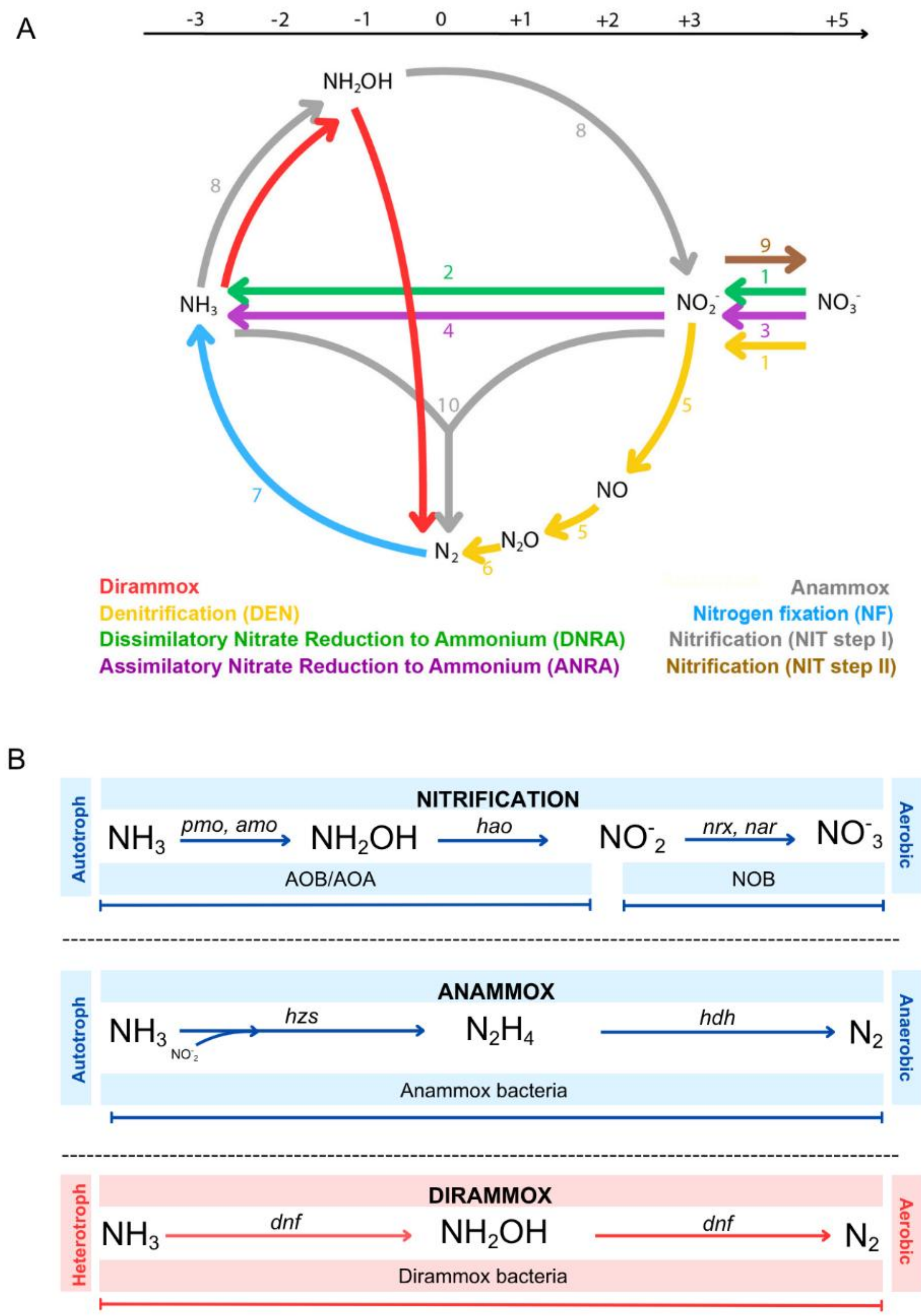

Fig. S25. Major nitrogen cycling pathways. (A) Schematic overview of major nitrogen cycling pathways analyzed in this study. Colored arrows represent distinct pathways: dirammox (red), DNRA (green), ANRA (purple), denitrification (yellow), nitrogen fixation (blue), and nitrification step II (brown). Nitrification step I and anammox, which were not detected in the analyzed strains,

are shown in grey. Numbers correspond to the ten pathways illustrated. Oxidation states of nitrogen species (–3 to +5) are indicated at the top, highlighting the redox transitions associated with each transformation. (B**)** Overview of microbial ammonia oxidation pathways showing key marker genes. In nitrification (aerobic, autotrophic), NH_3 is oxidized to NH_2OH by *amo/pmo*, further to NO_2^- by *hao*, and to NO_3^- by nitrite-oxidizing bacteria (NOB) via *nrx/nar*. In anammox (anaerobic, autotrophic), NH_3 and NO_2^- are converted to N_2 via N_2H_4, mediated by *hzs* and *hdh*. In dirammox (aerobic, heterotrophic), NH_3 is converted to NH_2OH and subsequently to N_2, associated with the *dnf* gene cluster.

Figure S26

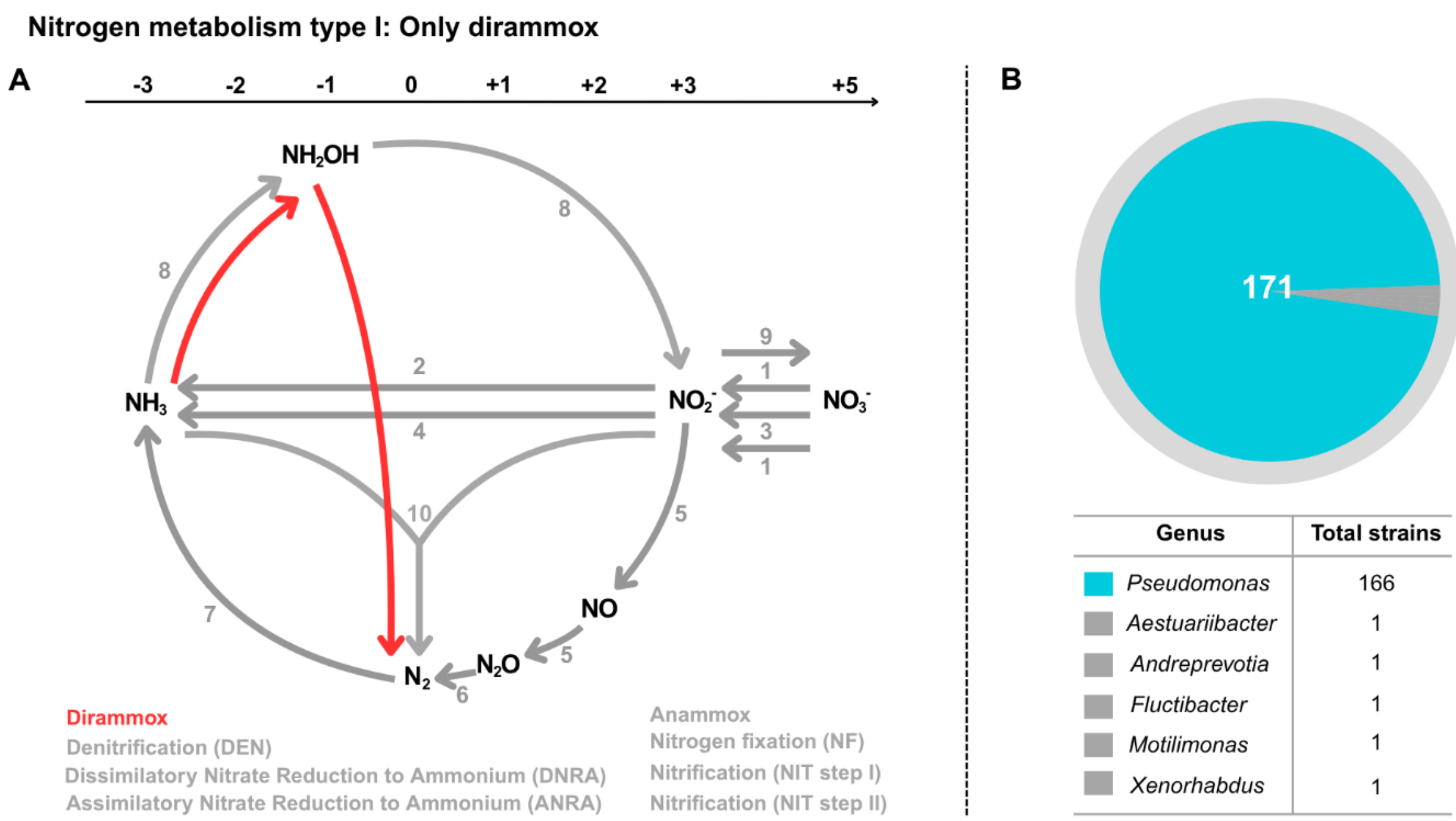

Fig. S26. Nitrogen metabolism type I: genomes containing only the dirammox pathway. (A) The dirammox pathway is shown in red, and other nitrogen metabolism pathways are shown in gray. (B) Taxonomic distribution of type I genomes. Genera represented by two or fewer genomes were shown in gray.

Figure S27

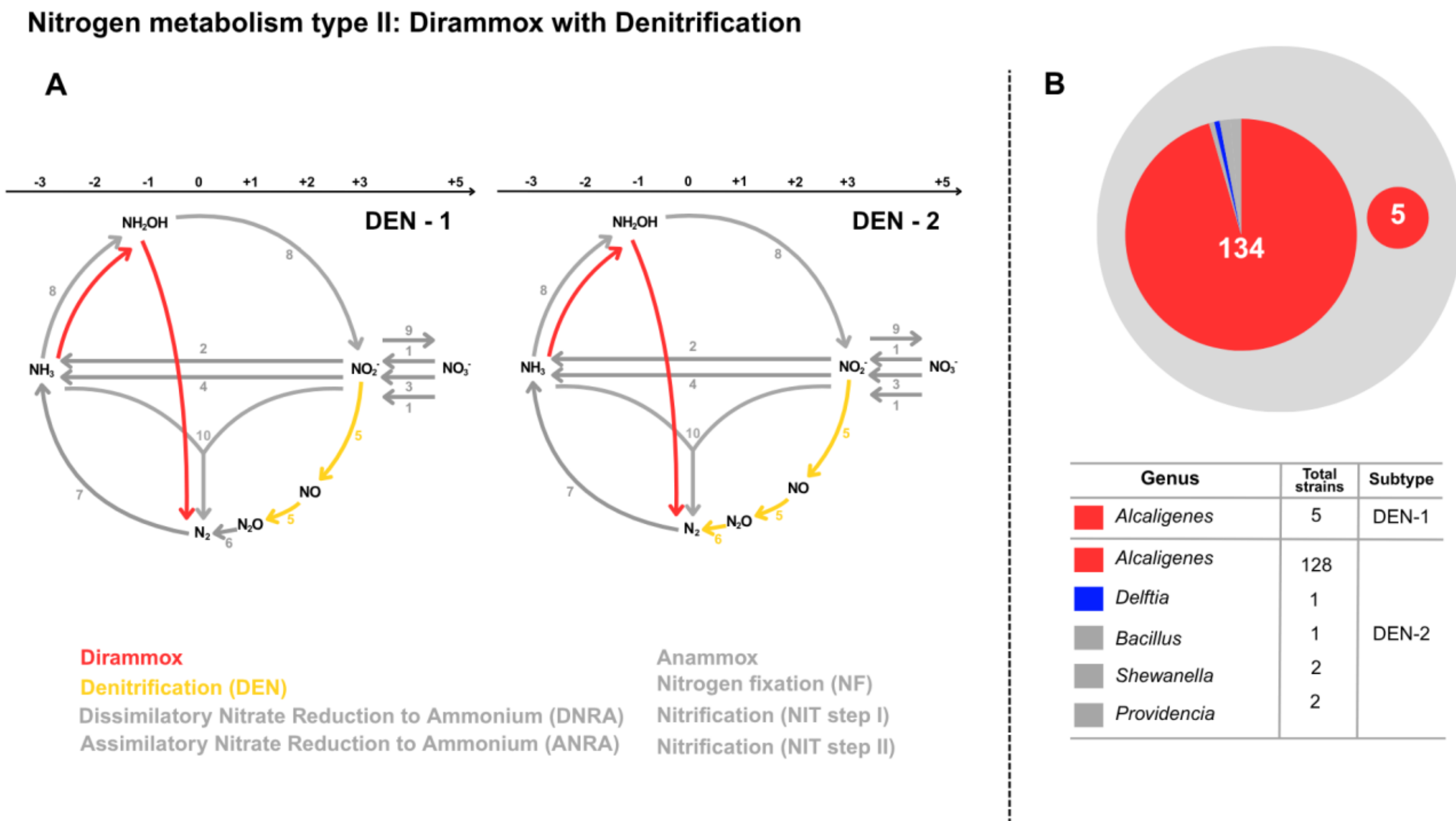

Fig. S27. Nitrogen metabolism type II: genomes containing dirammox and denitrification pathways. (A) The dirammox pathway is shown in red, the denitrification pathway is shown in yellow, and other nitrogen metabolism pathways are shown in gray. (B) Taxonomic distribution and subtype composition of type II genomes. Genera represented by two or fewer genomes were shown in gray.

Figure S28

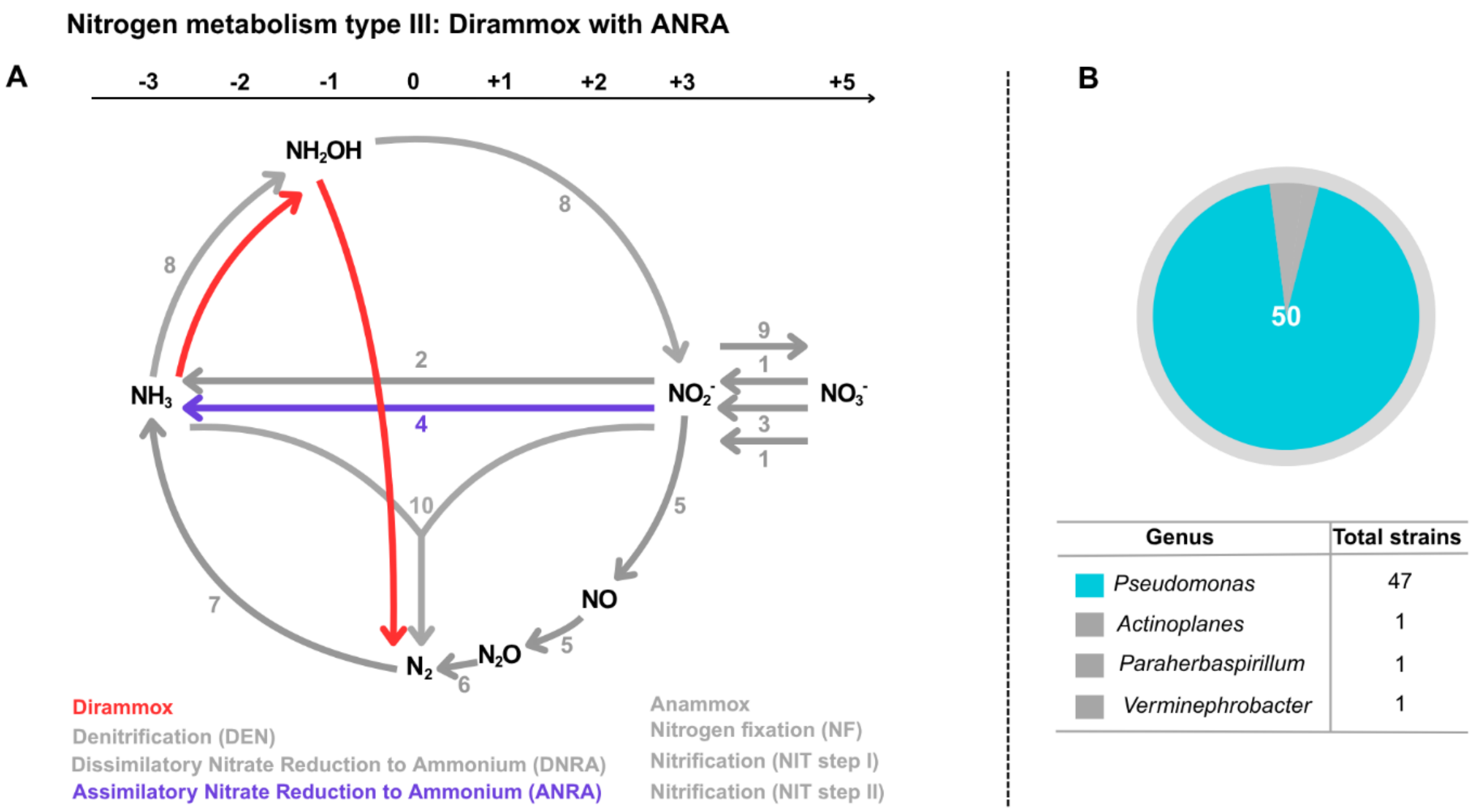

Fig. S28. Nitrogen metabolism type III: genomes containing dirammox and ANRA pathways. (A) The dirammox pathway is shown in red, the assimilatory nitrate reduction to ammonium pathway is shown in purple, and other nitrogen metabolism pathways are shown in gray. (B) Taxonomic distribution of type III genomes. Genera represented by two or fewer genomes were shown in gray.

Figure S29

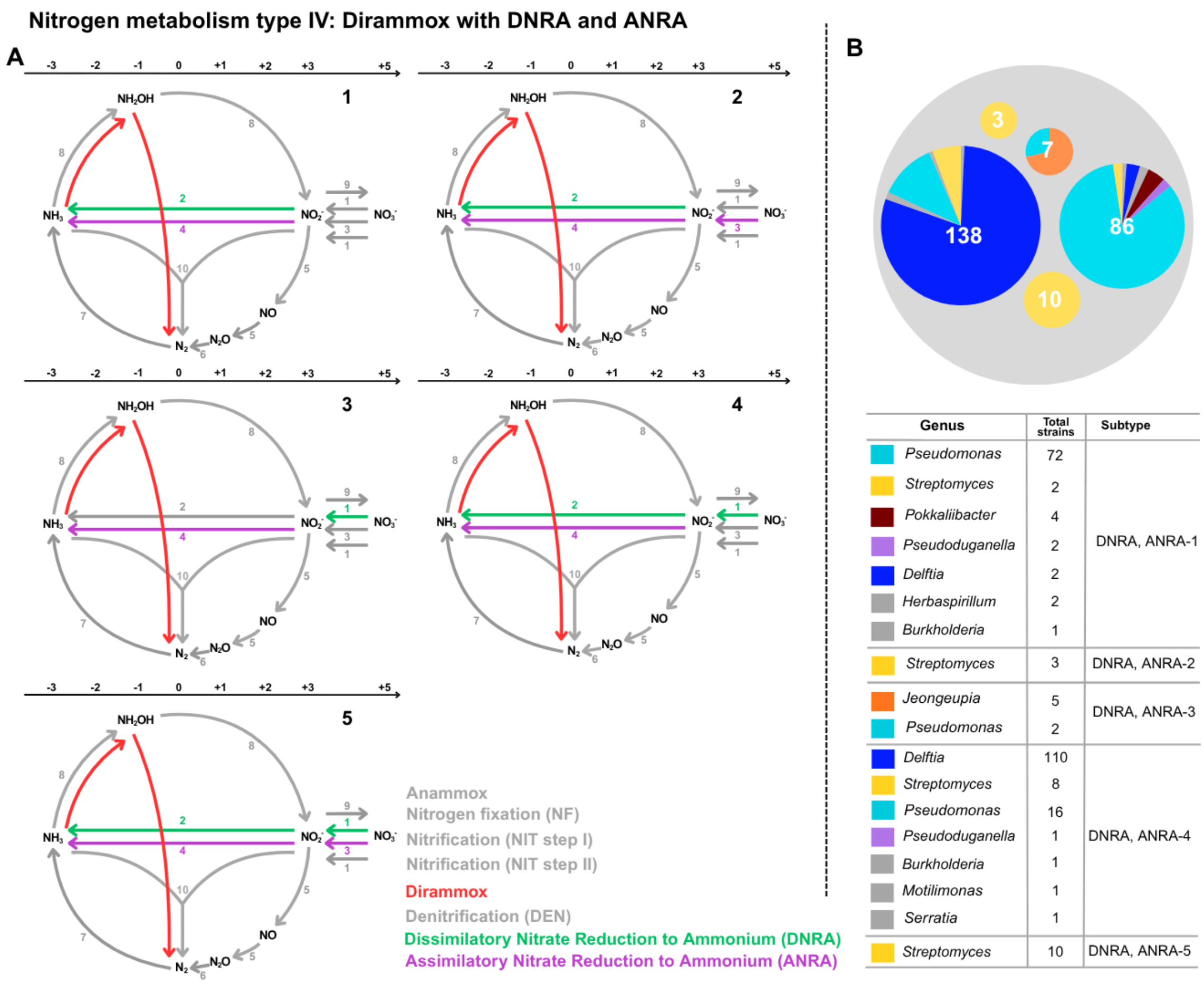

Genus	Total strains	Subtype
Pseudomonas	72	DNRA, ANRA-1
Streptomyces	2	
Pokkaliibacter	4	
Pseudoduganella	2	
Delftia	2	
Herbaspirillum	2	
Burkholderia	1	
Streptomyces	3	DNRA, ANRA-2
Jeongeupia	5	DNRA, ANRA-3
Pseudomonas	2	
Delftia	110	DNRA, ANRA-4
Streptomyces	8	
Pseudomonas	16	
Pseudoduganella	1	
Burkholderia	1	
Motilimonas	1	
Serratia	1	
Streptomyces	10	DNRA, ANRA-5

Fig. S29. Nitrogen metabolism type IV: genomes containing dirammox, DNRA, and ANRA pathways. (A) The dirammox pathway is shown in red, dissimilatory nitrate reduction to ammonium is shown in green, assimilatory nitrate reduction to ammonium is shown in purple, and other nitrogen metabolism pathways are shown in gray. (B) Taxonomic distribution and subtype composition of type IV genomes. Genera represented by two or fewer genomes were shown in gray.

Figure S30

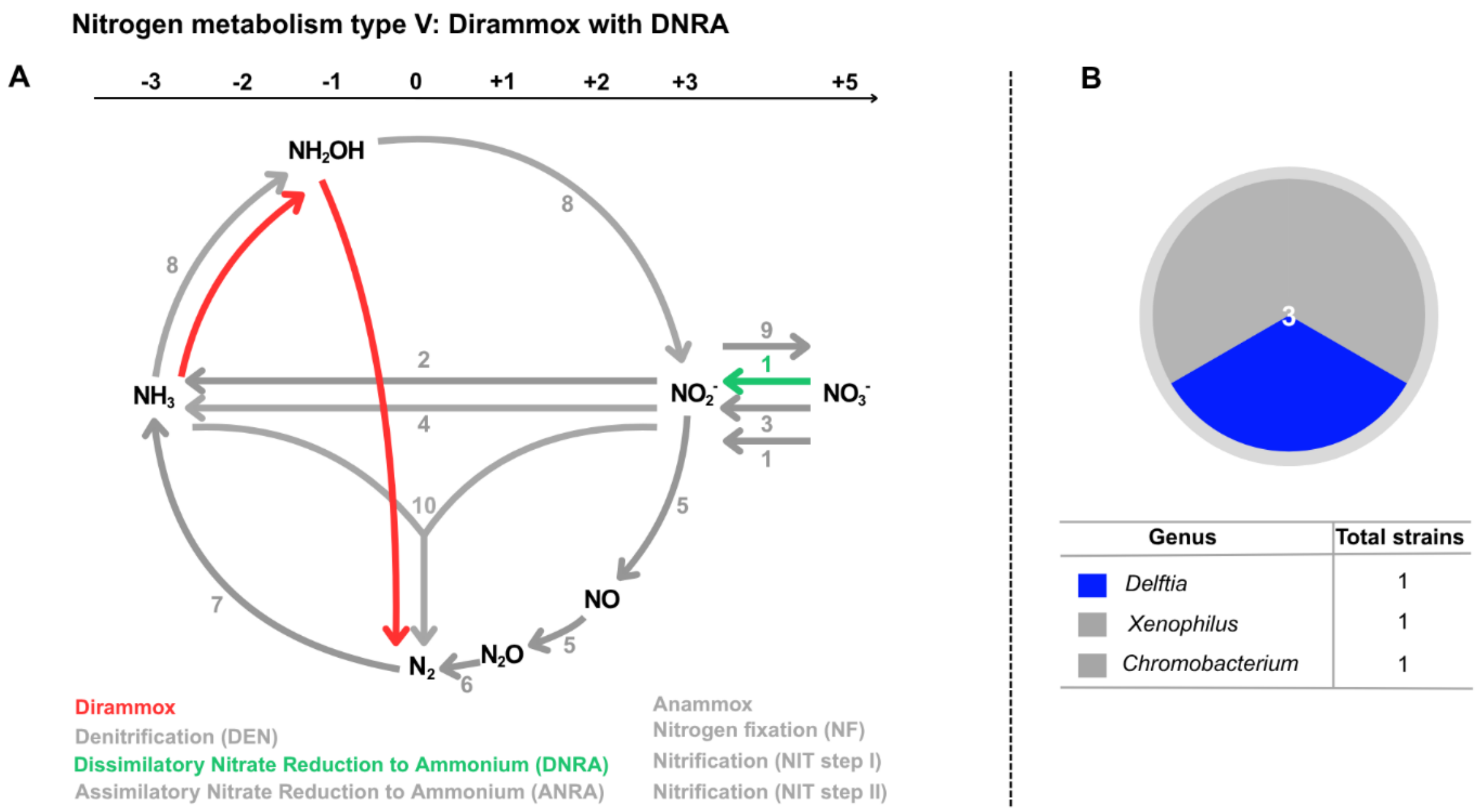

Fig. S30. Nitrogen metabolism type V: genomes containing dirammox and DNRA pathways. (A) The dirammox pathway is shown in red, dissimilatory nitrate reduction to ammonium is shown in green, and other nitrogen metabolism pathways are shown in gray. (B) Taxonomic distribution of type V genomes. Genera represented by two or fewer genomes were shown in gray.

Figure S31

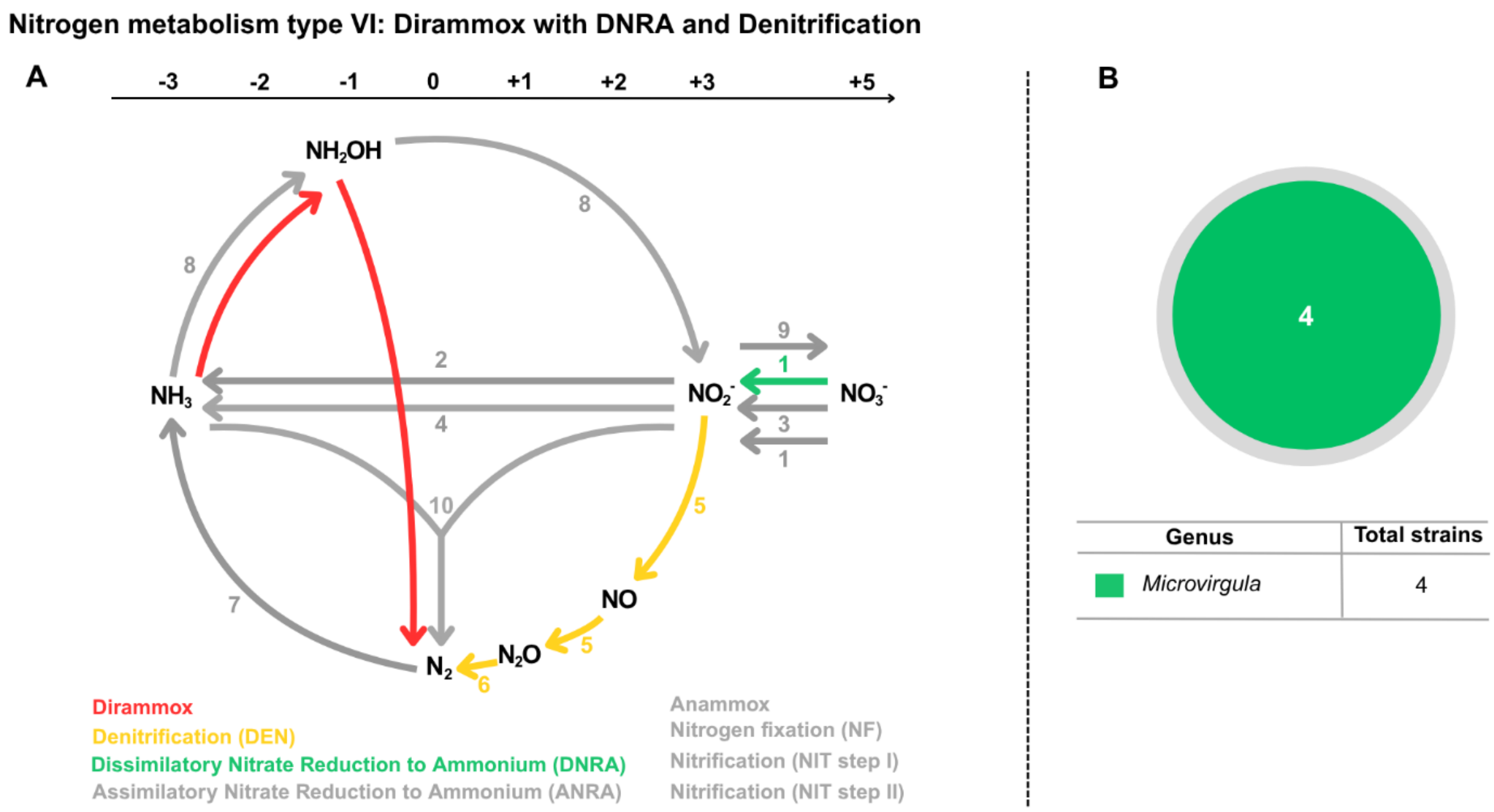

Fig. S31. Nitrogen metabolism type VI: genomes containing dirammox, DNRA, and denitrification pathways. (A) The dirammox pathway is shown in red, denitrification is shown in yellow, dissimilatory nitrate reduction to ammonium is shown in green, and other nitrogen metabolism pathways are shown in gray. (B) Taxonomic distribution of type VI genomes.

Figure S32

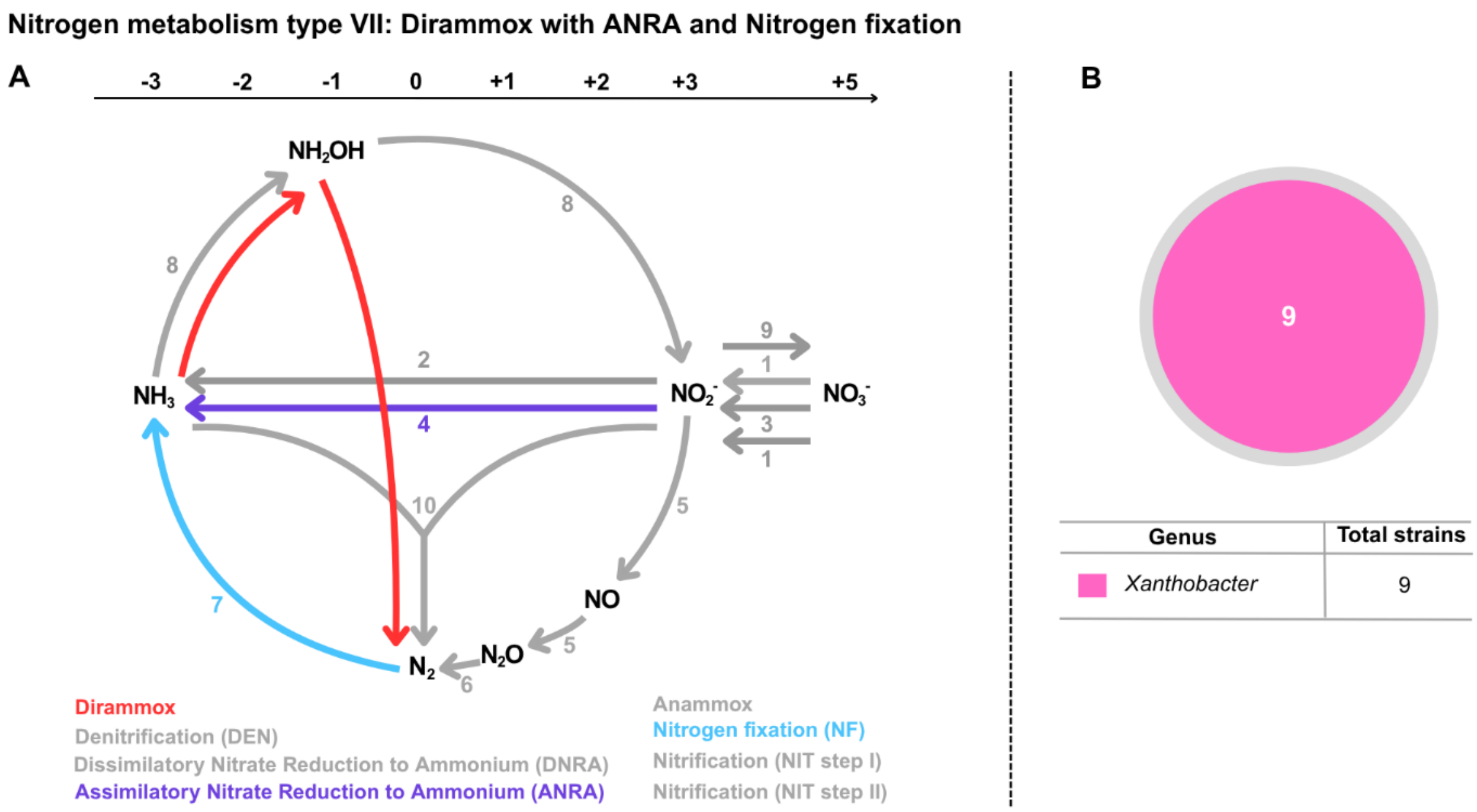

Fig. S32. Nitrogen metabolism type VII: genomes containing dirammox, ANRA, and nitrogen fixation pathways. (A) The dirammox pathway is shown in red, assimilatory nitrate reduction to ammonium is shown in purple, nitrogen fixation is shown in blue, and other nitrogen metabolism pathways are shown in gray. (B) Taxonomic distribution of type VII genomes.

Figure S33

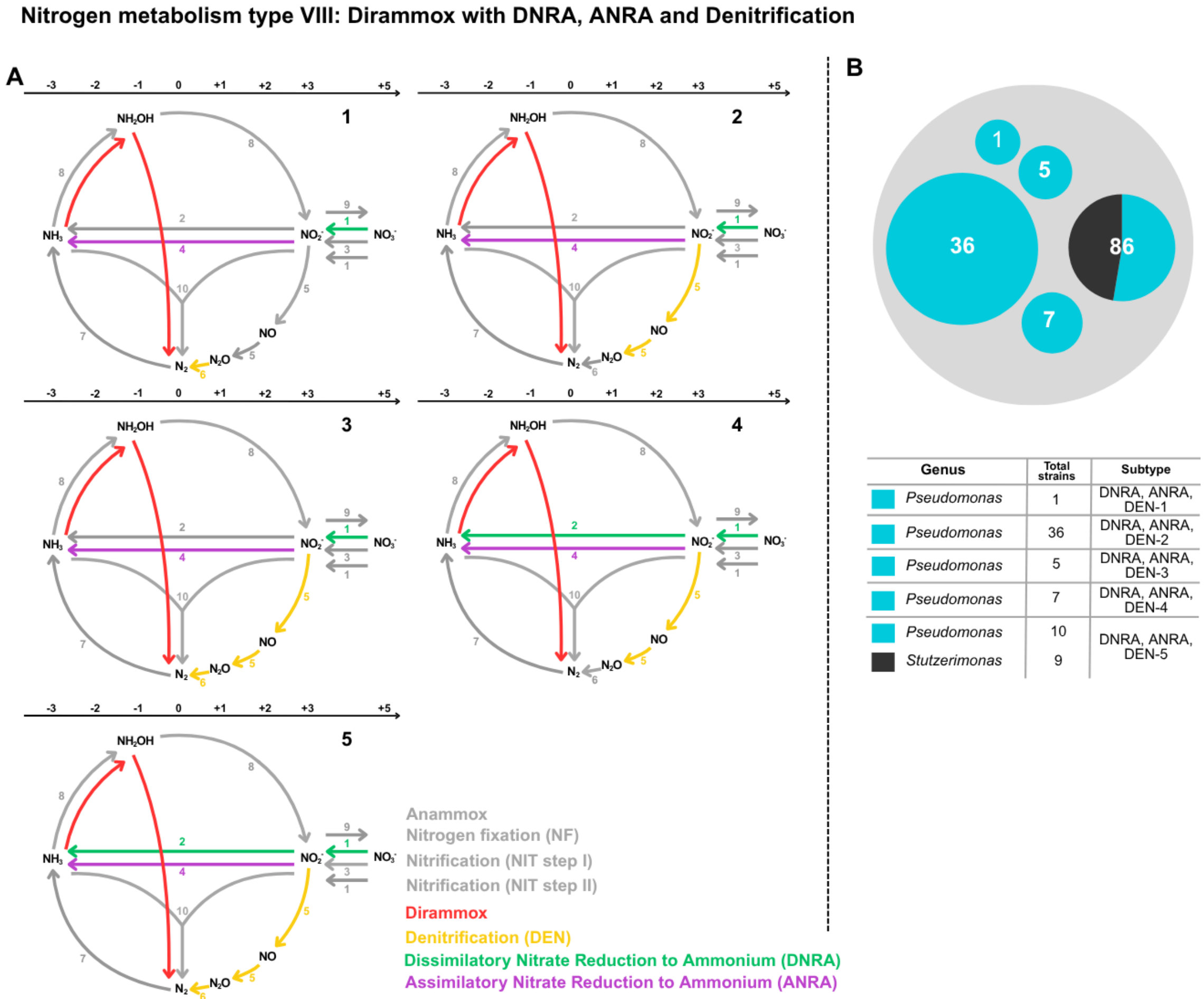

Genus	Total strains	Subtype
Pseudomonas	1	DNRA, ANRA, DEN-1
Pseudomonas	36	DNRA, ANRA, DEN-2
Pseudomonas	5	DNRA, ANRA, DEN-3
Pseudomonas	7	DNRA, ANRA, DEN-4
Pseudomonas	10	DNRA, ANRA, DEN-5
Stutzerimonas	9	

Fig. S33. Nitrogen metabolism type VIII: genomes containing dirammox, DNRA, ANRA, and denitrification pathways. (A) The dirammox pathway is shown in red, denitrification is shown in yellow, dissimilatory nitrate reduction to ammonium is shown in green, assimilatory nitrate reduction to ammonium is shown in purple, and other nitrogen metabolism pathways are shown in gray. (B) Taxonomic distribution and subtype composition of type VIII genomes.

Figure S34

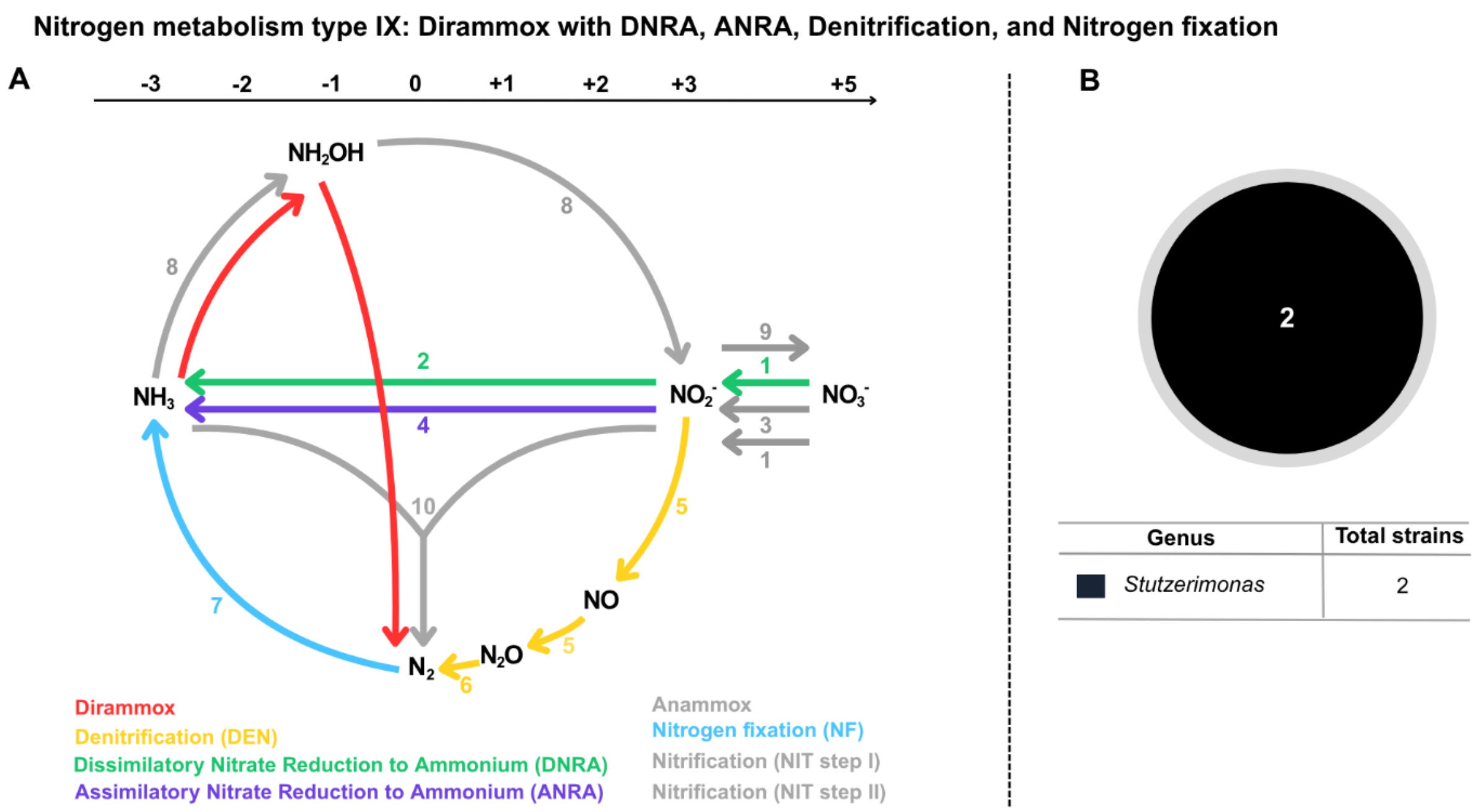

Fig. S34. Nitrogen metabolism type IX: genomes containing dirammox, DNRA, ANRA, denitrification, and nitrogen fixation pathways. (A) The dirammox pathway is shown in red, denitrification is shown in yellow, dissimilatory nitrate reduction to ammonium is shown in green, assimilatory nitrate reduction to ammonium is shown in purple, nitrogen fixation is shown in blue, and other nitrogen metabolism pathways are shown in gray. (B) Taxonomic distribution of type IX genomes.

Figure S35

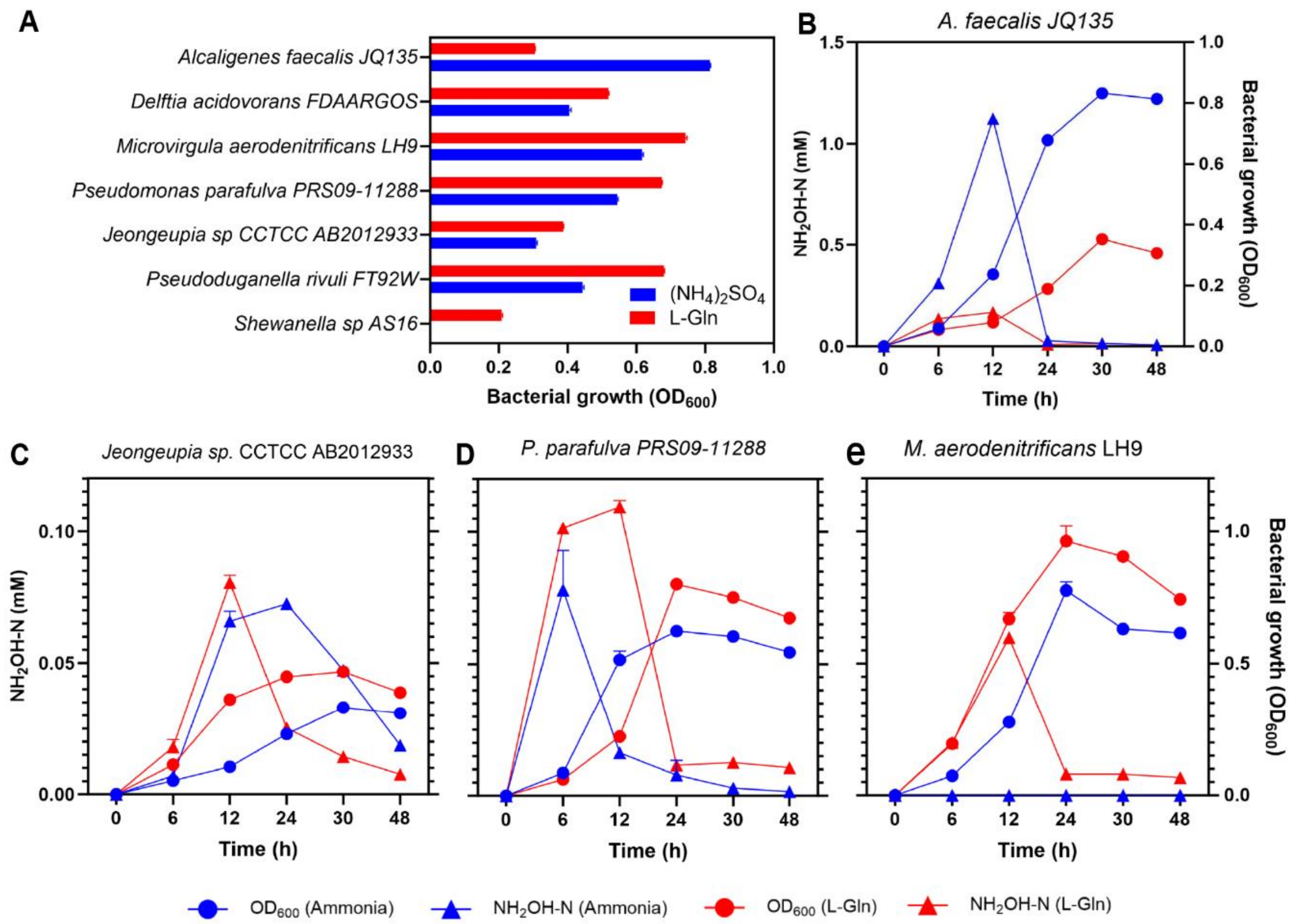

Fig. S35. Hydroxylamine accumulation and growth of seven wild-type strains on ammonia and L-glutamine as nitrogen sources. (**a**) Growth of seven wild type strains in the presence of ammonia and L-glutamine after 48 h of incubation. (**b–e**) Hydroxylamine accumulation and growth curves of *Alcaligenes faecalis* JQ135, *Jeongeupia* sp. CCTCC AB 2012933, *Pseudomonas parafulva* PRS09-11288, and *Microvirgula aerodenitrificans* LH9 were cultured with both nitrogen sources over 48 h. Blue and red bars represent hydroxylamine accumulation with ammonia and L-glutamine as substrate, respectively. Data represent the mean ± standard deviation (s.d.) of biological triplicates.

Figure S36

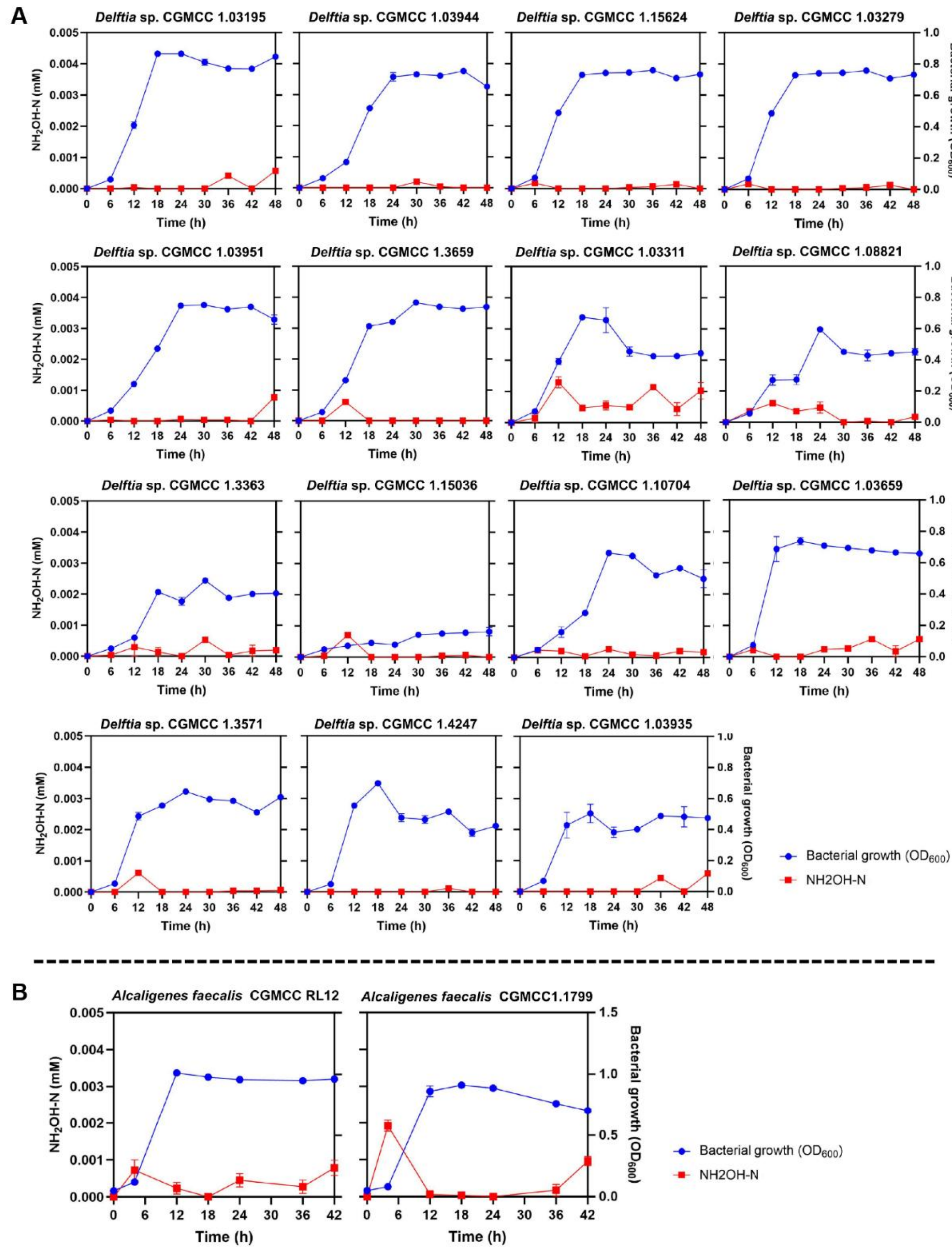

Fig. S36. Hydroxylamine accumulation and growth of *Delftia* and *Alcaligenes* strains on ammonia. (**a**) Hydroxylamine accumulation and growth curves of fifteen *Delftia* strains (**b**) two *Alcaligenes* strains.

Table S1. All strains were used in this study

Strains	Description	Source
Alcaligenes faecalis JQ135	Str^r; wild type	Lab stock
*Delftia acidovoran*s FDAARGOS 997	Wild type	China General Microbiological Culture Collection Center (CGMCC)
Microvirgula aerodenitrificans LH9	Wild type	Guizhou Medical University (GMU)
Pseudomonas parafulva PRS09-11288	Wild type	China General Microbiological Culture Collection Center (CGMCC)
Pseudoduganella rivuli FT92W	Wild type	Institute of Microbiology, Guangdong Academy of Sciences (GDIM)
Jeongeupia sp. CCTCC AB2012933	Wild type	China Center for Type Culture Collection (CCTCC)
Shewanella sp. AS16	Wild type	Jinan University (JNU)
Escherichia coli BW25113	Wild type, protein expression host	Lab stock
Recombinant strain JQ135	Cmr, *E. coli* BW25113 containing *dnfABC* gene cluster from *A. faecalis* JQ135	Cloned in this study
Recombinant strain FDAARGOS 997	*E. coli* BW25113 containing *dnfABC* gene cluster from D. acidovorans CGMCC 1.3363	Cloned in this study
Recombinant strain PRS09-11288	*E. coli* BW25113 containing *dnfABC* gene cluster from P. parafulva CGMCC 1.5632	Cloned in this study
Recombinant strain LH9	*E. coli* BW25113 containing *dnfABC* gene cluster from M. aerodenitrificans LH9	Cloned in this study
Recombinant strain CCTCC AB2012933	*E. coli* BW25113 containing *dnfABC* gene cluster from Jeon	Cloned in this study
Recombinant strain AS16	*E. coli* BW25113 containing *dnfABC* gene cluster from Shewanella sp. AS16	Cloned in this study
Recombinant strain FT92W	*E. coli* BW25113 containing *dnfABC* gene cluster from P. rivuli FT92W	Cloned in this study

Table S2. All plasmids and primers were used in this study

Plasmids/Primers	Description/Sequence (5' → 3')	Source/Description
DH5α	Cloning host	Lab stock
pBAD33	Gene expression plasmid	Lab stock
pBAD-Af	pBAD33 carrying *dnfABC* gene cluster of *A. faecalis* JQ135	In this study
pBAD-Da	pBAD33 carrying *dnfABC* gene cluster of *D. acidovorans* CGMCC 1.3363	In this study
pBAD-Pp	pBAD33 carrying *dnfABC* gene cluster of *P. parafulva* CGMCC 1.5632	In this study
pBAD-Ma	pBAD33 carrying *dnfABC* gene cluster of *M. aerodenitrificans* LH9	In this study
pBAD-Jc	pBAD33 carrying *dnfABC* gene cluster of *J. chitinilytica* KCTC 23701	In this study
pBAD-Sh	pBAD33 carrying *dnfABC* gene cluster of *Shewanella* sp. AS16	In this study
pBAD-Pr	pBAD33 carrying *dnfABC* gene cluster of *P. rivuli* FT92W	In this study
pBAD33-F	AAGCTTGGCTGTTTTGGCGGATG	For PCR of pBAD for cloning of dnfABC
pBAD33-R	CATGGTGAATTCCTCCTGCTAGCCCAAAAAAACGGGTA	
Af-ABC-F	AGCAGGAGGAATTCACCATGACAATCAAAAGCTACGAAACTG	To construct plasmid pBAD-Af
Af-ABC-R	CATCCGCCAAAACAGCCAAGCTTTCATGCAGCACAATCAGCGGA	
Pp-ABC-F	AGCAGGAGGAATTCACCATGCGTAATCAAACTTAACAACTACAA	To construct plasmid pBAD-Pp
Pp-ABC-R	CATCCGCCAAAACAGCCAAGCTTCTCCACCGAGATGAACCCT	
Da-ABC-F	AGCAGGAGGAATTCACCATGAATCTCGATATCTCTCGGAC	To construct plasmid pBAD-Da
Da-ABC-R	CATCCGCCAAAACAGCCAAGCTTGTACGACAACCATCTTCAACTCTC	
Ma-ABC-F	AGCAGGAGGAATTCACCATGAAACATTTGAATGCAGGTACC	To construct plasmid pBAD-Ma
Ma-ABC-R	CATCCGCCAAAACAGCCAAGCTTGAAACCACGTCCAGCAGATAC	
Jc-A-F	CTAGCGAATTCGAGCTAGGAGGAATTCACCATGGCTTT	To construct plasmid pBAD-Jc
Jc-A-R	GCATCATCGTGGTCATGACACCACCTCCAGAC	
Jc-B-F	TGTCATGACCACGATGATGCATG	
Jc-B-R	GGACTTCATAACACCTGCTCCTCT	
Jc-C-F	GAGGAGCAGGTGTTATGAAGTCCGTTCTTGCGA	
Jc-C-R	CCGCCAAAACAGCCATCATGCCGGCGAGGTTTG	
Sh-F	AGCAGGAGGAATTCACCATGTCAAGCGGCAATATTTCGCATATTGG	To construct plasmid pBAD-Sh
Sh-R	CATCCGCCAAAACAGCCAAGCTTCTAGGACAATGCTTGCAGCGC	
Pr_F	AGCAGGAGGAATTCACCATGGCGGTCTACGTGTCGAGC	To construct plasmid pBAD-Pr
Pr_R	CATCCGCCAAAACAGCCAAGCTTGAAGCCGCGTCAAAAATG	
