# Supplementary material for: Diversity of dirammox microorganisms
Source: mBio. 2026 Oct 15;17(11):e01645-26. doi: 10.1128/mbio.01645-26 (PMC13753816; doi:10.1128/mbio.01645-26)
Supplement: Supplementary file 3 [file mbio.01645-26-s0002.docx]

**Diversity of Dirammox Microorganisms**

Gantuya Khasbaatar^1,2#^, Qun Han^1,2#^, Xiao-Kang Wang^1,2#^, Lei Song^3^, Chang Liu^1,2^, Xin Sun^1,2^, Ze-Shen Liu^1^, Ying Liu^1^, Cheng-Ying Jiang^1^, Ji-Guo Qiu^4^, Shuang-Jiang Liu^1^, Yanning Zheng^1,2*^, De-Feng Li^1,2*^

^1^ State Key Laboratory of Microbial Diversity and Innovative Utilization, Institute of Microbiology, Chinese Academy of Sciences, Beijing 100101, China

^2^ College of Life Sciences, University of Chinese Academy of Sciences, Beijing 100049, China

^3^ China General Microbiological Culture Collection Center, Institute of Microbiology, Chinese Academy of Sciences, Beijing 100101, China

^4^ Key Laboratory of Agricultural and Environmental Microbiology, Ministry of Agriculture and Rural Affairs, College of Life Sciences, Nanjing Agricultural University, Nanjing 210095, China

^#^ Those authors contribute equally.

***Correspondences:**

De-Feng Li: lidefeng@im.ac.cn, Tel: +86-10-64807015.

Yanning Zheng: zhengyn@im.ac.cn, Tel: +86-10-64806943

**Running title: Diversity of Dirammox Microorganisms**

**This Supplementary Information file includes:** Results and Methods

Table of Contents

**Supplementary Results**

GC content deviation and MGE-associated genomic context of *dnfABC* loci……………….….3-4

**Supplementary Methods**

Phylogenetic analysis…………………………………………………………………….………..5

GC content and flanking Mobile Genetic Element (MGE) analysis………………………….…5-6

Bacterial strains, media, and cultivation conditions…………………………………………….….6

Cloning, expression, and functional assay of the *dnfABC* gene cluster…………………………6-7

**Supplementary Results**

**GC content deviation and MGE-associated genomic context of *dnfABC* loci**

To evaluate genomic signatures associated with *dnfABC* loci, GC content deviation was calculated for the complete *dnfABC* locus, the concatenated *dnfA*, *dnfB*, and *dnfC* CDSs, and each individual gene relative to the corresponding host genome background. Among the 690 complete *dnfABC* loci, 440 loci showed higher GC content than their host genomes, accounting for 63.8%, whereas 250 loci were GC-depleted, accounting for 36.2%. The mean signed GC deviation of the complete locus was 0.08 percentage points, while the mean absolute GC deviation was 2.35 percentage points. For the concatenated *dnfABC* CDSs, 453 clusters were GC-enriched relative to their host genomes, accounting for 65.7%, whereas 237 clusters were GC-depleted, accounting for 34.3%. The mean signed and absolute GC deviations of the concatenated CDSs were 0.11 and 2.41 percentage points, respectively.

At the individual gene level, *dnfA*, *dnfB*, and *dnfC* showed contrasting GC deviation patterns. *dnfA* was predominantly GC-depleted relative to the host genome background, with 645 of 690 sequences showing lower GC content, accounting for 93.5%. The mean signed GC deviation of *dnfA* was −3.10 percentage points, and the mean absolute deviation was 3.16 percentage points. In contrast, *dnfB* was mainly GC-enriched, with 574 of 690 sequences showing higher GC content than the host genome background, accounting for 83.2%. The mean signed and absolute GC deviations of *dnfB* were 2.06 and 3.76 percentage points, respectively. Similarly, *dnfC* was GC-enriched in 494 of 690 sequences, accounting for 71.6%, with mean signed and absolute deviations of 1.53 and 3.09 percentage points, respectively. These results indicate that the overall GC composition of the *dnfABC* cluster is shaped by contrasting gene-level GC patterns, with *dnfA* contributing mainly to GC depletion and *dnfB* and *dnfC* contributing mainly to GC enrichment.

Genus-level analysis showed substantial variation in GC deviation among *dnfABC*-harboring taxa. The largest mean absolute GC deviations of complete *dnfABC* loci were observed mainly in low-abundance genera, including *Burkholderia* (14.32 percentage points, n=2), *Xenorhabdus* (10.32, n=1), *Shewanella* (9.15, n=2), *Stutzerimonas* (7.91, n=11), *Verminephrobacter* (7.63, n=1), and *Xanthobacter* (4.62, n=9). These loci were generally GC-depleted relative to their corresponding host genomes. In contrast, the dominant *dnfABC*-harboring genera, including *Pseudomonas*, *Delftia*, and *Alcaligenes*, showed more moderate GC deviations, although individual clusters still exhibited intra-genus variability (Fig. S19-S22A).

To assess MGE-associated genomic context, protein-coding sequences located within 20 CDSs upstream and downstream of each *dnfABC* locus were examined. Strict MGE-related proteins were detected near 207 of 690 *dnfABC* clusters, accounting for 30.0%. In total, 470 strict MGE-related protein records were identified. Integrases were the most abundant MGE-related proteins, with 215 records, followed by transposases with 129 records, phage tail proteins with 117 records, and IS-family transposases with 96 records. Other MGE-related proteins, including phage terminases, holins, and portal proteins, were detected only rarely, with 6, 3, and 2 records, respectively. Weak mobility-associated signals were detected in 23 clusters, accounting for 3.3%, whereas non-MGE-like annotations were excluded from further analysis. The positions of strict MGE-related genes relative to *dnfABC* loci were also variable. MGE-related genes were detected upstream of *dnfABC* loci in 103 clusters and downstream in 118 clusters, whereas 14 clusters contained strict MGE-related genes on both sides. At the genus level, strict MGE signal frequencies differed substantially among taxa. Complete MGE association was observed in *Xanthobacter* (9/9, 100%), and relatively high frequencies were also detected in *Pseudoduganella* (2/3, 66.7%), *Herbaspirillum* (1/2, 50.0%), and *Pseudomonas* (162/362, 44.8%). In contrast, lower frequencies were observed in *Stutzerimonas* (3/11, 27.3%), *Jeongeupia* (1/5, 20.0%), *Streptomyces* (3/23, 13.0%), *Delftia* (14/115, 12.2%), and *Alcaligenes* (6/133, 4.5%). No strict MGE-related genes were detected in several genera, including *Microvirgula*, *Shewanella*, *Verminephrobacter*, and *Motilimonas* (Fig. S22B-S24).

**Supplementary Methods**

**Phylogenetic analysis**

Phylogenetic trees of DnfA, DnfB, and DnfC were reconstructed from amino acid sequences using the maximum likelihood method implemented in IQ-TREE2 [31]. To reduce redundancy and improve tree readability while retaining the diversity of gene cluster types and genus representation, sequences were dereplicated using a stratified CD-HIT strategy based on the previously defined classification of the *dnfABC* cluster. Briefly, the 690 genomes containing *dnfABC* had been assigned to seven cluster groups according to gene organization: Ia, Ib, Ic, II, III, IV, and V. Within each predefined cluster group, sequences were further stratified by genus, and CD-HIT was applied independently to each cluster group and genus stratum using a 95% sequence identity threshold. One representative sequence from each CD-HIT cluster was retained for downstream phylogenetic analysis. Multiple sequence alignments of representative DnfA, DnfB, and DnfC protein sequences were performed separately using MAFFT [32]. The resulting trees were visualized in iTOL (<https://itol.embl.de/>), and final illustrations with key annotations were prepared using Adobe Illustrator 2023 (Adobe Inc., San Jose, CA, USA).

**GC content and flanking Mobile Genetic Element (MGE) analysis**

GC content and MGE-associated genomic context were analyzed for 690 non-redundant *dnfABC*-positive genomes using the corresponding FNA, GFF, and FAA files. For each genome, the scaffold carrying the *dnfABC* cluster was identified, and whole-genome GC content was calculated from all A, T, G, and C bases. Nucleotide sequences of *dnfA*, *dnfB*, and *dnfC* were extracted based on GFF-derived coordinates and strand information. GC content was calculated for each gene, for the concatenated *dnfABC* CDSs, and for the complete *dnfABC* locus spanning from the minimum start to the maximum end coordinate, including intergenic regions. GC deviation was defined as the difference between the GC content of each target sequence and the corresponding whole-genome GC content. Signed and absolute GC deviations were summarized at both cluster and genus levels [33]. To assess MGE-associated genomic context, CDSs located within 20 genes upstream and downstream of each *dnfABC* locus on the same scaffold were extracted from GFF annotations. Flanking CDSs were screened based on product annotations and attribute fields. Strict MGE-related proteins, including transposases, IS-family transposases, integrases, prophage integrases, phage terminases, phage structural proteins, holins, and portal proteins, were used for the main statistics. Conjugative transfer proteins, plasmid maintenance proteins, and XerC/XerD recombinases were recorded as weak mobility-associated signals, whereas defense-, stress-, or host physiology-related proteins were excluded. Cluster- and genus-level MGE frequencies were calculated as the proportion of *dnfABC* loci containing at least one strict MGE-related protein within the 20-CDS flanking region [34].

**Bacterial strains, media, and cultivation conditions**

All bacterial strains used in this study are listed in Supplementary Table 1. The strains were cultivated aerobically in various media, including heterotrophic nitrification medium (HNM), Luria‐Bertani (LB), and Reasoner’s 2A (R2A) at 30°C and 160 rpm. HNM was prepared by dissolving the following components per liter: 4.72 g sodium succinate, 0.66 g (NH4)2SO4, 0.5 g KH2PO4, 0.2 g MgSO4·7H2O, 1.25 g Na2HPO4·12H2O, and 2 ml trace element solution. The trace solution consisted of Na₂EDTA 57.1 g/L, FeSO4·7H2O 5 g/L, ZnSO4·7H2O 3.9 g/L, MnCl₂·4H₂O 1 g/L, CoCl2·6H2O 1.6 g/L, CuSO4·5H2O 1.6 g/L, (NH4)6Mo7O24·4H2O 1.1 g/L and CaCl2·2H2O 7 g/L. In the modified HNM media, 10 mM of L-glutamine was used as a nitrogen source instead of ammonium [13-14, 16]. The initial pH of all media was adjusted to 7.0. Agar plates were prepared by adding agar to the medium in a 1.5% volume ratio.

**Cloning, expression, and functional assay of the *dnfABC* gene cluster**

Wild-type bacterial strains carrying the *dnfABC* cluster genes were activated in appropriate media, including LB and R2A, then overnight cultures (1% v/v) were inoculated into HNM or modified HNM medium and incubated at 30°C with shaking at 160 rpm. Growth (OD₆₀₀) and hydroxylamine concentrations were monitored using colorimetric assays [14-15, 37]. All measurements were performed with a VICTOR Nivo Multimode Microplate Reader (PerkinElmer). For heterologous expression, genomic DNA was extracted from a *dnfABC* containing wild-type strain using a commercial genomic DNA isolation kit (Tiangen DP302-2, Beijing). The *dnfABC* genes were amplified by PCR with gene-specific primers, purified, and cloned into the pBAD33 or DH5α plasmid (Amp^r^; Invitrogen, USA) using Gibson Assembly. The recombinant plasmid was transformed into competent *E. coli* BW25113 cells (TaKaRa, Japan) by heat shock [38]. All plasmids and primers are shown in Supplementary Table 2. Transformed colonies were selected on LB agar supplemented with chloramphenicol and verified by colony PCR and sequencing. Recombinant strains were induced with 5 mM L-arabinose until OD₆₀₀ reached ~0.5, and then harvested by centrifugation (6000 rpm, 10 min), washed with phosphate-buffered saline (PBS), and resuspended in HNM or modified HNM medium. Hydroxylamine accumulation was determined at 37 °C after 120 minutes, as previously described [14-15, 37]. All experiments were performed in triplicate, and results are presented as mean ± SD. Statistical analyses and data visualization were performed using GraphPad Prism version 10 (GraphPad Software, San Diego, CA, USA).
